# Supplementary material for: Computationally reproducing results from meta-analyses in ecology and evolutionary biology using shared code and data
Source: PLoS One. 2024 Mar 13;19(3):e0300333. doi: 10.1371/journal.pone.0300333 (PMC10936784; doi:10.1371/journal.pone.0300333)
Supplement: S1 Appendix — (PDF) [file pone.0300333.s001.pdf]

Appendix: Computationally reproducing results from meta-analyses in ecology and evolutionary biology using shared code and data

Steven Kambouris [1,2,*], **David P. Wilkinson** [1,2], **Eden T. Smith** [1,3], **Fiona Fidler** [1,2,3]
[1]MetaMelb Research Initiative, The University of Melbourne, Victoria, Australia
[2]School of Agriculture, Food and Ecosystem Sciences, The University of Melbourne, Victoria, Australia
[3]School of Historical and Philosophical Studies, The University of Melbourne, Victoria, Australia
[*]Correspondence: steven.kambouris@unimelb.edu.au

CONTENTS

1 Meta-analysis in ecology and evolutionary biology 3

2 Literature search 6

3 Review of journal policies on data and code sharing 21

4 Coding scheme for code and data sharing 24

5 Recording mentions of software used 25

6 Data and Code Sharing 29

7 Software mentioned in articles 35

8 Target Results 44

9 Reproducibility Reports 48

10 Reproducing target results when code not relevant 64

11 Revisiting the definition of reproducibility 71

1 META-ANALYSIS IN ECOLOGY AND EVOLUTIONARY BIOLOGY

Any study reporting numerical results (i.e., not just meta-analyses) can potentially be the subject of an attempt to computationally reproduce results, so why focusing on meta-analyses? First, there is a practical imperative here: narrowing the scope of this study makes it tractable. The ecology and evolution literature is vast and varied, and although the literature at large could be sampled to arrive at a manageable subset of articles to assess, the screening process to identify suitable candidate articles and exclude irrelevant ones would be arduous without some sort of guiding principle. In that sense, "meta-analysis" is just one of many potential ways to winnow down the literature, in that it is a quantitative method that produces numerical results that can (in principle) be subject to a computational reproducibility attempt. But still, why narrow the scope to meta-analyses in particular? To contextualise our answer to this, we start with a brief review of meta-analysis in ecology and evolutionary biology.

Meta-analysis in ecology and evolutionary biology

Meta-analysis, a set of statistical methods for combining the results of multiple studies, is a widely-used tool for research synthesis in medicine, the social sciences, and natural sciences (1). Meta-analysis has been used for decades in disciplines such as psychology, education, and especially medicine, where it has become a core tool for assessing the evidence of treatments, in particular via Cochrane systematic reviews (2; 3). In addition to an enormous literature on methods of meta-analysis, guidelines such as the Preferred Reporting Items for Systematic Reviews and Meta-Analyses (PRISMA) have been developed to standardise how meta-analyses are performed and reported (4; 5). PRISMA has been extended to be relevant to meta-analyses in ecology and evolutionary biology specifically (6).

Compared to some other disciplines, meta-analysis was adopted by ecology and evolutionary biology relatively recently, but has grown substantially, from a handful of meta-analyses published in the early 1990s to over 500 meta-analyses published in 2010 (1). In addition to meta-analyses themselves, there have been numerous methodological papers and handbooks covering how meta-analytical methods can be applied in circumstances specific to ecology/evolutionary biology (e.g., 7–10). Most relevantly for this paper, there have also been reviews of how meta-analyses have been conducted within the fields of ecology and evolutionary biology.

An early review of methods in meta-analysis (11) reviewed 29 meta-analyses published between 1991 and 1998, and is a useful baseline to track how methodology has progressed since the early years of meta-analysis in ecology. Overall, it was observed that techniques used in the medical meta-analysis literature had not been

adopted in ecology, in particular methods of assessing publication bias (only 34% of meta-analyses accounted for publication bias, and all calculated a Rosenthal fail-safe number; none used superior alternatives such as funnel plots, regression or the "trim and fill" method—refer to (12) for summaries of these methods). 76% of meta-analyses used the Q statistic to explore heterogeneity in effect sizes, and 17% included a sensitivity analysis of some kind. 28% of meta-analysis provided no information on how the primary studies were located. In terms of the effect sizes used by the meta-analyses, 55% used standardised mean differences, 31% used the Pearson correlation coefficient, and 7% used response ratios.

(12) conducted a survey of 100 "biological" meta-analyses (i.e., meta-analyses in ecology and evolutionary biology) published over 2009–2011. They found that only 17% controlled for phylogenetic relatedness between species, and 49% used methods to identify and/or assess the impact of publication bias (specifically, to identify publication bias, about 40% of these meta-analyses used funnel plots, about 10% used a correlation-based method, less than 10% used a quantile plot-based method, and about 5% used a regression-based method. For assessing the impact of publication bias, about 30% calculated Rosenthal fail-safe numbers and less than 10% used the "trim and fill" method). In terms of the effect sizes used by meta-analyses, about 60% used standardised mean differences, about 20% used correlation coefficients and the remainder used other measures.

(13) specifically addressed the point about a need for the term "meta-analysis" to be well-defined in the ecology and conservation biology literature. They examined 133 nominal meta-analyses, and applied a two-stage rating system based on the technical requirements for a meta-analysis according to the medical meta-analysis literature. They found that only 45% of the meta-analyses satisfied all requirements in the first stage of rating, and 25% of the meta-analyses satisfied none. In the second stage of rating which involved only 83 of the meta-analyses which had scored sufficiently highly in the first stage, only a single meta-analysis satisfied all second stage requirements, and 22% of the second stage meta-analyses satisfied none. The authors recommended that going forward, "meta-analyses" in ecology journals ought to include the seven technical requirements outlined in their review.

(14) performed a similar review to both (12) and (13), but focused on meta-analyses in plant ecology. They developed a 16-item rubric to assess the quality of meta-analytic methods, based on previous reviews of meta-analytic methods with some additions and refinements. Each item in the rubric listed meta-analyses that were exemplars of relevant method(s). They used a 14 of these items to assess the methods used in a sample of 322 meta-analyses in plant ecology. The results were mixed: although meta-analyses in plant ecology were highly likely to list all primary studies included in the analysis (87% of meta-analyses in the sample) or

explore caused of heterogeneity in results (89%), only 32% reported the full details of their literature searches, only 31% considered publication bias, and only 25% conducted a sensitivity analysis. 5% of meta-analyses considered changes in study effect sizes over time, and 11% took phylogenetic relatedness into account.

(15) used a 17-item rubric very similar to (14) to evaluate the methods used by 18 meta-analyses published in the journal *Molecular Ecology*. The goal of this paper was to formalise the definition of meta-analysis for editors, authors, reviewers, and readers of the journal. They found that only 22% of studies met the standard expected for an effective meta-analysis, which required satisfying at least 15 of the 17 items, and 56% of meta-analyses satisfied 9 or fewer of the 17 items. The adherence to the different methods was mixed: While 100% of the meta-analyses provided a list of the primary studies included and documented the meta-data extracted from each, and 94% mentioned the inclusion/exclusion criteria used for selected studies, 50% included details of the literature search terms used and only 22% provided details of both the databases searched and dates the searches were conducted. Only 33% of meta-analyses took publication bias into account, 22% quantified the heterogeneity between effect sizes, and only 33% explicitly reported whether they were using a fixed effect or random effects model.

The common theme of heterogeneity in the methods labelled as "meta-analysis" in the ecology literature has led to the emergence of what might be called the "formal" (13; 14) or "narrow" (16) definition of meta-analysis: (14) define meta-analysis "a set of statistical methods for combining outcomes (effect sizes) across different data sets addressing the same research question to examine patterns of response across these data sets and sources of heterogeneity in outcomes", although they do also note that there is no single agreed-upon checklist for assessing whether a given meta-analysis is using the correct methods for this purpose. What does seem clear is that the methods and procedures of the formal/narrow meta-analysis are those mentioned in the checklists/rubrics/rating systems of (13), (14), and (15).

Focusing on meta-analysis in this study

The first and primary reason for choosing to focus on meta-analysis is this: despite the findings outlined in the previous section, meta-analyses are (relatively) uniform in their statistical methods and data, and so restricting the study to meta-analyses allows for the assessment of "like" studies. This has a few different dimensions that speak to the tractability of the study:

- The great variety in quantitative and statistical methods employed across the entire ecology and evolution literature (with the accompanying variety in computational resource requirements) means that failure to computationally reproduce one study but not another could be a result of radically differ-

ent scales of computational requirements, which is a confounding factor we'd like to avoid as much as possible, due to limited resources. Potential ways of dealing with this (e.g., screening articles to preclude studies with "too high" computational resource requirements) seem too subjective and difficult to operationalise. Choosing a single type of study, meta-analysis, acts to reduce the likely variation in computational resource requirements.

- In general, meta-analytic models are fitted using relatively small data sets (in the order of tens or hundreds, perhaps thousands, of data points as opposed to "big data" with millions of data points) and require modest computational resources (i.e., can be easily run on a desktop or laptop computer with no high performance computing resources required).

- Meta-analyses in particular benefit from the existence of standards for reporting, e.g., PRISMA. Relevantly for this study, this includes standards around the reporting/sharing of data. While a given meta-analysis may not be obliged to strictly adhere to all PRIMSA reporting guidelines, the existence of such guidelines makes it more likely that different studies can be assessed on a like basis than if no such guidelines or standards existed.

The second reason is, as mentioned earlier, meta-analysis has become an important part of the fields of ecology and evolution. To the extent that meta-analyses become regarded (for better or worse) as a higher standard of evidence, it commensurately raises the stakes of meta-analytic results. In that context, being able to assure the results of meta-analyses through computational reproduction has some value.

2 LITERATURE SEARCH

We set about curating a set of meta-analyses to survey by conducting a Scopus abstract and citation database search (we accessed the Scopus database via the University of Melbourne library's subscription). The search query, conducted on 20th December 2017, searched article titles, abstracts, and keywords for the string "meta-anal*", subject to two constraints. The first constraint restricted results to articles published between 2015 and 2017, inclusive. The second constraint restricted results to articles published in one of 21 ecology and evolution journal titles (identified by ISSN). The journal titles included are as follows: *The American Naturalist*, *Animal Behaviour*, *Behavioral Ecology*, *Behavioral Ecology and Sociobiology*, *Biological Reviews*, *Ecological Applications*, *Ecological Monographs*, *Ecology*, *Ecology Letters*, *Evolution*, *Evolutionary Ecology*, *Functional Ecology*, *Journal of Animal Ecology*, *Journal of Applied Ecology*, *Journal of Ecology*, *Journal of Evolutionary Biology*, *Molecular Ecology*, *New Phytologist*, *Oecologia*, *Oikos*, *Quarterly Review of Biology*.

The Scopus search string used was as follows:

```
TITLE-ABS-KEY ( meta-anal* ) AND ( PUBYEAR = 2015
OR PUBYEAR = 2016 OR PUBYEAR = 2017 )
AND ISSN ( 0003-0147 OR 0003-3472 OR 1045-2249 OR 0340-5443
OR 1464-7931 OR 1051-0761 OR 0012-9615 OR 0012-9658
OR 1461-023x OR 0014-3820 OR 0269-7653 OR 0269-8463
OR 0021-8790 OR 0021-8901 OR 0022-0477 OR 1010-061x
OR 0962-1083 OR 0028-646x OR 0029-8549 OR 0030-1299
OR 0033-5770 )
```

This list of ecology and evolution journal titles is the same as used for the survey of meta-analyses conducted in (12). This choice was made to (i) be assured of searching journals that actively published meta-analyses, and (ii) keep the study tractable: (12) yielded 390 studies from their three-year (2009–11) search of these journal titles and kept the 100 most recent meta-analyses, so that gave an indication of the approximate number of meta-analysis studies we would need to review. It is unclear if this set of journal titles can be considered a "representative" sample of all ecology and evolutionary biology journals; one obvious factor is that not all journals would necessarily consider meta-analyses to be within their scope. However, it seems clear that the list of journals used for this study is not particularly aberrant, at least: for example, (17) reviewed the data and code release policies of 96 "ecology" journals indexed by Web of Science, and the list of 96 journals reviewed includes 17 of the 21 titles surveyed by (12).

Identifying meta-analyses

The search results returned articles which contained the string "meta-anal*" somewhere in the article's title, abstract, or list of keywords. However, not all such articles will necessarily be meta-analyses. The next step was to screen the articles to obtain a sample of "meta-analyses". As the review of the ecological meta-analysis methodology literature foreshadowed, this was not straightforward.

The articles were screened using a two-step process: first, some types of articles were checked for and when found, put aside. These article types were (i) errata or corrigenda notices, and (ii) letters or comments in reply to a previously published article. Since errata and comments rely heavily on the context provided by the article they are in reference to (which may or may not be a meta-analysis, and which may or may not be in scope of the literature search), they were considered not suitable to include as "meta-analyses". We considered these article types to be straightforward to identify (due to clear cues in their title, and other contextual clues such as being included in a comments/letters section of a journal issue), and so removed them from consideration without a formal review of their contents.

The second step involved evaluating the remaining articles in the following way: rather than checking they meet a particular set of methodological requirements, meta-analyses were identified by confirming that an article merely includes a *claim* that it is a meta-analysis (or that a meta-analysis was conducted, or words to that effect) or not. This approach to identifying meta-analyses was intended to be as generous as possible and methodologically agnostic.

Identifying a claim that an article is/conducts a meta-analysis still requires judgment and interpretation, and is subjective. To make the claim identification process transparent, we constructed and employed a simple coding scheme with eight items to summarise the "evidence" in support of each article claiming to be a meta-analysis. The coding scheme is outlined in Table 1. This scheme records the use of the term "meta-analysis" in crucial places in the article (title, abstract, keywords if the article includes them), as well as the quoted text of any actual claim found within the body of the article text.

For items 1–4 and 7, the value "Y" indicates an unambiguous "yes" to the question/contention posed in the column "Description" of Table 1, and the value "N" indicates an unambiguous "no". For items 1–4, the value "U" was available to indicate situations where the mention of "meta-analysis" was somehow unclear. For item 3 only, the value "N/A" was used to indicate that an article did not include any keywords.

Items 5 and 6 record the most substantive piece of evidence: text, directly quoted from the article, which contains the claim to be a meta-analysis (if the claim can be found). Item 7 contains the final judgment of whether the article can be considered to include a claim or not (either yes "Y" or no "N"), and item 8 records any additional notes about the judgment.

All articles remaining after the first step were coded using this scheme. In practice, this meant searching the text of each article for the string "meta" (this word fragment was chosen to avoid issues with the matching of the hyphen in "meta-analysis"), and reviewing all matches in order to answer the coding scheme items.

Articles were considered as claiming to be a meta-analysis if the value of item 7 (Claim in article) in the coding scheme was "Y" . Articles found not to include such a claim (a value of "N" for item 7) were put aside.

The final set of ecology and evolutionary biology meta-analyses, to be the basis of the rest of this study, is simply the set of 177 articles coded as containing claims to be meta-analyses. The bibliographic details of all 177 meta-analysis articles are listed in Table 2.

Index	Field	Values	Description
1	Claim in title	Y, N, U	Does the article include the term "meta-analysis" in its title?
2	Claim in abstract	Y, N, U	Does the article include the term "meta-analysis" in its abstract?
3	Claim in keywords	Y, N, U, N/A	If applicable, is "meta-analysis" one of the article's keywords?
4	Claim in body text	Y, N, U	Does the article body text contain a claim to be a meta-analysis?
5	Quote of claim	open text	The actual text of the claim as it appears in the article.
6	Quote page number	open text	Page number(s) the quote appears on.
7	Claim in article	Y, N	An overall judgment of whether or not the article claims to be a meta-analysis.
8	Notes	open text	Any additional notes about the article's meta-analysis claim status.

Table 1: The eight item coding scheme used for determining whether an article claims to be a meta-analysis. In the Values column, "Y" indicates "yes", "N" indicates "no", "U" indicates "unclear", and "N/A" indicates "not applicable".

Table 2: References for all 177 meta-analysis articles in the data set used in this study.

ID	Study
MA001	Bowles TM, Jackson LE, Loeher M, Cavagnaro TR. Ecological intensification and arbuscular mycorrhizas: a meta-analysis of tillage and cover crop effects. Journal of Applied Ecology. 2017;54(6):1785–1793. doi:10.1111/1365-2664.12815
MA003	Mori AS, Tatsumi S, Gustafsson L. Landscape properties affect biodiversity response to retention approaches in forestry. Journal of Applied Ecology. 2017;54(6):1627–1637. doi:10.1111/1365-2664.12888
MA005	Charlebois JA, Sargent RD. No consistent pollinator-mediated impacts of alien plants on natives. Ecology Letters. 2017;20(11):1479–1490. doi:10.1111/ele.12831
MA006	Martin-StPaul N, Delzon S, Cochard H. Plant resistance to drought depends on timely stomatal closure. Ecology Letters. 2017;20(11):1437–1447. doi:10.1111/ele.12851
MA009	Romano A, Saino N, Møller AP. Viability and expression of sexual ornaments in the barn swallow Hirundo rustica: a meta-analysis. Journal of Evolutionary Biology. 2017;30(10):1929–1935. doi:10.1111/jeb.13151
MA010	Davidson KE, Fowler MS, Skov MW, Doerr SH, Beaumont N, Griffin JN. Livestock grazing alters multiple ecosystem properties and services in salt marshes: a meta-analysis. Journal of Applied Ecology. 2017;54(5):1395–1405. doi:10.1111/1365-2664.12892
MA011	Grant JWA, Weir LK, Steingrímsson SÓ. Territory size decreases minimally with increasing food abundance in stream salmonids: Implications for population regulation. Journal of Animal Ecology. 2017;86(6):1308–1316. doi:10.1111/1365-2656.12737
MA013	Horswill C, O'Brien SH, Robinson RA. Density dependence and marine bird populations: are wind farm assessments precautionary? Journal of Applied Ecology. 2017;54(5):1406–1414. doi:10.1111/1365-2664.12841

ID	Study
MA014	Auer SK, Killen SS, Rezende EL. Resting vs. active: a meta-analysis of the intra- and inter-specific associations between minimum, sustained, and maximum metabolic rates in vertebrates. Functional Ecology. 2017;31(9):1728–1738. doi:10.1111/1365-2435.12879
MA015	Buchanan AL, Hermann SL, Lund M, Szendrei Z. A meta-analysis of non-consumptive predator effects in arthropods: the influence of organismal and environmental characteristics. Oikos. 2017;126(9):1233–1240. doi:10.1111/oik.04384
MA016	Xu X, Medvigy D, Joseph Wright S, Kitajima K, Wu J, Albert LP, et al. Variations of leaf longevity in tropical moist forests predicted by a trait-driven carbon optimality model. Ecology Letters. 2017;20(9):1097–1106. doi:10.1111/ele.12804
MA017	Soria M, Leigh C, Datry T, Bini LM, Bonada N. Biodiversity in perennial and intermittent rivers: a meta-analysis. Oikos. 2017;126(8):1078–1089. doi:10.1111/oik.04118
MA018	Delavaux CS, Smith-Ramesh LM, Kuebbing SE. Beyond nutrients: a meta-analysis of the diverse effects of arbuscular mycorrhizal fungi on plants and soils. Ecology. 2017;98(8):2111–2119. doi:10.1002/ecy.1892
MA019	LaManna JA, Martin TE. Logging impacts on avian species richness and composition differ across latitudes and foraging and breeding habitat preferences. Biological Reviews. 2017;92(3):1657–1674. doi:10.1111/brv.12300
MA020	Leal LC, Peixoto PEC. Decreasing water availability across the globe improves the effectiveness of protective ant–plant mutualisms: a meta-analysis. Biological Reviews. 2017;92(3):1785–1794. doi:10.1111/brv.12307
MA021	Romano A, Costanzo A, Rubolini D, Saino N, Møller AP. Geographical and seasonal variation in the intensity of sexual selection in the barn swallow Hirundo rustica: a meta-analysis. Biological Reviews. 2017;92(3):1582–1600. doi:10.1111/brv.12297
MA022	Anthelme F, Meneses RI, Valero NNH, Pozo P, Dangles O. Fine nurse variations explain discrepancies in the stress-interaction relationship in alpine regions. Oikos. 2017;126(8):1173–1183. doi:10.1111/oik.04248
MA023	Hindrikson M, Remm J, Pilot M, Godinho R, Stronen AV, Baltrūnaité L, et al. Wolf population genetics in Europe: a systematic review, meta-analysis and suggestions for conservation and management. Biological Reviews. 2017;92(3):1601–1629. doi:10.1111/brv.12298
MA024	Knapp JL, Bartlett LJ, Osborne JL. Re-evaluating strategies for pollinator-dependent crops: How useful is parthenocarpy? Journal of Applied Ecology. 2017;54(4):1171–1179. doi:10.1111/1365-2664.12813
MA025	Gázquez A, Beemster GTS. What determines organ size differences between species? A meta-analysis of the cellular basis. New Phytologist. 2017;215(1):299–308. doi:10.1111/nph.14573
MA026	Hendershot JN, Read QD, Henning JA, Sanders NJ, Classen AT. Consistently inconsistent drivers of microbial diversity and abundance at macroecological scales. Ecology. 2017;98(7):1757–1763. doi:10.1002/ecy.1829
MA027	Farji-Brener AG, Werenkraut V. The effects of ant nests on soil fertility and plant performance: a meta-analysis. Journal of Animal Ecology. 2017;86(4):866–877. doi:10.1111/1365-2656.12672

ID	Study
MA028	Hitchcock DJ, Varpe Ø, Andersen T, Borgå K. Effects of reproductive strategies on pollutant concentrations in pinnipeds: a meta-analysis. Oikos. 2017;126(6):772–781. doi:10.1111/oik.03955
MA029	Miller SE, Barrueto M, Schluter D. A comparative analysis of experimental selection on the stickleback pelvis. Journal of Evolutionary Biology. 2017;30(6):1165–1176. doi:10.1111/jeb.13085
MA031	Rowiński PK, Rogell B. Environmental stress correlates with increases in both genetic and residual variances: A meta-analysis of animal studies. Evolution. 2017;71(5):1339–1351. doi:10.1111/evo.13201
MA033	Yue K, Fornara DA, Yang W, Peng Y, Peng C, Liu Z, et al. Influence of multiple global change drivers on terrestrial carbon storage: additive effects are common. Ecology Letters. 2017;20(5):663–672. doi:10.1111/ele.12767
MA035	Wood KA, Ponting J, D'Costa N, Newth JL, Rose PE, Glazov P, et al. Understanding intrinsic and extrinsic drivers of aggressive behaviour in waterbird assemblages: a meta-analysis. Animal Behaviour. 2017;126:209–216. doi:10.1016/j.anbehav.2017.02.008
MA036	Greenwood S, Ruiz-Benito P, Martínez-Vilalta J, Lloret F, Kitzberger T, Allen CD, et al. Tree mortality across biomes is promoted by drought intensity, lower wood density and higher specific leaf area. Ecology Letters. 2017;20(4):539–553. doi:10.1111/ele.12748
MA037	Martin PA, Newton AC, Bullock JM. Impacts of invasive plants on carbon pools depend on both species' traits and local climate. Ecology. 2017;98(4):1026–1035. doi:10.1002/ecy.1711
MA038	Holtmann B, Lagisz M, Nakagawa S. Metabolic rates, and not hormone levels, are a likely mediator of between-individual differences in behaviour: a meta-analysis. Functional Ecology. 2017;31(3):685–696. doi:10.1111/1365-2435.12779
MA039	Catano CP, Dickson TL, Myers JA. Dispersal and neutral sampling mediate contingent effects of disturbance on plant beta-diversity: a meta-analysis. Ecology Letters. 2017;20(3):347–356. doi:10.1111/ele.12733
MA040	Poulin R, Pérez-Ponce de León G. Global analysis reveals that cryptic diversity is linked with habitat but not mode of life. Journal of Evolutionary Biology. 2017;30(3):641–649. doi:10.1111/jeb.13034
MA041	Usui T, Butchart SHM, Phillimore AB. Temporal shifts and temperature sensitivity of avian spring migratory phenology: a phylogenetic meta-analysis. Journal of Animal Ecology. 2017;86(2):250–261. doi:10.1111/1365-2656.12612
MA042	Hrycik AR, Almeida LZ, Höök TO. Sub-lethal effects on fish provide insight into a biologically-relevant threshold of hypoxia. Oikos. 2017;126(3):307–317. doi:10.1111/oik.03678
MA044	Rossetti MR, Tscharntke T, Aguilar R, Batáry P. Responses of insect herbivores and herbivory to habitat fragmentation: a hierarchical meta-analysis. Ecology Letters. 2017;20(2):264–272. doi:10.1111/ele.12723
MA045	Sorenson GH, Dey CJ, Madliger CL, Love OP. Effectiveness of baseline corticosterone as a monitoring tool for fitness: a meta-analysis in seabirds. Oecologia. 2017;183(2):353–365. doi:10.1007/s00442-016-3774-3
MA046	Foo YZ, Nakagawa S, Rhodes G, Simmons LW. The effects of sex hormones on immune function: a meta-analysis. Biological Reviews. 2017;92(1):551–571. doi:10.1111/brv.12243

ID	Study
MA048	Hossie T, Landolt K, Murray DL. Determinants and co-expression of anti-predator responses in amphibian tadpoles: a meta-analysis. Oikos. 2017;126(2):n/a–n/a. doi:10.1111/oik.03305
MA049	Booksmythe I, Mautz B, Davis J, Nakagawa S, Jennions MD. Facultative adjustment of the offspring sex ratio and male attractiveness: a systematic review and meta-analysis. Biological Reviews. 2017;92(1):108–134. doi:10.1111/brv.12220
MA052	Ameye M, Allmann S, Verwaeren J, Smagghe G, Haesaert G, Schuurink RC, et al. Green leaf volatile production by plants: a meta-analysis. New Phytologist. 2017; p. n/a–n/a. doi:10.1111/nph.14671
MA053	Vidal MC, Murphy SM. Bottom-up vs. top-down effects on terrestrial insect herbivores: a meta-analysis. Ecology Letters. 2018;21(1):138–150. doi:10.1111/ele.12874
MA054	Deng Q, McMahon DE, Xiang Y, Yu CL, Jackson RB, Hui D. A global meta-analysis of soil phosphorus dynamics after afforestation. New Phytologist. 2017;213(1):181–192. doi:10.1111/nph.14119
MA055	Lee MR, Bernhardt ES, van Bodegom PM, Cornelissen JHC, Kattge J, Laughlin DC, et al. Invasive species' leaf traits and dissimilarity from natives shape their impact on nitrogen cycling: a meta-analysis. New Phytologist. 2017;213(1):128–139. doi:10.1111/nph.14115
MA056	Zvereva EL, Zverev V, Kruglova OY, Kozlov MV. Strategies of chemical anti-predator defences in leaf beetles: is sequestration of plant toxins less costly than de novo synthesis? Oecologia. 2017;183(1):93–106. doi:10.1007/s00442-016-3743-x
MA057	Xiao Z, Wang X, Koricheva J, Kergunteuil A, Le Bayon RC, Liu M, et al. Earthworms affect plant growth and resistance against herbivores: A meta-analysis. Functional Ecology. 2018;32(1):150–160. doi:10.1111/1365-2435.12969
MA058	Camenzind T, Hättenschwiler S, Treseder KK, Lehmann A, Rillig MC. Nutrient limitation of soil microbial processes in tropical forests. Ecological Monographs. 2018;88(1):4–21. doi:10.1002/ecm.1279
MA059	Yang H, Zhang Q, Koide RT, Hoeksema JD, Tang J, Bian X, et al. Taxonomic resolution is a determinant of biodiversity effects in arbuscular mycorrhizal fungal communities. Journal of Ecology. 2017;105(1):219–228. doi:10.1111/1365-2745.12655
MA060	Winternitz J, Abbate JL, Huchard E, Havlíček J, Garamszegi LZ. Patterns of MHC-dependent mate selection in humans and nonhuman primates: a meta-analysis. Molecular Ecology. 2017;26(2):668–688. doi:10.1111/mec.13920
MA061	Molnar JL, Diogo R, Hutchinson JR, Pierce SE. Reconstructing pectoral appendicular muscle anatomy in fossil fish and tetrapods over the fins-to-limbs transition. Biological Reviews. 2017; p. n/a–n/a. doi:10.1111/brv.12386
MA062	Grueber CE, Gray LJ, Morris KM, Simpson SJ, Senior AM. Intergenerational effects of nutrition on immunity: a systematic review and meta-analysis. Biological Reviews. 2018;93(2):1108–1124. doi:10.1111/brv.12387

ID	Study
MA063	Gruner DS, Bracken MES, Berger SA, Eriksson BK, Gamfeldt L, Matthiessen B, et al. Effects of experimental warming on biodiversity depend on ecosystem type and local species composition. Oikos. 2016;126(1):8–17. doi:10.1111/oik.03688
MA064	Dynarski KA, Houlton BZ. Nutrient limitation of terrestrial free-living nitrogen fixation. New Phytologist. 2018;217(3):1050–1061. doi:10.1111/nph.14905
MA065	Noble DWA, Stenhouse V, Schwanz LE. Developmental temperatures and phenotypic plasticity in reptiles: a systematic review and meta-analysis. Biological Reviews. 2018;93(1):72–97. doi:10.1111/brv.12333
MA066	Junker RR, Kuppler J, Amo L, Blande JD, Borges RM, van Dam NM, et al. Covariation and phenotypic integration in chemical communication displays: biosynthetic constraints and eco-evolutionary implications. New Phytologist. 2017; p. n/a–n/a. doi:10.1111/nph.14505
MA067	Risely A, Klaassen M, Hoye BJ. Migratory animals feel the cost of getting sick: A meta-analysis across species. Journal of Animal Ecology. 2017;87(1):301–314. doi:10.1111/1365-2656.12766
MA068	Ronget V, Gaillard J, Coulson T, Garratt M, Gueyffier F, Lega J, et al. Causes and consequences of variation in offspring body mass: meta-analyses in birds and mammals. Biological Reviews. 2017;93(1):1–27. doi:10.1111/brv.12329
MA069	Rohner PT, Teder T, Esperk T, Lüpold S, Blanckenhorn WU. The evolution of male-biased sexual size dimorphism is associated with increased body size plasticity in males. Functional Ecology. 2017;In press:n/a–n/a. doi:10.1111/1365-2435.13004
MA070	Goodell K, Parker IM. Invasion of a dominant floral resource: effects on the floral community and pollination of native plants. Ecology. 2017;98(1):57–69. doi:10.1002/ecy.1639
MA071	Sievers M, Hale R, Parris KM, Swearer SE. Impacts of human-induced environmental change in wetlands on aquatic animals. Biological Reviews. 2017;93(1):529–554. doi:10.1111/brv.12358
MA074	Harts AMF, Booksmythe I, Jennions MD. Mate guarding and frequent copulation in birds: A meta-analysis of their relationship to paternity and male phenotype. Evolution. 2016;70(12):2789–2808. doi:10.1111/evo.13081
MA075	Poorter H, Fiorani F, Pieruschka R, Wojciechowski T, Putten WH, Kleyer M, et al. Pampered inside, pestered outside? Differences and similarities between plants growing in controlled conditions and in the field. New Phytologist. 2016;212(4):838–855. doi:10.1111/nph.14243
MA076	Edge CB, Houlahan JE, Jackson DA, Fortin M. The response of amphibian larvae to environmental change is both consistent and variable. Oikos. 2016;125(12):1700–1711. doi:10.1111/oik.03166
MA077	Gill AL, Finzi AC. Belowground carbon flux links biogeochemical cycles and resource-use efficiency at the global scale. Ecology Letters. 2016;19(12):1419–1428. doi:10.1111/ele.12690
MA078	Buoro M, Olden JD, Cucherousset J. Global Salmonidae introductions reveal stronger ecological effects of changing intraspecific compared to interspecific diversity. Ecology Letters. 2016;19(11):1363–1371. doi:10.1111/ele.12673

ID	Study
MA079	Besson AA, Lagisz M, Senior AM, Hector KL, Nakagawa S. Effect of maternal diet on offspring coping styles in rodents: a systematic review and meta-analysis. Biological Reviews. 2016;91(4):1065–1080. doi:10.1111/brv.12210
MA080	Flick AJ, Acevedo MA, Elderd BD. The negative effects of pathogen-infected prey on predators: a meta-analysis. Oikos. 2016;125(11):1554–1560. doi:10.1111/oik.03458
MA081	Jaffé R, Pope N, Acosta AL, Alves DA, Arias MC, De la Rúa P, et al. Beekeeping practices and geographic distance, not land use, drive gene flow across tropical bees. Molecular Ecology. 2016;25(21):5345–5358. doi:10.1111/mec.13852
MA082	Tsen EWJ, Sitzia T, Webber BL. To core, or not to core: the impact of coring on tree health and a best-practice framework for collecting dendrochronological information from living trees. Biological Reviews. 2016;91(4):899–924. doi:10.1111/brv.12200
MA090	Derroire G, Balvanera P, Castellanos-Castro C, Decocq G, Kennard DK, Lebrija-Trejos E, et al. Resilience of tropical dry forests – a meta-analysis of changes in species diversity and composition during secondary succession. Oikos. 2016;125(10):1386–1397. doi:10.1111/oik.03229
MA091	Lemoine NP, Hoffman A, Felton AJ, Baur L, Chaves F, Gray J, et al. Underappreciated problems of low replication in ecological field studies. Ecology. 2016;97(10):2554–2561. doi:10.1002/ecy.1506
MA092	Xu X, Medvigy D, Powers JS, Becknell JM, Guan K. Diversity in plant hydraulic traits explains seasonal and inter-annual variations of vegetation dynamics in seasonally dry tropical forests. New Phytologist. 2016;212(1):80–95. doi:10.1111/nph.14009
MA093	Hidding B, Bakker ES, Hootsmans MJM, Hilt S. Synergy between shading and herbivory triggers macrophyte loss and regime shifts in aquatic systems. Oikos. 2016;125(10):1489–1495. doi:10.1111/oik.03104
MA094	Turney S, Buddle CM. Pyramids of species richness: the determinants and distribution of species diversity across trophic levels. Oikos. 2016;125(9):1224–1232. doi:10.1111/oik.03404
MA095	Gibert A, Gray EF, Westoby M, Wright IJ, Falster DS. On the link between functional traits and growth rate: meta-analysis shows effects change with plant size, as predicted. Journal of Ecology. 2016;104(5):1488–1503. doi:10.1111/1365-2745.12594
MA096	Jessop TS, Lane ML, Teasdale L, Stuart-Fox D, Wilson RS, Careau V, et al. Multiscale Evaluation of Thermal Dependence in the Glucocorticoid Response of Vertebrates. The American Naturalist. 2016;188(3):342–356. doi:10.1086/687588
MA097	Katz DSW. The effects of invertebrate herbivores on plant population growth: a meta-regression analysis. Oecologia. 2016;182(1):43–53. doi:10.1007/s00442-016-3602-9
MA098	Fuzessy LF, Cornelissen TG, Janson C, Silveira FAO. How do primates affect seed germination? A meta-analysis of gut passage effects on neotropical plants. Oikos. 2016;125(8):1069–1080. doi:10.1111/oik.02986
MA099	Cooke J, Leishman MR. Consistent alleviation of abiotic stress with silicon addition: a meta-analysis. Functional Ecology. 2016;30(8):1340–1357. doi:10.1111/1365-2435.12713

ID	Study
MA100	Yoon S, Read Q. Consequences of exotic host use: impacts on Lepidoptera and a test of the ecological trap hypothesis. Oecologia. 2016;181(4):985–996. doi:10.1007/s00442-016-3560-2
MA101	Coetzee BWT, Chown SL. A meta-analysis of human disturbance impacts on Antarctic wildlife. Biological Reviews. 2016;91(3):578–596. doi:10.1111/brv.12184
MA102	Östman Ö, Eklöf J, Eriksson BK, Olsson J, Moksnes PO, Bergström U. Top-down control as important as nutrient enrichment for eutrophication effects in North Atlantic coastal ecosystems. Journal of Applied Ecology. 2016;53(4):1138–1147. doi:10.1111/1365-2664.12654
MA103	He Q, Silliman BR. Consumer control as a common driver of coastal vegetation worldwide. Ecological Monographs. 2016;86(3):278–294. doi:10.1002/ecm.1221
MA106	Delgado-Baquerizo M, Maestre FT, Reich PB, Trivedi P, Osanai Y, Liu YR, et al. Carbon content and climate variability drive global soil bacterial diversity patterns. Ecological Monographs. 2016;86(3):373–390. doi:10.1002/ecm.1216
MA107	Thom D, Seidl R. Natural disturbance impacts on ecosystem services and biodiversity in temperate and boreal forests. Biological Reviews. 2016;91(3):760–781. doi:10.1111/brv.12193
MA108	Liu J, Wu N, Wang H, Sun J, Peng B, Jiang P, et al. Nitrogen addition affects chemical compositions of plant tissues, litter and soil organic matter. Ecology. 2016;97(7):1796–1806. doi:10.1890/15-1683.1
MA109	Liao H, D'Antonio CM, Chen B, Huang Q, Peng S. How much do phenotypic plasticity and local genetic variation contribute to phenotypic divergences along environmental gradients in widespread invasive plants? A meta-analysis. Oikos. 2016;125(7):905–917. doi:10.1111/oik.02372
MA110	Arceo-Gómez G, Ashman TL. Invasion status and phylogenetic relatedness predict cost of heterospecific pollen receipt: implications for native biodiversity decline. Journal of Ecology. 2016;104(4):1003–1008. doi:10.1111/1365-2745.12586
MA111	Oduor AMO, Leimu R, Kleunen Mv. Invasive plant species are locally adapted just as frequently and at least as strongly as native plant species. Journal of Ecology. 2016;104(4):957–968. doi:10.1111/1365-2745.12578
MA112	Ficetola GF, Maiorano L. Contrasting effects of temperature and precipitation change on amphibian phenology, abundance and performance. Oecologia. 2016;181(3):683–693. doi:10.1007/s00442-016-3610-9
MA115	Kriengwatana B, Spierings MJ, ten Cate C. Auditory discrimination learning in zebra finches: effects of sex, early life conditions and stimulus characteristics. Animal Behaviour. 2016;116:99–112. doi:10.1016/j.anbehav.2016.03.028
MA117	Samia DSM, Blumstein DT, Stankowich T, Cooper WE. Fifty years of chasing lizards: new insights advance optimal escape theory. Biological Reviews. 2016;91(2):349–366. doi:10.1111/brv.12173
MA118	Smilanich AM, Fincher RM, Dyer LA. Does plant apparency matter? Thirty years of data provide limited support but reveal clear patterns of the effects of plant chemistry on herbivores. New Phytologist. 2016;210(3):1044–1057. doi:10.1111/nph.13875
MA119	Boyle WA, Sandercock BK, Martin K. Patterns and drivers of intraspecific variation in avian life history along elevational gradients: a meta-analysis. Biological Reviews. 2016;91(2):469–482. doi:10.1111/brv.12180

ID	Study
MA120	Blount JD, Vitikainen EIK, Stott I, Cant MA. Oxidative shielding and the cost of reproduction. Biological Reviews. 2016;91(2):483–497. doi:10.1111/brv.12179
MA121	Wesner JS. Contrasting effects of fish predation on benthic versus emerging prey: a meta-analysis. Oecologia. 2016;180(4):1205–1211. doi:10.1007/s00442-015-3539-4
MA122	Moore MP, Riesch R, Martin RA. The predictability and magnitude of life-history divergence to ecological agents of selection: a meta-analysis in livebearing fishes. Ecology Letters. 2016;19(4):435–442. doi:10.1111/ele.12576
MA123	Rowen E, Kaplan I. Eco-evolutionary factors drive induced plant volatiles: a meta-analysis. New Phytologist. 2016;210(1):284–294. doi:10.1111/nph.13804
MA124	Thurman TJ, Barrett RDH. The genetic consequences of selection in natural populations. Molecular Ecology. 2016;25(7):1429–1448. doi:10.1111/mec.13559
MA125	Hébert MP, Beisner BE, Maranger R. A meta-analysis of zooplankton functional traits influencing ecosystem function. Ecology. 2016;97(4):1069–1080. doi:10.1890/15-1084.1
MA126	Anderson JT. Plant fitness in a rapidly changing world. New Phytologist. 2016;210(1):81–87. doi:10.1111/nph.13693
MA127	Lorenz S, Martinez-Fernández V, Alonso C, Mosselman E, García de Jalón D, González del Tánago M, et al. Fuzzy cognitive mapping for predicting hydromorphological responses to multiple pressures in rivers. Journal of Applied Ecology. 2016;53(2):559–566. doi:10.1111/1365-2664.12569
MA128	Dougherty LR, Shuker DM. Variation in pre- and post-copulatory sexual selection on male genital size in two species of lygaeid bug. Behavioral Ecology and Sociobiology. 2016;70(4):625–637. doi:10.1007/s00265-016-2082-6
MA129	Crouzeilles R, Curran M. Which landscape size best predicts the influence of forest cover on restoration success? A global meta-analysis on the scale of effect. Journal of Applied Ecology. 2016;53(2):440–448. doi:10.1111/1365-2664.12590
MA130	van Katwijk MM, Thorhaug A, Marbà N, Orth RJ, Duarte CM, Kendrick GA, et al. Global analysis of seagrass restoration: the importance of large-scale planting. Journal of Applied Ecology. 2016;53(2):567–578. doi:10.1111/1365-2664.12562
MA133	McCary MA, Mores R, Farfan MA, Wise DH. Invasive plants have different effects on trophic structure of green and brown food webs in terrestrial ecosystems: a meta-analysis. Ecology Letters. 2016;19(3):328–335. doi:10.1111/ele.12562
MA135	Mazé-Guilmo E, Blanchet S, McCoy KD, Loot G. Host dispersal as the driver of parasite genetic structure: a paradigm lost? Ecology Letters. 2016;19(3):336–347. doi:10.1111/ele.12564
MA136	Allen DC, Wesner JS. Synthesis: comparing effects of resource and consumer fluxes into recipient food webs using meta-analysis. Ecology. 2016;97(3):594–604. doi:10.1890/15-1109.1
MA137	Zvereva EL, Kozlov MV. The costs and effectiveness of chemical defenses in herbivorous insects: a meta-analysis. Ecological Monographs. 2016;86(1):107–124. doi:10.1890/15-0911.1

ID	Study
MA138	Barton KE. Tougher and thornier: general patterns in the induction of physical defence traits. Functional Ecology. 2016;30(2):181–187. doi:10.1111/1365-2435.12495
MA140	Street SE, Cross CP, Brown GR. Exaggerated sexual swellings in female nonhuman primates are reliable signals of female fertility and body condition. Animal Behaviour. 2016;112(Supplement C):203–212. doi:10.1016/j.anbehav.2015.11.023
MA145	Moore FR, Shuker DM, Dougherty L. Stress and sexual signaling: a systematic review and meta-analysis. Behavioral Ecology. 2016;27(2):363–371. doi:10.1093/beheco/arv195
MA146	Roca IT, Desrochers L, Giacomazzo M, Bertolo A, Bolduc P, Deschesnes R, et al. Shifting song frequencies in response to anthropogenic noise: a meta-analysis on birds and anurans. Behavioral Ecology. 2016;27(5):1269–1274. doi:10.1093/beheco/arw060
MA147	Holman L. Bet hedging via multiple mating: A meta-analysis. Evolution. 2016;70(1):62–71. doi:10.1111/evo.12822
MA148	Vico G, Manzoni S, Nkurunziza L, Murphy K, Weih M. Trade-offs between seed output and life span – a quantitative comparison of traits between annual and perennial congeneric species. New Phytologist. 2016;209(1):104–114. doi:10.1111/nph.13574
MA149	Daskin JH, Pringle RM. Does primary productivity modulate the indirect effects of large herbivores? A global meta-analysis. Journal of Animal Ecology. 2016;85(4):857–868. doi:10.1111/1365-2656.12522
MA150	Gunton RM, Pöyry J. Scale-specific spatial density dependence in parasitoids: a multi-factor meta-analysis. Functional Ecology. 2016;30(9):1501–1510. doi:10.1111/1365-2435.12627
MA151	German RN, Thompson CE, Benton TG. Relationships among multiple aspects of agriculture's environmental impact and productivity: a meta-analysis to guide sustainable agriculture. Biological Reviews. 2017;92(2):716–738. doi:10.1111/brv.12251
MA152	Shantz AA, Lemoine NP, Burkepile DE. Nutrient loading alters the performance of key nutrient exchange mutualisms. Ecology Letters. 2016;19(1):20–28. doi:10.1111/ele.12538
MA153	Dillingham PW, Moore JE, Fletcher D, Cortés E, Curtis KA, James KC, et al. Improved estimation of intrinsic growth rmax for long-lived species: integrating matrix models and allometry. Ecological Applications. 2016;26(1):322–333. doi:10.1890/14-1990
MA155	Strader ME, Aglyamova GV, Matz MV. Red fluorescence in coral larvae is associated with a diapause-like state. Molecular Ecology. 2016;25(2):559–569. doi:10.1111/mec.13488
MA157	Wood KA, O'Hare MT, McDonald C, Searle KR, Daunt F, Stillman RA. Herbivore regulation of plant abundance in aquatic ecosystems. Biological Reviews. 2017;92(2):1128–1141. doi:10.1111/brv.12272
MA158	Deng Q, Hui D, Luo Y, Elser J, Wang YP, Loladze I, et al. Down-regulation of tissue N:P ratios in terrestrial plants by elevated CO2. Ecology. 2015;96(12):3354–3362. doi:10.1890/15-0217.1
MA159	Garamszegi LZ, Markó G, Szász E, Zsebők S, Azcárate M, Herczeg G, et al. Among-year variation in the repeatability, within- and between-individual, and phenotypic correlations of behaviors in a natural population. Behavioral Ecology and Sociobiology. 2015;69(12):2005–2017. doi:10.1007/s00265-015-2012-z

ID	Study
MA160	Lüpold S, Simmons LW, Tomkins JL, Fitzpatrick JL. No evidence for a trade-off between sperm length and male premating weaponry. Journal of Evolutionary Biology. 2015;28(12):2187–2195. doi:10.1111/jeb.12742
MA162	Moreno-Mateos D, Meli P, Vara-Rodríguez MI, Aronson J. Ecosystem response to interventions: lessons from restored and created wetland ecosystems. Journal of Applied Ecology. 2015;52(6):1528–1537. doi:10.1111/1365-2664.12518
MA163	Katano I, Doi H, Eriksson BK, Hillebrand H. A cross-system meta-analysis reveals coupled predation effects on prey biomass and diversity. Oikos. 2015;124(11):1427–1435. doi:10.1111/oik.02430
MA164	Graham S, Chapuis E, Meconcelli S, Bonel N, Sartori K, Christophe A, et al. Size-assortative mating in simultaneous hermaphrodites: an experimental test and a meta-analysis. Behavioral Ecology and Sociobiology. 2015;69(11):1867–1878. doi:10.1007/s00265-015-1999-5
MA168	Goessling JM, Kennedy H, Mendonça MT, Wilson AE. A meta-analysis of plasma corticosterone and heterophil : lymphocyte ratios – is there conservation of physiological stress responses over time? Functional Ecology. 2015;29(9):1189–1196. doi:10.1111/1365-2435.12442
MA169	Romero GQ, Gonçalves-Souza T, Vieira C, Koricheva J. Ecosystem engineering effects on species diversity across ecosystems: a meta-analysis. Biological Reviews. 2015;90(3):877–890. doi:10.1111/brv.12138
MA170	Nielsen JM, Popp BN, Winder M. Meta-analysis of amino acid stable nitrogen isotope ratios for estimating trophic position in marine organisms. Oecologia. 2015;178(3):631–642. doi:10.1007/s00442-015-3305-7
MA171	James J, Slater FM, Vaughan IP, Young KA, Cable J. Comparing the ecological impacts of native and invasive crayfish: could native species' translocation do more harm than good? Oecologia. 2015;178(1):309–316. doi:10.1007/s00442-014-3195-0
MA176	Esteban R, Barrutia O, Artetxe U, Fernández-Marín B, Hernández A, García-Plazaola JI. Internal and external factors affecting photosynthetic pigment composition in plants: a meta-analytical approach. New Phytologist. 2015;206(1):268–280. doi:10.1111/nph.13186
MA178	Becker DJ, Streicker DG, Altizer S. Linking anthropogenic resources to wildlife–pathogen dynamics: a review and meta-analysis. Ecology Letters. 2015;18(5):483–495. doi:10.1111/ele.12428
MA179	Starko S, Claman BZ, Martone PT. Biomechanical consequences of branching in flexible wave-swept macroalgae. New Phytologist. 2015;206(1):133–140. doi:10.1111/nph.13182
MA180	Hsu YH, Schroeder J, Winney I, Burke T, Nakagawa S. Are extra-pair males different from cuckolded males? A case study and a meta-analytic examination. Molecular Ecology. 2015;24(7):1558–1571. doi:10.1111/mec.13124
MA181	Mazza CA, Ballaré CL. Photoreceptors UVR8 and phytochrome B cooperate to optimize plant growth and defense in patchy canopies. New Phytologist. 2015;207(1):4–9. doi:10.1111/nph.13332
MA182	Jackson MC. Interactions among multiple invasive animals. Ecology. 2015;96(8):2035–2041. doi:10.1890/15-0171.1
MA183	Arct A, Drobniak SM, Cichoń M. Genetic similarity between mates predicts extrapair paternity—a meta-analysis of bird studies. Behavioral Ecology. 2015;26(4):959–968. doi:10.1093/beheco/arv004

ID	Study
MA184	Boudreau SA, Anderson SC, Worm B. Top-down and bottom-up forces interact at thermal range extremes on American lobster. Journal of Animal Ecology. 2015;84(3):840–850. doi:10.1111/1365-2656.12322
MA185	Yang LY, Machado CA, Dang XD, Peng YQ, Yang DR, Zhang DY, et al. The incidence and pattern of copollinator diversification in dioecious and monoecious figs. Evolution. 2015;69(2):294–304. doi:10.1111/evo.12584
MA186	Siefert A, Violle C, Chalmandrier L, Albert CH, Taudiere A, Fajardo A, et al. A global meta-analysis of the relative extent of intraspecific trait variation in plant communities. Ecology Letters. 2015;18(12):1406–1419. doi:10.1111/ele.12508
MA187	Beninde J, Veith M, Hochkirch A. Biodiversity in cities needs space: a meta-analysis of factors determining intra-urban biodiversity variation. Ecology Letters. 2015;18(6):581–592. doi:10.1111/ele.12427
MA188	Senior AM, Nakagawa S, Lihoreau M, Simpson SJ, Raubenheimer D. An Overlooked Consequence of Dietary Mixing: A Varied Diet Reduces Interindividual Variance in Fitness. The American Naturalist. 2015;186(5):649–659. doi:10.1086/683182
MA189	Albertson LK, Allen DC. Meta-analysis: abundance, behavior, and hydraulic energy shape biotic effects on sediment transport in streams. Ecology. 2015;96(5):1329–1339. doi:10.1890/13-2138.1
MA190	Frankham R. Genetic rescue of small inbred populations: meta-analysis reveals large and consistent benefits of gene flow. Molecular Ecology. 2015;24(11):2610–2618. doi:10.1111/mec.13139
MA191	Voje KL. Scaling of Morphological Characters across Trait Type, Sex, and Environment: A Meta-analysis of Static Allometries. The American Naturalist. 2015;187(1):89–98. doi:10.1086/684159
MA192	Slot M, Kitajima K. General patterns of acclimation of leaf respiration to elevated temperatures across biomes and plant types. Oecologia. 2015;177(3):885–900. doi:10.1007/s00442-014-3159-4
MA193	Jahnke M, Olsen JL, Procaccini G. A meta-analysis reveals a positive correlation between genetic diversity metrics and environmental status in the long-lived seagrass Posidonia oceanica. Molecular Ecology. 2015;24(10):2336–2348. doi:10.1111/mec.13174
MA194	Iacarella JC, Dick JTA, Alexander ME, Ricciardi A. Ecological impacts of invasive alien species along temperature gradients: testing the role of environmental matching. Ecological Applications. 2015;25(3):706–716. doi:10.1890/14-0545.1
MA195	Quesnelle PE, Lindsay KE, Fahrig L. Relative effects of landscape-scale wetland amount and landscape matrix quality on wetland vertebrates: a meta-analysis. Ecological Applications. 2015;25(3):812–825. doi:10.1890/14-0362.1
MA197	Dougherty LR, Shuker DM. The effect of experimental design on the measurement of mate choice: a meta-analysis. Behavioral Ecology. 2015;26(2):311–319. doi:10.1093/beheco/aru125
MA198	Paz-Vinas I, Loot G, Stevens VM, Blanchet S. Evolutionary processes driving spatial patterns of intraspecific genetic diversity in river ecosystems. Molecular Ecology. 2015;24(18):4586–4604. doi:10.1111/mec.13345

ID	Study
MA199	Sistla SA, Appling AP, Lewandowska AM, Taylor BN, Wolf AA. Stoichiometric flexibility in response to fertilization along gradients of environmental and organismal nutrient richness. Oikos. 2015;124(7):949–959. doi:10.1111/oik.02385
MA200	Jauni M, Gripenberg S, Ramula S. Non-native plant species benefit from disturbance: a meta-analysis. Oikos. 2015;124(2):122–129. doi:10.1111/oik.01416
MA201	Bunn RA, Ramsey PW, Lekberg Y. Do native and invasive plants differ in their interactions with arbuscular mycorrhizal fungi? A meta-analysis. Journal of Ecology. 2015;103(6):1547–1556. doi:10.1111/1365-2745.12456
MA202	Mehrabi Z, Tuck SL. Relatedness is a poor predictor of negative plant–soil feedbacks. New Phytologist. 2015;205(3):1071–1075. doi:10.1111/nph.13238
MA203	Wang X, Taub DR, Jablonski LM. Reproductive allocation in plants as affected by elevated carbon dioxide and other environmental changes: a synthesis using meta-analysis and graphical vector analysis. Oecologia. 2015;177(4):1075–1087. doi:10.1007/s00442-014-3191-4
MA204	Albert A, Auffret AG, Cosyns E, Cousins SAO, D'hondt B, Eichberg C, et al. Seed dispersal by ungulates as an ecological filter: a trait-based meta-analysis. Oikos. 2015;124(9):1109–1120. doi:10.1111/oik.02512
MA205	Mijangos JL, Pacioni C, Spencer PBS, Craig MD. Contribution of genetics to ecological restoration. Molecular Ecology. 2015;24(1):22–37. doi:10.1111/mec.12995
MA206	Tamburello L, Maggi E, Benedetti-Cecchi L, Bellistri G, Rattray AJ, Ravaglioli C, et al. Variation in the impact of non-native seaweeds along gradients of habitat degradation: a meta-analysis and an experimental test. Oikos. 2015;124(9):1121–1131. doi:10.1111/oik.02197
MA207	Ferreira V, Castagneyrol B, Koricheva J, Gulis V, Chauvet E, Graça MAS. A meta-analysis of the effects of nutrient enrichment on litter decomposition in streams. Biological Reviews. 2015;90(3):669–688. doi:10.1111/brv.12125
MA208	Stephens AEA, Westoby M. Effects of insect attack to stems on plant survival, growth, reproduction and photosynthesis. Oikos. 2015;124(3):266–273. doi:10.1111/oik.01809
MA210	Anderegg WRL. Spatial and temporal variation in plant hydraulic traits and their relevance for climate change impacts on vegetation. New Phytologist. 2015;205(3):1008–1014. doi:10.1111/nph.12907
MA211	Yuan ZY, Chen HYH. Negative effects of fertilization on plant nutrient resorption. Ecology. 2015;96(2):373–380. doi:10.1890/14-0140.1
MA212	Valls A, Coll M, Christensen V. Keystone species: toward an operational concept for marine biodiversity conservation. Ecological Monographs. 2015;85(1):29–47. doi:10.1890/14-0306.1
MA213	Colautti RI, Lau JA. Contemporary evolution during invasion: evidence for differentiation, natural selection, and local adaptation. Molecular Ecology. 2015;24(9):1999–2017. doi:10.1111/mec.13162
MA214	Fuiman LA, Connelly TL, Lowerre-Barbieri SK, McClelland JW. Egg boons: central components of marine fatty acid food webs. Ecology. 2015;96(2):362–372. doi:10.1890/14-0571.1

ID	Study
MA215	Baeten L, Davies TJ, Verheyen K, Calster HV, Vellend M. Disentangling dispersal from phylogeny in the colonization capacity of forest understorey plants. Journal of Ecology. 2015;103(1):175–183. doi:10.1111/1365-2745.12333
MA217	Taranu ZE, Gregory-Eaves I, Leavitt PR, Bunting L, Buchaca T, Catalan J, et al. Acceleration of cyanobacterial dominance in north temperate-subarctic lakes during the Anthropocene. Ecology Letters. 2015;18(4):375–384. doi:10.1111/ele.12420
MA218	Pintor LM, Byers JE. Do native predators benefit from non-native prey? Ecology Letters. 2015;18(11):1174–1180. doi:10.1111/ele.12496
MA220	Harper KA, Macdonald SE, Mayerhofer MS, Biswas SR, Esseen PA, Hylander K, et al. Edge influence on vegetation at natural and anthropogenic edges of boreal forests in Canada and Fennoscandia. Journal of Ecology. 2015;103(3):550–562. doi:10.1111/1365-2745.12398
MA222	Culina A, Radersma R, Sheldon BC. Trading up: the fitness consequences of divorce in monogamous birds. Biological Reviews. 2015;90(4):1015–1034. doi:10.1111/brv.12143
MA223	Bracken MES, Hillebrand H, Borer ET, Seabloom EW, Cebrian J, Cleland EE, et al. Signatures of nutrient limitation and co-limitation: responses of autotroph internal nutrient concentrations to nitrogen and phosphorus additions. Oikos. 2015;124(2):113–121. doi:10.1111/oik.01215
MA224	Périquet S, Fritz H, Revilla E. The Lion King and the Hyaena Queen: large carnivore interactions and coexistence. Biological Reviews. 2015;90(4):1197–1214. doi:10.1111/brv.12152
MA226	Elliott KH, Hare JF, Vaillant ML, Gaston AJ, Ropert-Coudert Y, Anderson WG. Ageing gracefully: physiology but not behaviour declines with age in a diving seabird. Functional Ecology. 2015;29(2):219–228. doi:10.1111/1365-2435.12316
MA227	Lafuente A, Pérez-Palacios P, Doukkali B, Molina-Sánchez MD, Jiménez-Zurdo JI, Caviedes MA, et al. Unraveling the effect of arsenic on the model Medicago–Ensifer interaction: a transcriptomic meta-analysis. New Phytologist. 2015;205(1):255–272. doi:10.1111/nph.13009
MA229	Gamfeldt L, Lefcheck JS, Byrnes JEK, Cardinale BJ, Duffy JE, Griffin JN. Marine biodiversity and ecosystem functioning: what's known and what's next? Oikos. 2015;124(3):252–265. doi:10.1111/oik.01549

3 REVIEW OF JOURNAL POLICIES ON DATA AND CODE SHARING

The availability of data and code for individual articles needs to be evaluated in the context of the publishing journals' policies about making data and code available at the time of publication. Due to the retrospective nature of this study, information about the journals' data and code policies contemporaneous with the articles published 2015–17 was not available. The journals' policies on data and code were inferred from other sources, including previous studies of journal policies and initiatives such as the Joint Data Archiving Policy (JDAP).

Data policies

JDAP was adopted by a number of journals in the fields of ecology and evolutionary biology in 2011 (195). JDAP introduced data archiving as a requirement for publication: the data that underlie the results of the article must be deposited in a public data repository, such as the Dryad Digital Repository (196). Four of the journals in this study adopted JDAP in 2011: *Evolution* (197), *Journal of Evolutionary Biology* (198), *Molecular Ecology* (199), and *The American Naturalist* (200). *Functional Ecology* adopted a slight variation of JDAP in 2014 (201), along with other journals published by the British Ecological Society: *Journal of Animal Ecology*, *Journal of Applied Ecology*, and *Journal of Ecology* (202). Therefore, assuming the journals' adoption of JDAP (or slight variation thereof) has persisted, we expected that these eight journals would have mandated data archiving for all studies published through 2015–17.

(17) investigated both the data and code policies for 17 of the 21 journal titles in this study. The policies checked were as of 1st June 2015, which is within the 2015–17 time period. Specifically, regarding data, (17) recorded whether journals' policies *required* data to be released as a condition of publication—that is, beyond mere encouragement to make data available. We regarded the findings in (17) as representing journals' policies on data and code at the start of 2015 (it is possible that some meta-analyses published in the first five months of 2015 were published under a different journal policy that then changed to the policy found by (17), but for simplicity we discounted this possibility). The four journals that were not reviewed in (17) are *Animal Behaviour*, *Biological Reviews*, *New Phytologist*, and *Quarterly Review of Biology*. We examined other sources to get an indication of their data and code policies.

The data policy of the journal *Animal Behaviour* was surveyed in January 2014 by (203). This survey found that the journal encouraged authors to make data available, but did not make it mandatory. In the absence of other information (which was searched for in e.g., editorials or news releases, but not found), we assume that this was the data policy of the journal during 2015–17. (When checked again in 2021, the journal was found to have the same policy of encouraging data sharing, so it seems safe to assume the policy has been consistently in place since 2014.) When checked in 2021, the journal *Biological Reviews* "encourages" authors to make data available, but does not require authors to do so, or to include data availability statements (204). In the absence of other information, we assume that this was the data policy of the journal during 2015–17. The data policy of the journal *New Phytologist* was surveyed in August/September 2013 by (205). This survey classified the policy of *New Phytologist* as *weak*, meaning that data sharing was encouraged but not required. In the absence of other information, we assume that this was the data policy of the journal during 2015–17. When checked in 2021, the instructions to authors webpage for the journal *Quarterly Review of*

Biology[1] makes no mention of data sharing, archiving, or availability. Similarly, an archived snapshot of the instructions to authors webpage as it was on 28th May 2016[2] made no mention of any data policy. In the absence of other information, we assume that not requiring data sharing was the effective data policy of the journal during 2015–17. A summary of the data-sharing policies of the journals in this study is given in Table 3.

Journal	JDAP member	Data sharing required?	Source
Animal Behaviour	N	N	(203)
Behavioral Ecology	N	N	(17)
Behavioral Ecology and Sociobiology	N	N	(17)
Biological Reviews	N	N	Journal website
Ecological Applications	N	Y	(17)
Ecological Monographs	N	Y	(17)
Ecology	N	Y	(17)
Ecology Letters	N	Y	(17)
Evolution	Y	Y	(17)
Evolutionary Ecology	N	N	(17)
Functional Ecology	Y	Y	(17)
Journal of Animal Ecology	Y	Y	(17)
Journal of Applied Ecology	Y	Y	(17)
Journal of Ecology	Y	Y	(17)
Journal of Evolutionary Biology	Y	Y	(17)
Molecular Ecology	Y	Y	(17)
New Phytologist	N	N	(205)
Oecologia	N	N	(17)
Oikos	N	Y	(17)
The American Naturalist	Y	Y	(17)
The Quarterly Review of Biology	N	N	Journal website

Table 3: Summary of whether data sharing was found to be required for each journal surveyed in this study, along with JDAP member status and source of the information. In the columns "JDAP member" and "Data sharing required?", "Y" indicates "yes" and "N" indicates "no".

Code policies

The principal source for information about journals' code policies came from (17). (206) updated the information about journals' code policies in 2020. Both studies recorded information about the code policies of 17 of the journals included in this present study. (17) recorded whether journals required the release of code as a requirement for publication as a binary yes/no variable (the same way as how journals' data policies were recorded). The updated survey in (206) distinguished between policies where code sharing was "encour-

[1] https://www.journals.uchicago.edu/journals/qrb/instruct

[2] https://web.archive.org/web/20160528051141/http://www.journals.uchicago.edu/journals/qrb/instruct

aged" and policies where code sharing was "mandatory" (the authors note that some journal policies were ambiguously worded such that it could not be determined whether code sharing was merely encouraged or a mandatory requirement; they designated such policies "encouraged/mandatory"). For the four journals not covered in (17), we found that *Animal Behaviour* had a policy of encouraging code sharing, but we could not find mention of polic(ies) about code in the online information for *Biological Reviews*, *New Phytologist*, and *The Quarterly Review of Biology*. For the purposes of this study, we shall regard these four journals as not having had a policy requiring code sharing during 2015–17.

Journal	2015 survey	2020 survey	2021 check
Animal Behaviour	-	-	E
Behavioral Ecology	N	N	-
Behavioral Ecology and Sociobiology	N	N	-
Biological Reviews	-	-	N.F.
Ecological Applications	Y	M	-
Ecological Monographs	Y	M	-
Ecology	Y	M	-
Ecology Letters	N	E/M	-
Evolution	N	M	-
Evolutionary Ecology	N	E	-
Functional Ecology	Y	E/M	-
Journal of Animal Ecology	Y	E/M	-
Journal of Applied Ecology	Y	E/M	-
Journal of Ecology	Y	E/M	-
Journal of Evolutionary Biology	N	M	-
Molecular Ecology	Y	E	-
New Phytologist	-	-	N.F.
Oecologia	N	N	-
Oikos	N	N	-
The American Naturalist	Y	E	-
The Quarterly Review of Biology	-	-	N.F.

Table 4: Summary of the code sharing policies found for each journal. The column "2015 survey" refers to (17), the column "2020 survey" refers to (206), and the column "2021 check" refers to our own checks made in 2021. Within the table columns, "Y" indicates "yes", "N" indicates "no", "E" indicates "encouraged", "M" indicates "mandatory", and "N.F." indicates "not found".

4 CODING SCHEME FOR CODE AND DATA SHARING

The assessment process for each article for shared data and code was as follows: first, we inspected the end sections of each article for any mention of supplemental material, and for the existence of a data/code availability statement of any kind. In cases without an explicit data availability statement, or where data/code were not listed as supplements, we reviewed the methods and results sections for any possible in-text mention of data/code availability, first by performing a keyword search for "data". Regardless of what was mentioned in the article, we also inspected the journal webpage for each article (accessed via The University of Melbourne

library) for indications and details of supplemental materials, shared data and shared code. We attempted to download and briefly inspect all files at the journal webpage that we found. Where supplemental material, data and/or code were reported as existing at other web links (e.g., an online data archive), we followed the web links and attempted to download and inspect all files we found. The coding scheme in Table 5 captures the results of this process.

This coding scheme assumes that if data and/or code were shared, there would be some positive indication of this fact somewhere in the article itself, or on the journal publisher's web page for the article (either as supplemental material, or as a link to an independent resource). An absence of any such indication was taken to mean that data/code was not shared. This approach does not account for the possibility that authors may have in fact shared the data and code associated with their article (say, by publishing it in a data repository such as Dryad) but not included any indication either in the article itself, or on the journal web page for the article. (One possible reason this might occur is when authors decide to share the data/code after the article had been published.) We decided not to attempt to check for such possibilities when assessment of an article and its journal web page found no indications of shared data or code.

In this coding scheme, items 1–4 concern supplemental material in general, items 5–12 concern shared data in particular, and items 13–20 concern shared code in particular (item 21 was used to record any additional notes). The items recording the existence (or not) of shared supplemental materials, data, and code are items 1, 5–6, and 13–14. For the data and code sharing, we separated out the nominal sharing of these from the actual sharing of these (in retrospect, we should have done the same for supplemental materials too). In this context, data and code were recorded as having been actually shared only if we were personally able to successfully download (via The University of Melbourne library) and inspect the relevant file(s).

The numbers of files shared (items 2, 7, and 15) were recorded to help keep track of downloaded files. These values were recorded only if the respective preceding items indicated that such files existed. This did lead to some anomalies: the number of files was recorded as 0 in cases where the article stated that all relevant data was made available within tables of the article itself (and not as a separate data file).

5 RECORDING MENTIONS OF SOFTWARE USED

The review process for mentions of software in an article consisted of checking the text of each article/supplementary document for the following keywords (using a case-insensitive search):

- "CMA", referring to the software package *Comprehensive Meta-Analysis* (207);
- "MetaWin", referring to the software package *MetaWin* (208);

Index	Field	Values	Description
1	Supplements included	Y, N	Does the article include supplementary information?
2	No. supplement files	0-99	Number of discrete files or documents included as supplementary information
3	Supplements mentioned	Y, N	Does the article *mention* the existence of the supplementary information?
4	Supplements detailed	Y, N	Does the article provide details of the contents of supplementary information?
5	Datasets nominally included	Y, N	Does the article *indicate* that data has been shared, included?
6	Datasets included	Y, N	Was the data actually included (shared) and obtainable?
7	No. data files	0-99	Number of discrete data files included
8	Dataset sources	open text	Location of the datasets (e.g., repository name)
9	Dataset URL	open text	Link to data as applicable
10	Dataset info in article	Y, N	Is the availability of data referred to in the article?
11	Dataset info on website	Y, N	Is the availability of data referred to on the journal web page for the article?
12	Data format	open text	File format(s) of data files
13	Code nominally included	Y, N	Does the article *indicate* that code has been shared, included?
14	Code included	Y, N	Was the code actually included (shared) and obtainable?
15	No. code files	0-99	Number of discrete code files included
16	Code sources	open text	Location of the code (e.g., repository name)
17	Code URL	open text	Link to code as applicable
18	Code info in article	Y, N	Is the availability of code referred to in the article?
19	Code info on website	Y, N	Is the availability of code referred to on the journal web page for the article?
20	Code type	open text	Language or software package the code is associated with
21	Notes	open text	Any additional notes about the article's data and code sharing.

Table 5: The twenty-one item coding scheme used for recording data and code sharing in meta-analysis articles. In the Values column, "Y" indicates "yes" and "N" indicates "no".

- "metafor", referring to the R package *metafor* (209);
- "mcmcglmm", referring to the R package *mcmcglmm* (210).

In the absence of these keywords being found, the methods section/supplementary document was manually scanned for statements along the lines of "analyses were performed using [software package]".

For each mention of software used (allowing for multiple mentions per article), the details were recorded using a ten-item coding scheme outlined in Table 6. The coding scheme was designed around an expectation of the frequent mention of R and R packages.

Items 1 and 2 record the name of the software package/platform as reported in the article and the page number of the mention respectively.

Items 3 and 4 record whether a specific version of the software was reported. Items 5, 6, and 7 are specific to the R software environment.

Item 5 is a flag indicating whether the mentioned software package was an R package or not. This required judgment beyond what was reported in the article: For most software mentioned, we were able to code this item based on our own knowledge of R and its packages; where we were not already familiar with the software package, we used contextual clues in the article (e.g., mentions of the function of the software or details from the citation if provided) and online searches of the software name to determine whether or not it was an R package.

Item 6 was only applicable to software identified as an R package: this recorded the location where the R package was hosted. We anticipated that there would be few discrete categories here: "base" referring to packages which are part of the base R installation; "Bioconductor" referring to R packages released as components of the Bioconductor project; "CRAN" referring to the Comprehensive R Archive Network, a repository for R packages; and "other" for all remaining cases.

Item 7 is applicable only to mentions of the R software environment at large: this records whether in addition to the mention of R, specific R packages mentioned as well.

Items 8 and 9 record whether and how the article cited/provided a reference for the software mentioned. Item 8 was initially "Y"/"N" (yes/no), during the coding process we decided to introduce an addition code "T" which was for instances of an "in text" reference for the software (e.g., the website for the software package in parentheses immediately following the software name) but with no corresponding details in the "References" section of the article. As a result, a value of "Y" indicates that the article includes a full reference

to the software in the References section. The full reference (or in-text only citation) as reported in the article is recorded in Item 9.

Finally, Item 10 was used to record additional notes/context about the mention of the software as applicable.

Index	Field	Values	Description
1	Software details	open text	The name of the software as reported in the article.
2	Page reference	open text	Specify the page number of the mention.
3	Version specified	Y, N	Does the article specify the version of the software?
4	Version details	open text	The version details as reported in the article.
5	Is R package	Y, N	Is the software mentioned an R package?
6	R package location	base, Bioconductor, CRAN, other, N/A	If the software mentioned is an R package, where is the package located/hosted?
7	R packages mentioned	Y, N, N/A	If the software mentioned is R, are packages mentioned elsewhere in the article?
8	Software cited	Y, N, T	Does the article include a citation for the software package?
9	Citation details	open text	The full reference to the software as reported in the article.
10	Notes	open text	Any additional notes about this mention of software.

Table 6: The ten item coding scheme used for recording software mentions. In the Values column, "Y" indicates "yes", "N" indicates "no", "T" indicates "in-text only", and "N/A" indicates "not applicable".

6 DATA AND CODE SHARING

For the 133 articles with data, we had found some kind of indication about data availability somewhere in the article itself or in the supplementary documentation for all but one article (in this one case, the information indicating that data was available was on the journal's web page for the article instead). This took the form of either an explicit data availability statement in the article, or a mention in the body of the article, as part of an in-article statement about the content of supplemental/supporting information, or in the supplemental/supporting information itself. For example, *Evolution* articles included a data availability statement in a dedicated section titled "Data Archiving" located at the end of the article, just before the references section.

Failures to obtain data and code

We failed to obtain data for five articles for three reasons: for the first three cases, a supplemental document indicated that data files were included as part of the supplemental material. However, the files referred to could not be found as part of the online supplement; it is possible that while the documentation for the data was uploaded, the actual files themselves were not. In the fourth case, the data availability statement said that data would be uploaded to Dryad upon acceptance of the article, however no link or details of how to find the data were provided (failing to update the data availability statement may have been an oversight when the article was being finalised for publication). In the final case, the article stated that data had been deposited in a research institute's database, but failed to provide any details apart from a link to the institute's main web page. The institute maintains a number of databases, and there was no clear way to identify which data in which database was relevant to the meta-analysis.

The one case where we could not obtain code is the one of the articles discussed above in reference to data availability, where files listed as being part of the supplement could not be found.

Data and Code Sharing by Journal

Figure 1 shows the rate of data sharing within each journal title. Figure 2 compares the observed rate of data sharing amongst JPAP journals to that of non-JDAP journals. Figure 3 compares the observed rate of data sharing between all journals requiring data sharing (JDAP or not) against those journals without a data sharing requirement. These figures show that in this sample, journal policies did increase the likelihood that a meta-analysis article would include accessible data, and although clearly a common practice among researchers submitting to these journals, adherence to the data sharing policy was not 100%.

Figure 4 shows the rate of code sharing within each journal title. Figure 5 compares the sharing rates for code across three categories: code required, code not required, and policy not known. This figure re-emphasises

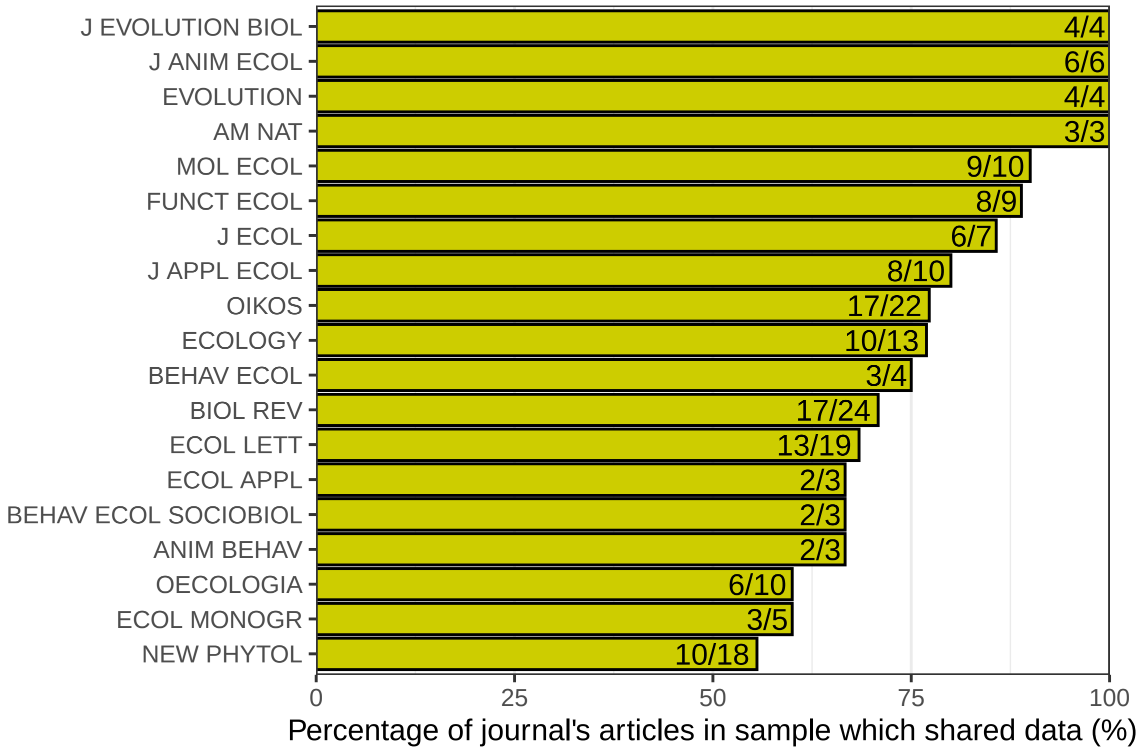

Figure 1: Comparison of data sharing rates in articles by journal.

that code sharing during this period was low, despite some journals' policies requiring code (these journals did have the highest rate of code sharing, but it was still less than 25% of articles published). During this period, code sharing lagged well behind data sharing (which was common practice in comparison).

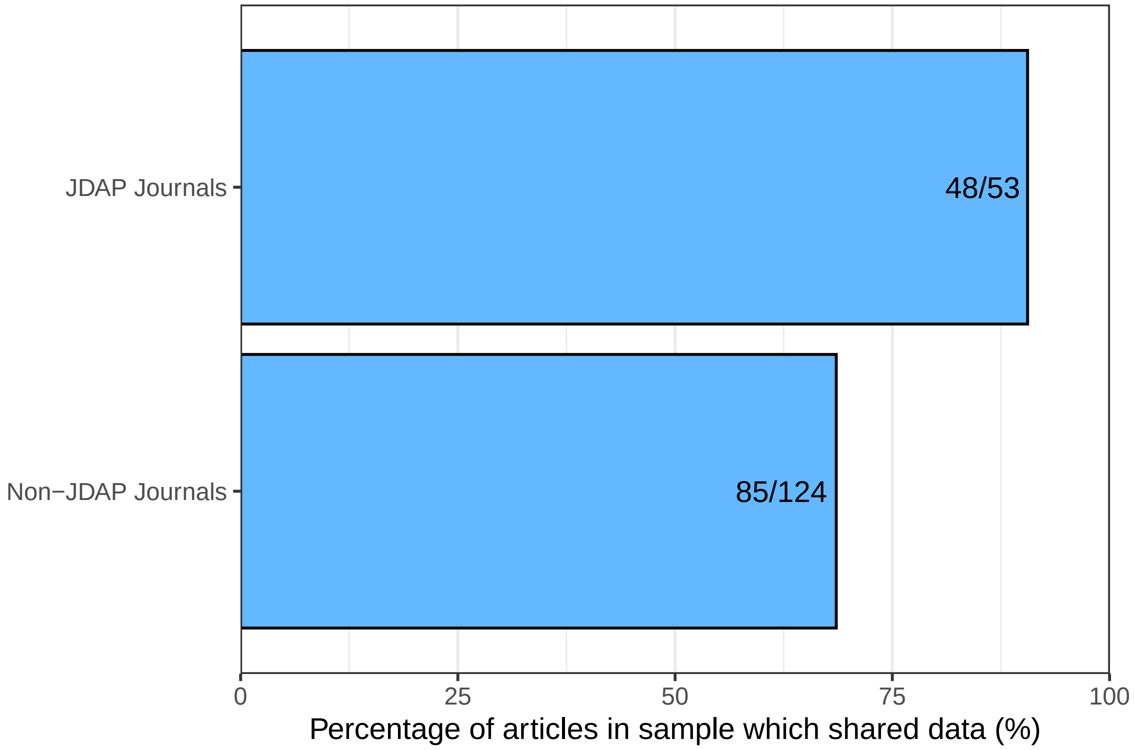

Figure 2: Comparison of data sharing rates in articles by journal JDAP membership status.

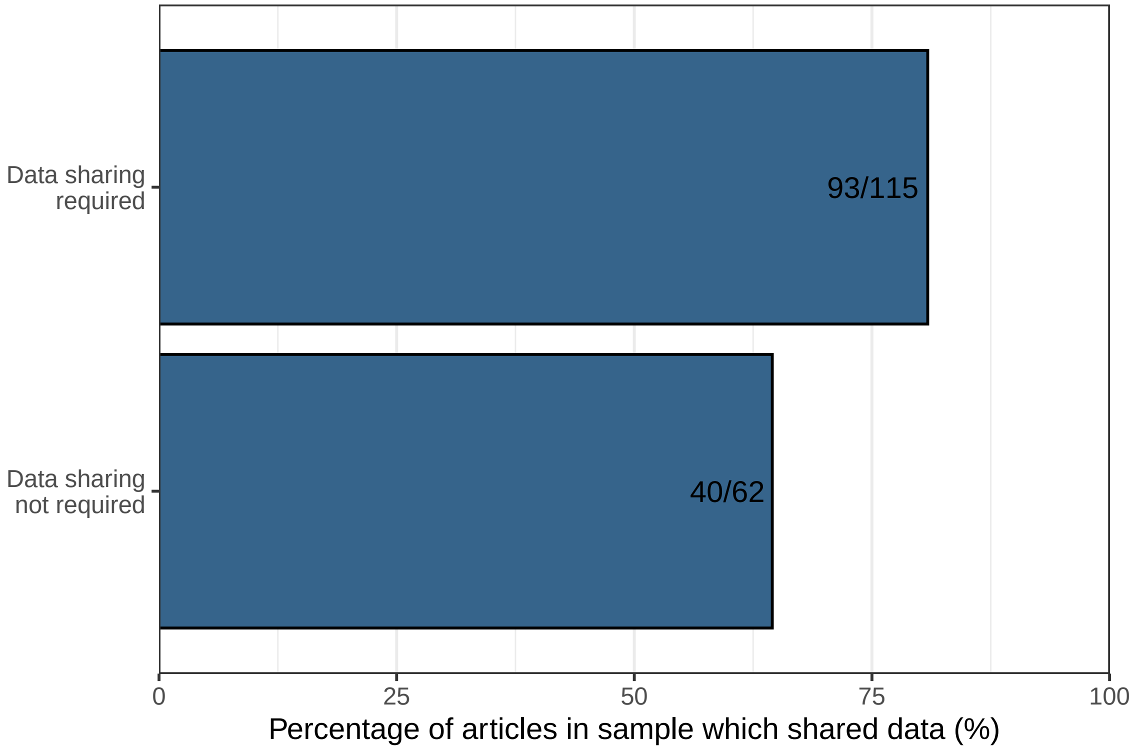

Figure 3: Comparison of data sharing rates in articles by journal data policy 2015–17.

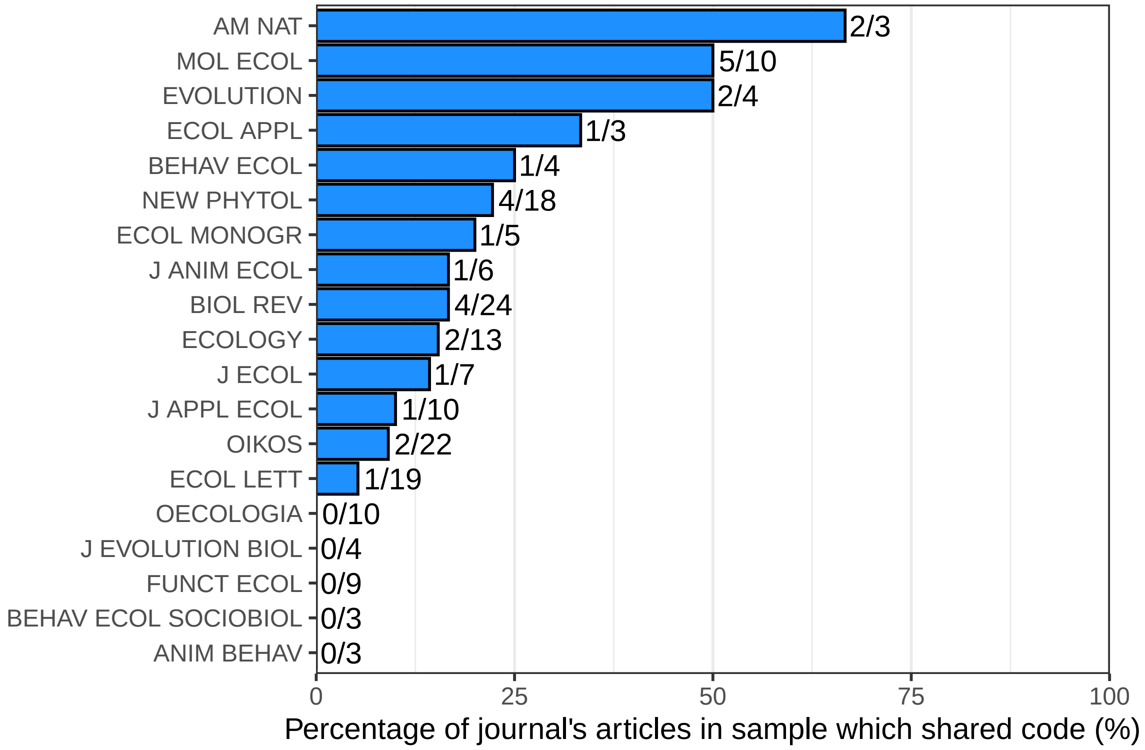

Figure 4: Comparison of code sharing rates in articles by journal.

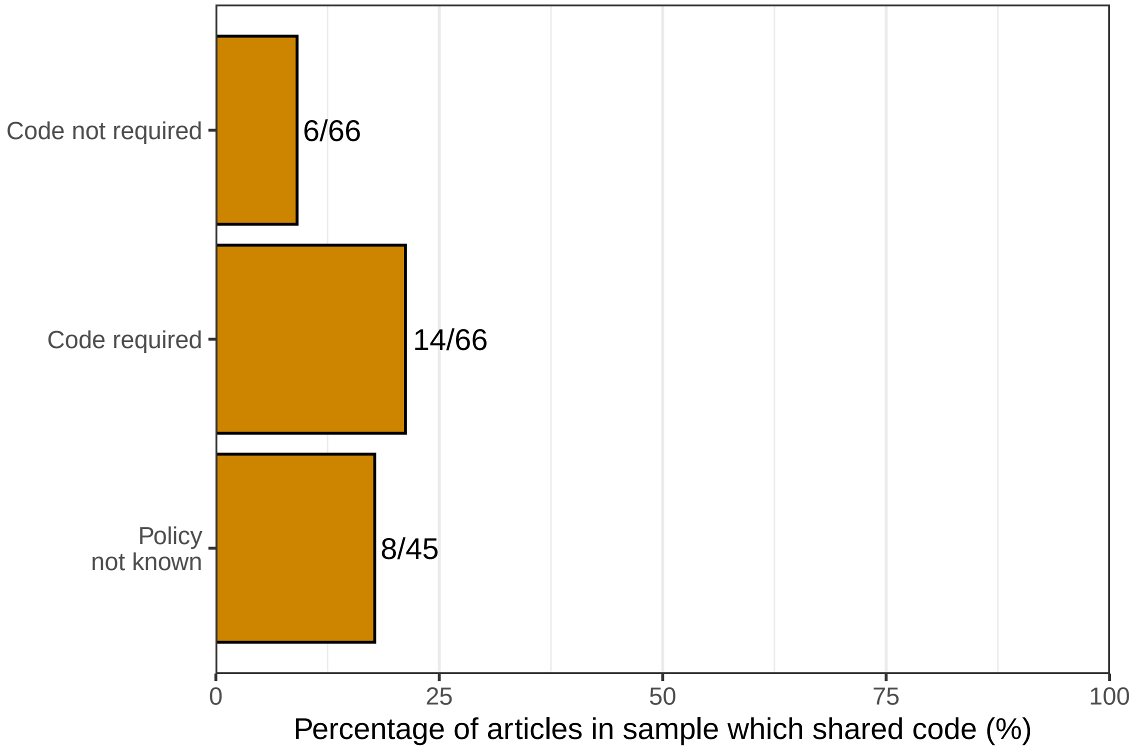

Figure 5: Comparison of code sharing rates in articles by journal code policy according to (17).

7 SOFTWARE MENTIONED IN ARTICLES

Figure 6 shows the distribution of the number of different software packages mentioned in each article (or in its supplementary material). Here, R packages have been treated as special cases: articles mentioning multiple R packages have been treated as just mentioning the R software environment. For example, an article which mentioned the R software environment and four R packages was regarded as mentioning one software package (the R software environment) rather than five software packages.

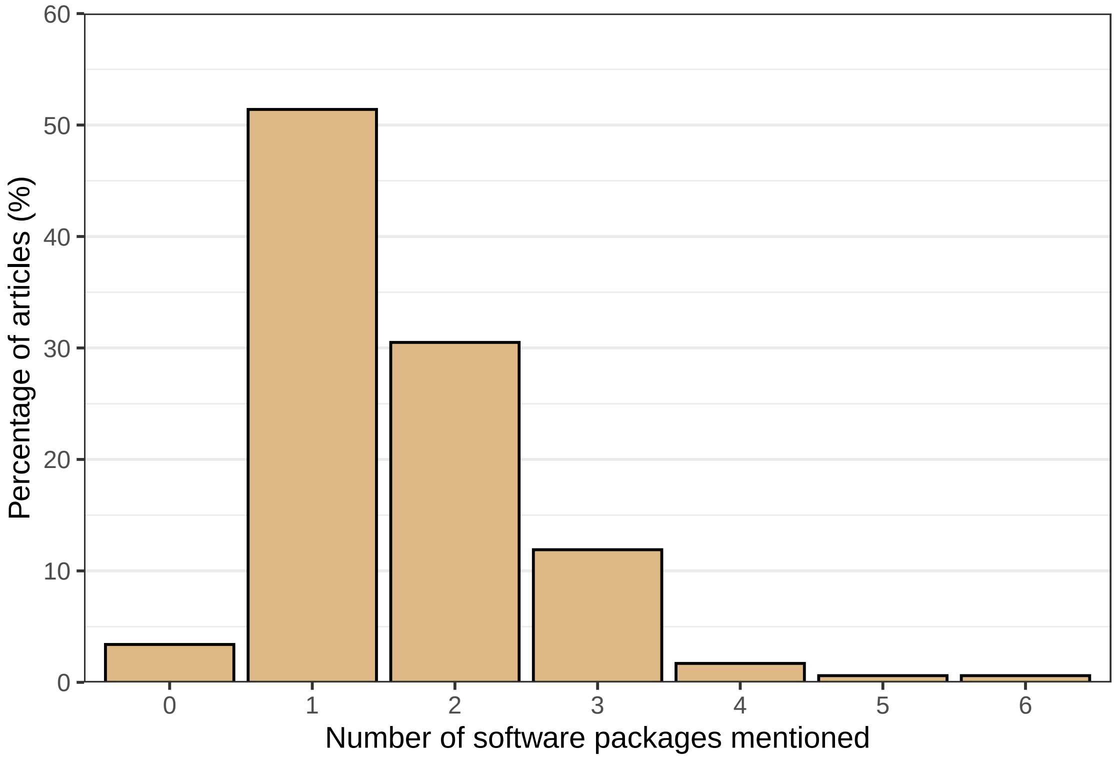

Figure 6: Distribution of the number of different software packages mentioned in each article (or its supplementary material).

Figure 7 is a version of Figure 6 which shows the distribution of the number of different software packages mentioned in each article (or in its supplementary material), *including* mentions of R packages. For example, an article which mentioned the R software environment and four R packages was regarded as mentioning five software packages rather than one software package (i.e., the R software in general).

Table 7 lists all software packages mentioned in the 177 meta-analysis articles. This table includes all mentions of the R software environment, but specifically excludes mentions of R packages, which are listed in the following table.

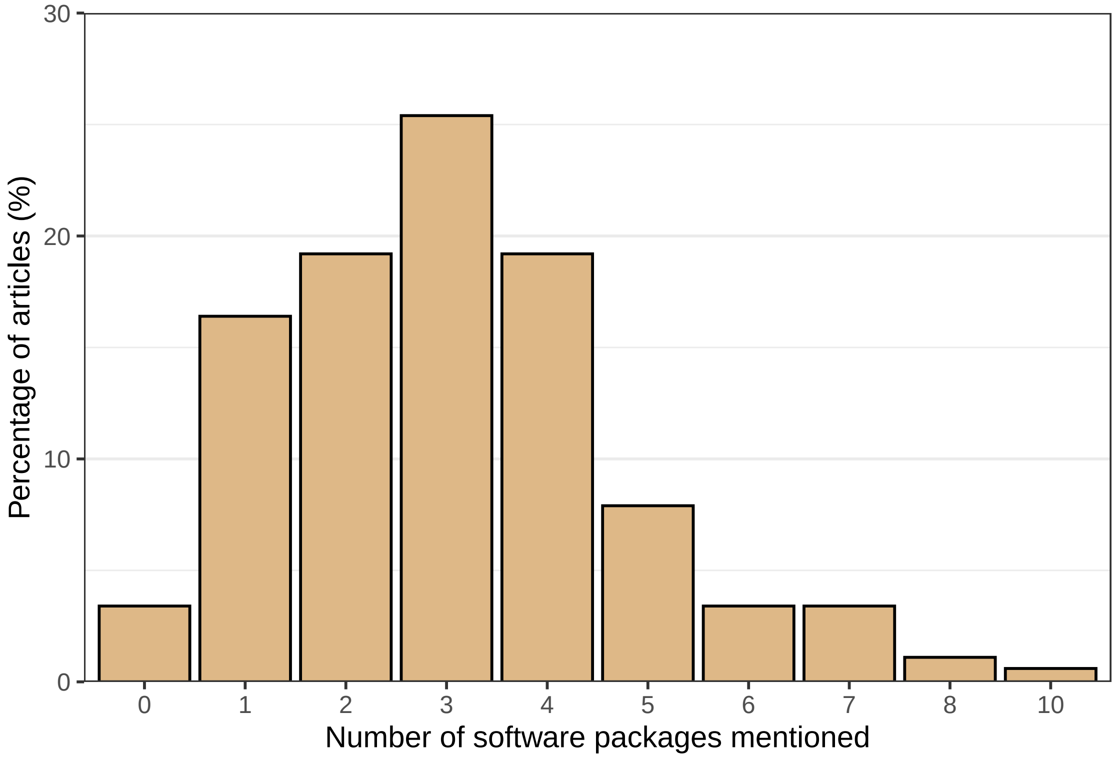

Figure 7: Distribution of the number of different software packages mentioned in each article (or its supplementary material), including mentions of R packages.

Table 7: All software packages mentioned in the 177 meta-analysis articles. Note that this table does not list individual R packages.

Name of software package	N	%
R	141	79.7
MetaWin	20	11.3
WebPlotDigitizer	10	5.6
DataThief III	9	5.1
SAS	9	5.1
ImageJ	7	4.0
GraphClick	6	3.4
PlotDigitizer	5	2.8
GetData Graph Digitizer	4	2.3
JMP	4	2.3
RStudio	4	2.3
SPSS/PASW	4	2.3
Minitab	3	1.7

Name of software package	N	%
Phylocom	3	1.7
Phylomatic	3	1.7
RAxML	3	1.7
ArcGIS	2	1.1
ArcMap	2	1.1
CMA	2	1.1
Engauge Digitizer	2	1.1
GENALEX	2	1.1
MAFFT	2	1.1
Python	2	1.1
Stan	2	1.1
AbstrackR	1	0.6
ADZE	1	0.6
AMOS	1	0.6
ARLSUMSTAT	1	0.6
ASReml-R	1	0.6
Bowtie2	1	0.6
Cervus	1	0.6
Circuitscape	1	0.6
Cytoscape	1	0.6
Digitize It 2010	1	0.6
Ecopath	1	0.6
ED2 (FORTRAN)	1	0.6
Excel	1	0.6
FigTree	1	0.6
GenClone	1	0.6
GrabIt! XP	1	0.6
GRASS GIS	1	0.6
Image Pro Plus	1	0.6
JAGS	1	0.6

Name of software package	N	%
LocARNA	1	0.6
MEGA 4	1	0.6
Mesquite	1	0.6
Modeltest	1	0.6
MrBayes	1	0.6
OpenBUGS	1	0.6
OriginPro	1	0.6
Perl	1	0.6
Photoshop	1	0.6
phyloMeta	1	0.6
PRIMER	1	0.6
QGIS	1	0.6
SigmaPlot	1	0.6
Techdig	1	0.6
xyscan	1	0.6

In total, there were 398 mentions of R and R packages across the articles: 141 mentions of the R software environment, and 257 mentions of specific R packages. Figure 8 shows the distribution of the number of packages mentioned by each R-using article. As the figure shows, it was most common for R-using articles to mention only one or two packages (68%); only 6% of R-using articles mentioned more than three R packages.

Table 8 lists all R packages mentioned in the 141 meta-analysis articles that mentioned using R. The table includes the location of each R package (whether CRAN, Bioconductor, a base R package, or from another source). Note: At the time of checking (2nd August 2022), four packages (*empiricalFDR.DESeq2*, *foodweb*, *MAc*, and *VIF*) have been removed from CRAN[3]. The vast majority (74, or 92%) of the mentioned R packages were from the Comprehensive R Archive Network (CRAN), with 3 (4%) from the Bioconductor project and 2 from other websites. One article mentioned the package *stats*, which is part of the "base" set of R packages that are an integral part of the R software.

[3]Package *empiricalFDR.DESeq2* was archived 13th June 2022 (`https://cran.r-project.org/package=empiricalFDR.DESeq2`); package *foodweb* was archived 21st June 2022 (`https://cran.r-project.org/package=foodweb`); package *MAc* was archived 4th March 2022 (`https://cran.r-project.org/package=MAc`); package *VIF* was archived 9th May 2022 (`https://cran.r-project.org/package=VIF`).

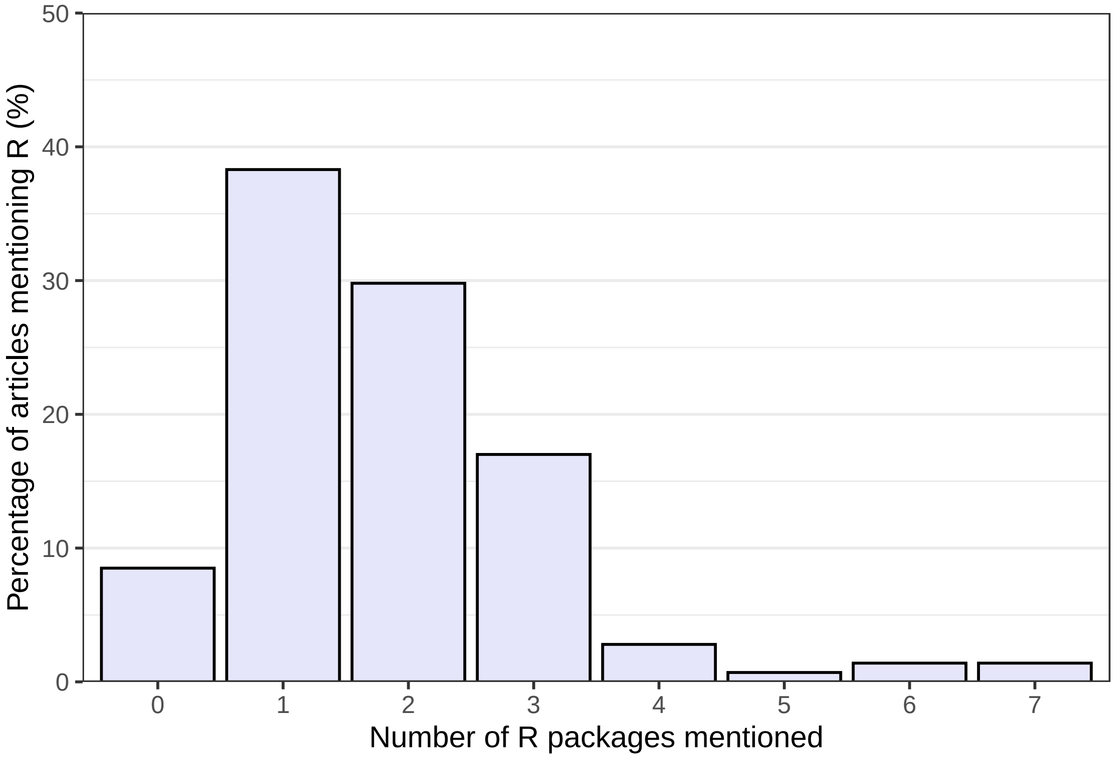

Figure 8: Distribution of the number of different R packages mentioned in each article which mentioned using R.

Table 8: All R packages mentioned in the sample of 141 meta-analysis articles which mentioned using R.

Name of R package	Package source	N	%
metafor	CRAN	75	53.2
MCMCglmm	CRAN	26	18.4
lme4	CRAN	20	14.2
ape	CRAN	13	9.2
MuMIn	CRAN	8	5.7
vegan	CRAN	7	5.0
nlme	CRAN	6	4.3
ggplot2	CRAN	5	3.5
phytools	CRAN	5	3.5
compute.es	CRAN	4	2.8
glmulti	CRAN	4	2.8
multcomp	CRAN	3	2.1
raster	CRAN	3	2.1
ade4	CRAN	2	1.4

Name of R package	Package source	N	%
boot	CRAN	2	1.4
lmerTest	CRAN	2	1.4
meta	CRAN	2	1.4
mgcv	CRAN	2	1.4
mice	CRAN	2	1.4
party	CRAN	2	1.4
picante	CRAN	2	1.4
randomForest	CRAN	2	1.4
rjags	CRAN	2	1.4
rmeta	CRAN	2	1.4
A3	CRAN	1	0.7
abc	CRAN	1	0.7
adegenet	CRAN	1	0.7
AICcmodavg	CRAN	1	0.7
arrayQualityMetrics	BioConductor	1	0.7
betareg	CRAN	1	0.7
caper	CRAN	1	0.7
coda	CRAN	1	0.7
coin	CRAN	1	0.7
DESeq2	BioConductor	1	0.7
dismo	CRAN	1	0.7
ecodist	CRAN	1	0.7
effects	CRAN	1	0.7
empiricalFDR.DESeq2	CRAN	1	0.7
foodweb	CRAN	1	0.7
gbm	CRAN	1	0.7
GENHET	other	1	0.7
Hmisc	CRAN	1	0.7
ICC	CRAN	1	0.7
igraph	CRAN	1	0.7

Name of R package	Package source	N	%
inext	CRAN	1	0.7
Kendall	CRAN	1	0.7
KOGMWU	CRAN	1	0.7
languageR	CRAN	1	0.7
leaps	CRAN	1	0.7
lmodel2	CRAN	1	0.7
lsmeans	CRAN	1	0.7
MAc	CRAN	1	0.7
maps	CRAN	1	0.7
maptools	CRAN	1	0.7
MASS	CRAN	1	0.7
merTools	CRAN	1	0.7
metahdep	BioConductor	1	0.7
MODISTools	CRAN	1	0.7
pez	CRAN	1	0.7
pheatmap	CRAN	1	0.7
plotmcmc	CRAN	1	0.7
plyr	CRAN	1	0.7
PVR	CRAN	1	0.7
R2WinBUGS	CRAN	1	0.7
rfPermute	CRAN	1	0.7
rgdal	CRAN	1	0.7
RInSp	CRAN	1	0.7
rms	CRAN	1	0.7
rotl	CRAN	1	0.7
rstan	CRAN	1	0.7
rvest	CRAN	1	0.7
segmented	CRAN	1	0.7
shape	CRAN	1	0.7
smatr	CRAN	1	0.7

Name of R package	Package source	N	%
STANDARICH	other	1	0.7
stats	base	1	0.7
vif	CRAN	1	0.7
visreg	CRAN	1	0.7
weights	CRAN	1	0.7
zoo	CRAN	1	0.7

Table 9 shows all R versions mentioned in the articles, as they originally appeared in the articles. This includes one article where the authors mention using two different versions of R for their study (v2.14.1 and v3.0.0), a study which included the R version twice, first in the body of the text and second as part of the citation in the references section, but where the versions differed (v3.1.0 and v3.0.1, which might be due to a typing error), and six articles where the version information provided was not complete (v2.12, v2.13, v2.14, v2.15, v3.1, v3.2). In the case of the six incomplete R version statements, it is possible that the authors were intending to refer to the "0" versions, i.e., 2.12.0, 2.13.0, etc.

R version	N	%
2.12	1	0.7
2.13	1	0.7
2.14	1	0.7
2.14.1	4	2.8
2.15	1	0.7
2.15.2	3	2.1
3.0.0/2.14.1	1	0.7
3.0.1	8	5.7
3.0.2	14	9.9
3.0.3	3	2.1
3.1	2	1.4
3.1.0	2	1.4
3.1.0/3.0.1	1	0.7
3.1.1	4	2.8
3.1.2	12	8.5
3.1.3	3	2.1
3.2	1	0.7
3.2.0	1	0.7
3.2.1	6	4.3
3.2.2	6	4.3
3.2.3	6	4.3
3.2.4	1	0.7
3.3.0	2	1.4
3.3.1	1	0.7
3.3.2	1	0.7
3.4.0	1	0.7
3.4.1	1	0.7
(No version mentioned)	53	37.6

Table 9: All R versions as originally mentioned in the sample of 141 meta-analysis articles which mentioned using R.

Variable	Value
ID	MA092
Study	(88)
Result source	in text and from Table 1 (pp.84-85)
Result type	Regression model results for all data
Regression result	$\text{TLP} = -4.67 + 0.725 \times \log(\text{SLA}) - 0.937 \times \log(\text{WD})$
N	68
R^2_{adj}	0.32 (p-value < 0.001)
RMSE	0.55

Table 10: Details of the target result for article MA092, (88). For context, TLP – turgor loss point, SLA – specific leaf area, WD – wood density, RMSE – root mean square error.

Variable	Value
ID	MA094
Study	(90)
Result source	in text (p.1227)
Result type	Ordination analysis result
N	n.s.
R^2	0.494 ($p < 0.0001$)

Table 11: Details of the target result for article MA094, (90). n.s. – not stated.

Variable	Value
ID	MA129
Study	(120)
Result source	Table 1 (A) (p.444)
Result type	Comparison of models by AICc
Result values	See Table 13

Table 12: Summary of the target result for article MA129, (120).

Buffer	Δ_i	w_i	R^2
Null	0.00	0.20	
50	0.96	0.12	
25	0.98	0.12	
75	1.17	0.11	
200	1.34	0.10	
150	1.56	0.09	
10	1.56	0.09	
100	1.61	0.09	
5	1.70	0.08	

Table 13: Details of the target result for article MA129, (120). The table headings and values are taken directly from Table 1 (A), p.444. Note that blank/missing values in the R^2 column are as per the original table. Here, Buffer is radius in km, Δ_i is AICc$_i$ − minimum AICc (where AICc is the corrected Akaike information criterion), w_i is Akaike weight, R^2 is coefficient of determination, omitted in this table.

Variable	Value
ID	MA212
Study	(182)
Result source	Table 2 (p.38)
Result type	Counts of matches
Result values	See Table 15

Table 14: Summary of the target result for article MA212, (182).

KS index	Match impact	Match biomass	No match	Overall match
KS_1	91	10	0	match impact
KS_2	5	81	15	match biomass
KS_3	50	28	23	
KS_4	25	54	22	match biomass
KS_5	86	12	3	match impact
KS_6	0	94	7	match biomass
KS_7	32	35	34	
KS_8	11	70	20	match biomass
KS_9	91	10	0	match impact
KS_{10}	25	54	22	match biomass
KS_{11}	71	20	10	match impact
KS_{12}	46	39	16	

Table 15: Details of the target result for article MA212, (182). The table headings and values are taken directly from Table 2, p.38. Note that blank/missing values in rows 3, 7, and 12 of column "Overall match" are as per the original table.

9 REPRODUCIBILITY REPORTS

Reproducibility report design

We decided to record all the steps of each reproduction attempt in a document integrating the running of analysis code with explanatory prose to contextualise the attempt and its outcome. This an attempt to follow the literate programming approach (211), which emphasises that computer programs ought to be human-readable and understandable. It's been recognised that this integration of analysis code and word processing facilitates reproducibility (212; 213), especially when the document is packaged with the data files required for the analysis into a compendium (214). As reported in section "Rates of data and code sharing" of the results in the main article, nearly all code that was shared was code for the R language (215). We mention this here because this fact determined the specifics of our technical approach to constructing the reproducibility reports.

We wrote a reproducibility report template using RMarkdown, a format for reproducible documents in the R language. An RMarkdown file can be compiled to produce a formatted, human-readable output document (such as an HTML or PDF document), which reports the results of running all included R code. The R source code in the RMarkdown document is re-run each time the document is compiled. We structured the template similarly to the RMarkdown reproducibility reports used in (216) to reproduce results from articles published in *Psychological Science*. Each report was structured as follows:

- A reference to the article and numerical details of the target result to be reproduced;
- Details of the shared data and code files;
- As assessment of the applicability of the shared data and code files;
- Set up of the R environment as required for the analyses;
- Importing and cleaning of data;
- Running the analysis code to reproduce the target result;
- Comparison of the original and reproduced target result value(s);
- A summary of information about the R computational environment used.

Within the RMarkdown source file, each report section consists of a combination of text marked up for appropriate formatting and "chunks" of R code which, when executed, perform in order the relevant tasks for the analysis (e.g., importing data from a file).

We set up each reproducibility report to run within its own Docker container. A container is a structured package of software designed to run a particular application in a virtual computing environment. The advantage of this approach is that applications can run on different computers without users needing to deal with software or system dependencies or settings. Docker is a tool for creating and running containers (217; 218). In particular, Docker allows users to build upon existing containers in an easy way. We created a container for each reproducibility report by starting with a pre-built container running R maintained by the Rocker project (219). The Rocker container already included all elements required to run an R session in an isolated computational environment. On top of this pre-built "layer" we built containers which installed all additional R packages required for the analyses in the reproducibility reports, including custom functions written by us to facilitate comparison of the original and reproduced values. We controlled the versions of both R and all R packages: the Rocker project maintains multiple containers with different versions of R; we selected version 3.5.0. We installed R packages from a snapshot of the Comprehensive R Archive Network (CRAN) frozen at 2 July 2018, to ensure compatibility with R 3.5.0[4] The final layer of the container for each reproducibility report incorporated the specific data and code files required for data analysis. The result of this work was a small, self-contained application with everything required to compile the reproducibility report for each of the articles with shared data and code. This is a variation on the "research compendium" (220): a research compendium is usually envisioned as being created by the original authors of a research project, to facilitate the reproducibility of their own results, rather than being created by a third party after the fact.

Running code

The core of each reproducibility report was the section which conducted the data analysis and calculated the target result. Because each reproducibility report is fundamentally an assessment of the shared data and code, we envisioned that each report would by default only execute lines of code taken directly from the shared code file(s) except where unavoidable. Importing data files was the principle situation where we anticipated we would need to modify lines of code and/or write new code.[5] In order to differentiate original lines of code from additional lines of code written by us, we wrote a function to specify which particular lines of an external code file to execute. This way, the original shared code could be run by a call to a function, rather than needing to be manually inserted into the source of the RMarkdown report. All additional, custom

[4]The date 2 July 2018 is the last day before the release of the succeeding version of R. We used the Microsoft mirror of CRAN: https://cran.microsoft.com/snapshot/2018-07-02/.

[5]We anticipated that most if not all shared code concerning operations involving external files would require modification. This was due to the fact that at the very least, file paths to data files, etc. would need to be changed to match the file system structure set up within each Docker container.

code would be written directly into the RMarkdown source file. Running individual lines of code from the original files in this way also had the advantage that only the code that was required to calculate the target result could be run, rather than the entire code file. For analyses that involved random number generation, we set an arbitrary random seed so that the specific set of numbers calculated would be reproduced over successive compilations of the report.

Table 16: The original and reproduced values of all target results.

ID	Study	Result type	Effect size type	Target result	Value type	Original	Reproduced	Percent error (%)	Status
MA016	(28)	correlation	Pearson's *r*	point est.	N	-0.83	-0.83	0.00	E
MA016	(28)	correlation	Pearson's *r*	*p*-value	N	< 0.001	< 0.001		E
MA016	(28)	correlation	Pearson's *r*	*N*	N	49	49	0.00	E
MA060	(65)	mean	Fisher *z*-transformation	point est.	N	0.044	0.043	2.27	R
MA060	(65)	mean	Fisher *z*-transformation	HPDI lower	N	-0.174	-0.194	11.49	10%+
MA060	(65)	mean	Fisher *z*-transformation	HPDI upper	N	0.289	0.268	7.27	< 10%
MA060	(65)	mean	Fisher *z*-transformation	*N*	N	37	37	0.00	E
MA062	(67)	mean	Hedges' *d*	point est.	N	-0.205	-0.204	0.49	R
MA062	(67)	mean	Hedges' *d*	CI lower	N	-0.444	-0.446	0.45	< 10%
MA062	(67)	mean	Hedges' *d*	CI upper	N	0.035	0.039	11.43	10%+
MA062	(67)	mean	Hedges' *d*	*N*	N	37	37	0.00	E
MA065	(70)	mean	Hedges' *g*	point est.	N	-8.42	-8.87	5.34	< 10%
MA065	(70)	mean	Hedges' *g*	CI lower	N	-10.73	-10.85	1.12	< 10%
MA065	(70)	mean	Hedges' *g*	CI upper	N	-6.63	-6.68	0.75	< 10%
MA065	(70)	mean	Hedges' *g*	*N*	N	703	703	0.00	E
MA067	(72)	mean	Hedges' *g*	point est.	N	-0.21	-0.21	0.00	E

ID	Study	Result type	Effect size type	Target result	Value type	Original	Reproduced	Percent error (%)	Status
MA067	(72)	mean	Hedges' *g*	SE	N	0.07	0.07	0.00	E
MA067	(72)	mean	Hedges' *g*	*z*-score	N	-2.7	-2.8	3.70	R
MA067	(72)	mean	Hedges' *g*	*p*-value	N	0.006	0.005	16.67	R
MA067	(72)	mean	Hedges' *g*	*N*	N	52	52	0.00	E
MA068	(73)	mean	odds ratio	point est.	N	1.82			F
MA068	(73)	mean	odds ratio	HPDI lower	N	1.37			F
MA068	(73)	mean	odds ratio	HPDI upper	N	2.41			F
MA068	(73)	mean	odds ratio	*N*	N	75			F
MA071	(76)	mean	response ratio	point est.	N	-0.26	-0.27	3.85	R
MA071	(76)	mean	response ratio	CI lower	N	-1.02	-1.03	0.98	R
MA071	(76)	mean	response ratio	CI upper	N	0.51	0.49	3.92	< 10%
MA071	(76)	mean	response ratio	*N*	N	50	50	0.00	E
MA074	(77)	correlation	Pearson's *r*	point est.	N	0.183	0.185	1.09	< 10%
MA074	(77)	correlation	Pearson's *r*	CI lower	N	0.089	0.089	0.00	E
MA074	(77)	correlation	Pearson's *r*	CI upper	N	0.274	0.281	2.55	< 10%
MA074	(77)	correlation	Pearson's *r*	*N*	N	43	43	0.00	E
MA081	(84)	mean	slope parameter	point est.	N	1.30	1.30	0.00	E
MA081	(84)	mean	slope parameter	CI lower	N	0.95			F
MA081	(84)	mean	slope parameter	CI upper	N	1.66			F
MA081	(84)	mean	slope parameter	*N*	N	1296	1296	0.00	E
MA091	(87)	mean	Cohen's *d*	point est.	N	0.56	0.56	0.00	E
MA091	(87)	mean	Cohen's *d*	CI lower	N	0.42	0.42	0.00	E

ID	Study	Result type	Effect size type	Target result	Value type	Original	Reproduced	Percent error (%)	Status
MA091	(87)	mean	Cohen's *d*	CI upper	N	0.69	0.69	0.00	E
MA091	(87)	mean	Cohen's *d*	*N*	N	65	65	0.00	E
MA092	(88)	model output	n.a.	R^2_{adj}	N	0.32	0.33	3.13	R
MA092	(88)	model output	n.a.	RMSE	N	0.55	0.55	0.00	E
MA092	(88)	model output	n.a.	intercept	N	-4.67	-4.18	10.49	10%+
MA092	(88)	model output	n.a.	log(SLA) coeff.	N	0.725	0.730	0.69	< 10%
MA092	(88)	model output	n.a.	log(WD) coeff.	N	-0.937	-0.980	4.59	< 10%
MA092	(88)	model output	n.a.	*N*	N	68	68	0.00	E
MA094	(90)	model output	n.a.	R^2	N	0.494			F
MA094	(90)	model output	n.a.	*p*-value	N	< 0.0001			F
MA095	(91)	mean	Fisher *z*-transformation	point est.	N	0.76	0.76	0.00	E
MA095	(91)	mean	Fisher *z*-transformation	CI lower	N	0.61	0.61	0.00	E
MA095	(91)	mean	Fisher *z*-transformation	CI upper	N	0.91	0.91	0.00	E
MA095	(91)	mean	Fisher *z*-transformation	*N*	N	25	25	0.00	E
MA126	(117)	mean	log odds ratio	point est.	N	-1.11	-1.11	0.00	E
MA126	(117)	mean	log odds ratio	SE	N	0.49	0.49	0.00	E
MA126	(117)	mean	log odds ratio	CI lower	N	-2.06	-2.06	0.00	E
MA126	(117)	mean	log odds ratio	CI upper	N	-0.15	-0.15	0.00	E

ID	Study	Result type	Effect size type	Target result	Value type	Original	Reproduced	Percent error (%)	Status
MA126	(117)	mean	log odds ratio	*z*-score	N	-2.28	-2.28	0.00	E
MA126	(117)	mean	log odds ratio	*p*-value	N	0.023	0.023	0.00	E
MA129	(120)	Table 1A, rank1	n.a.	Δ_i	N	0.00	0.00		E
MA129	(120)	Table 1A, rank1	n.a.	w_i	N	0.20	0.20	0.00	E
MA129	(120)	Table 1A, rank1	n.a.	buffer (km radius)	C	Null	Null		E
MA129	(120)	Table 1A, rank2	n.a.	Δ_i	N	0.96	0.96	0.00	E
MA129	(120)	Table 1A, rank2	n.a.	w_i	N	0.12	0.12	0.00	E
MA129	(120)	Table 1A, rank2	n.a.	buffer (km radius)	C	50	50		E
MA129	(120)	Table 1A, rank3	n.a.	Δ_i	N	0.98	0.98	0.00	E
MA129	(120)	Table 1A, rank3	n.a.	w_i	N	0.12	0.12	0.00	E
MA129	(120)	Table 1A, rank3	n.a.	buffer (km radius)	C	25	25		E
MA129	(120)	Table 1A, rank4	n.a.	Δ_i	N	1.17	1.17	0.00	E
MA129	(120)	Table 1A, rank4	n.a.	w_i	N	0.11	0.11	0.00	E
MA129	(120)	Table 1A, rank4	n.a.	buffer (km radius)	C	75	75		E
MA129	(120)	Table 1A, rank5	n.a.	Δ_i	N	1.34	1.34	0.00	E
MA129	(120)	Table 1A, rank5	n.a.	w_i	N	0.10	0.10	0.00	E
MA129	(120)	Table 1A, rank5	n.a.	buffer (km radius)	C	200	200		E

ID	Study	Result type	Effect size type	Target result	Value type	Original	Reproduced	Percent error (%)	Status
MA129	(120)	Table 1A, rank6	n.a.	Δ_i	N	1.56	1.56	0.00	E
MA129	(120)	Table 1A, rank6	n.a.	w_i	N	0.09	0.09	0.00	E
MA129	(120)	Table 1A, rank6	n.a.	buffer (km radius)	C	150	150		E
MA129	(120)	Table 1A, rank7	n.a.	Δ_i	N	1.56	1.56	0.00	E
MA129	(120)	Table 1A, rank7	n.a.	w_i	N	0.09	0.09	0.00	E
MA129	(120)	Table 1A, rank7	n.a.	buffer (km radius)	C	10	10		E
MA129	(120)	Table 1A, rank8	n.a.	Δ_i	N	1.61	1.61	0.00	E
MA129	(120)	Table 1A, rank8	n.a.	w_i	N	0.09	0.09	0.00	E
MA129	(120)	Table 1A, rank8	n.a.	buffer (km radius)	C	100	100		E
MA129	(120)	Table 1A, rank9	n.a.	Δ_i	N	1.70	1.70	0.00	E
MA129	(120)	Table 1A, rank9	n.a.	w_i	N	0.08	0.08	0.00	E
MA129	(120)	Table 1A, rank9	n.a.	buffer (km radius)	C	5	5		E
MA145	(128)	mean	Fisher *z*-transformation	point est.	N	-0.08	-0.08	0.00	E
MA145	(128)	mean	Fisher *z*-transformation	HPDI lower	N	-0.22	-0.21	4.55	R
MA145	(128)	mean	Fisher *z*-transformation	HPDI upper	N	0.03	0.05	66.67	10%+

ID	Study	Result type	Effect size type	Target result	Value type	Original	Reproduced	Percent error (%)	Status
MA145	(128)	mean	Fisher *z*-transformation	N	N	118	118	0.00	E
MA145	(128)	mean	Fisher *z*-transformation	$N_{studies}$	N	38	38	0.00	E
MA145	(128)	mean	Fisher *z*-transformation	$N_{species}$	N	25	25	0.00	E
MA147	(130)	mean	percentage	point est.	N	0.13	0.13	0.00	E
MA147	(130)	mean	percentage	SE	N	0.03	0.03	0.00	E
MA147	(130)	mean	percentage	CI lower	N	0.074	0.074	0.00	E
MA147	(130)	mean	percentage	CI upper	N	0.19	0.19	0.00	E
MA147	(130)	mean	percentage	N	N	49	49	0.00	E
MA155	(137)	correlation	Pearson's r	point est.	N	0.51	0.51	0.00	E
MA155	(137)	correlation	Pearson's r	p-value	N	0.01	0.01	0.00	E
MA188	(160)	mean	Log response ratio	point est.	N	-0.363	-0.363	0.00	E
MA188	(160)	mean	Log response ratio	CI lower	N	-0.408	-0.408	0.00	E
MA188	(160)	mean	Log response ratio	CI upper	N	-0.318	-0.318	0.00	E
MA188	(160)	mean	Log response ratio	N	N	818	818	0.00	E
MA191	(163)	mean	allometric slope parameter	point est.	N	0.86	0.85	1.16	R
MA191	(163)	mean	allometric slope parameter	CI lower	N	0.77	0.77	0.00	E

ID	Study	Result type	Effect size type	Target result	Value type	Original	Reproduced	Percent error (%)	Status
MA191	(163)	mean	allometric slope parameter	CI upper	N	0.94	0.94	0.00	E
MA191	(163)	mean	allometric slope parameter	*N*	N	553	553	0.00	E
MA198	(169)	mean	Fisher *z*-transformation	point est.	N	-0.41	-0.42	2.44	R
MA198	(169)	mean	Fisher *z*-transformation	CI lower	N	-0.55	-0.55	0.00	E
MA198	(169)	mean	Fisher *z*-transformation	CI upper	N	-0.27	-0.28	3.70	R
MA198	(169)	mean	Fisher *z*-transformation	*N*	N	79	80	1.27	< 10%
MA202	(173)	mean	Hedges' *d*	point est.	N	-0.330	-0.340	3.03	< 10%
MA202	(173)	mean	Hedges' *d*	CI lower	N	-0.503	-0.521	3.58	< 10%
MA202	(173)	mean	Hedges' *d*	CI upper	N	-0.156	-0.159	1.92	< 10%
MA202	(173)	mean	Hedges' *d*	*N*	N	329	329	0.00	E
MA211	(181)	mean	log response ratio	point est.	N	0.24			F
MA211	(181)	mean	log response ratio	CI lower	N	0.23			F
MA211	(181)	mean	log response ratio	CI upper	N	0.25			F
MA211	(181)	mean	log response ratio	*N*	N	3298			F
MA212	(182)	Table 2, Match biomass	n.a.	KS1	N	10	10	0.00	E

ID	Study	Result type	Effect size type	Target result	Value type	Original	Reproduced	Percent error (%)	Status
MA212	(182)	Table 2, Match biomass	n.a.	KS2	N	81	81	0.00	E
MA212	(182)	Table 2, Match biomass	n.a.	KS3	N	28	28	0.00	E
MA212	(182)	Table 2, Match biomass	n.a.	KS4	N	54	54	0.00	E
MA212	(182)	Table 2, Match biomass	n.a.	KS5	N	12	12	0.00	E
MA212	(182)	Table 2, Match biomass	n.a.	KS6	N	94	94	0.00	E
MA212	(182)	Table 2, Match biomass	n.a.	KS7	N	35	35	0.00	E
MA212	(182)	Table 2, Match biomass	n.a.	KS8	N	70	70	0.00	E
MA212	(182)	Table 2, Match biomass	n.a.	KS9	N	10	10	0.00	E
MA212	(182)	Table 2, Match biomass	n.a.	KS10	N	54	54	0.00	E
MA212	(182)	Table 2, Match biomass	n.a.	KS11	N	20	20	0.00	E
MA212	(182)	Table 2, Match biomass	n.a.	KS12	N	39	39	0.00	E

ID	Study	Result type	Effect size type	Target result	Value type	Original	Reproduced	Percent error (%)	Status
MA212	(182)	Table 2, Match impact	n.a.	KS1	N	91	91	0.00	E
MA212	(182)	Table 2, Match impact	n.a.	KS2	N	5	5	0.00	E
MA212	(182)	Table 2, Match impact	n.a.	KS3	N	50	51	2.00	< 10%
MA212	(182)	Table 2, Match impact	n.a.	KS4	N	25	25	0.00	E
MA212	(182)	Table 2, Match impact	n.a.	KS5	N	86	87	1.16	< 10%
MA212	(182)	Table 2, Match impact	n.a.	KS6	N	0	0		E
MA212	(182)	Table 2, Match impact	n.a.	KS7	N	32	33	3.12	< 10%
MA212	(182)	Table 2, Match impact	n.a.	KS8	N	11	11	0.00	E
MA212	(182)	Table 2, Match impact	n.a.	KS9	N	91	91	0.00	E
MA212	(182)	Table 2, Match impact	n.a.	KS10	N	25	25	0.00	E
MA212	(182)	Table 2, Match impact	n.a.	KS11	N	71	72	1.41	< 10%

ID	Study	Result type	Effect size type	Target result	Value type	Original	Reproduced	Percent error (%)	Status
MA212	(182)	Table 2, Match impact	n.a.	KS12	N	46	47	2.17	< 10%
MA212	(182)	Table 2, No match	n.a.	KS1	N	0	0		E
MA212	(182)	Table 2, No match	n.a.	KS2	N	15	15	0.00	E
MA212	(182)	Table 2, No match	n.a.	KS3	N	23	22	4.35	< 10%
MA212	(182)	Table 2, No match	n.a.	KS4	N	22	22	0.00	E
MA212	(182)	Table 2, No match	n.a.	KS5	N	3	2	33.33	10%+
MA212	(182)	Table 2, No match	n.a.	KS6	N	7	7	0.00	E
MA212	(182)	Table 2, No match	n.a.	KS7	N	34	33	2.94	< 10%
MA212	(182)	Table 2, No match	n.a.	KS8	N	20	20	0.00	E
MA212	(182)	Table 2, No match	n.a.	KS9	N	0	0		E
MA212	(182)	Table 2, No match	n.a.	KS10	N	22	22	0.00	E
MA212	(182)	Table 2, No match	n.a.	KS11	N	10	9	10.00	10%+
MA212	(182)	Table 2, No match	n.a.	KS12	N	16	15	6.25	< 10%
MA212	(182)	Table 2, Overall match	n.a.	KS1	C	match impact	match impact		E
MA212	(182)	Table 2, Overall match	n.a.	KS2	C	match biomass	match biomass		E
MA212	(182)	Table 2, Overall match	n.a.	KS3	C	(none)	match impact		NC
MA212	(182)	Table 2, Overall match	n.a.	KS4	C	match biomass	match biomass		E

ID	Study	Result type	Effect size type	Target result	Value type	Original	Reproduced	Percent error (%)	Status
MA212	(182)	Table 2, Overall match	n.a.	KS5	C	match impact	match impact		E
MA212	(182)	Table 2, Overall match	n.a.	KS6	C	match biomass	match biomass		E
MA212	(182)	Table 2, Overall match	n.a.	KS7	C	(none)	(none)		E
MA212	(182)	Table 2, Overall match	n.a.	KS8	C	match biomass	match biomass		E
MA212	(182)	Table 2, Overall match	n.a.	KS9	C	match impact	match impact		E
MA212	(182)	Table 2, Overall match	n.a.	KS10	C	match biomass	match biomass		E
MA212	(182)	Table 2, Overall match	n.a.	KS11	C	match impact	match impact		E
MA212	(182)	Table 2, Overall match	n.a.	KS12	C	(none)	(none)		E
MA213	(183)	mean	difference in means	point est.	N	-0.07	-0.07	0.00	E
MA213	(183)	mean	difference in means	*p*-value	N	0.362	0.362	0.00	E
MA213	(183)	mean	difference in means	*N*	N	654	654	0.00	E
MA229	(194)	mean	log response ratio	point est.	N	0.40	0.39	2.50	R
MA229	(194)	mean	log response ratio	CI lower	N	0.24	0.26	8.33	< 10%
MA229	(194)	mean	log response ratio	CI upper	N	0.53	0.53	0.00	E

ID	Study	Result type	Effect size type	Target result	Value type	Original	Reproduced	Percent error (%)	Status
MA229	(194)	mean	log response ratio	*N*	N	57	57	0.00	E

Examining dependency between reproduced values within articles

Table 5 in the main article lists 19 articles where (i) the result type is a summary effect and (ii) the code is relevant. For these 19 articles, the set of target result values are broadly similar in type: there is a point estimate, a sample size, and some kind of measure of uncertainty (e.g., the upper and lower bounds of a confidence interval). To gauge the level of dependency between the reproductions of these different types of values within articles, Table 17 breaks down the results for each article by target value type, specifying how closely the target result value was reproduced (using the same categories reported in Table 6 in the main article). For this summary, measures of uncertainty other than confidence interval bounds (e.g., standard errors) were ignored.

ID	N	Point est.	CI lower	CI upper
MA091	Exact	Exact	Exact	Exact
MA095	Exact	Exact	Exact	Exact
MA147	Exact	Exact	Exact	Exact
MA188	Exact	Exact	Exact	Exact
MA145	Exact	Exact	Rounding	At Least 10%
MA081	Exact	Exact	Failure	Failure
MA067	Exact	Exact	n.a.	n.a.
MA213	Exact	Exact	n.a.	n.a.
MA191	Exact	Rounding	Exact	Exact
MA071	Exact	Rounding	Rounding	Within 10%
MA229	Exact	Rounding	Within 10%	Exact
MA062	Exact	Rounding	Within 10%	At Least 10%
MA060	Exact	Rounding	At Least 10%	Within 10%
MA074	Exact	Within 10%	Exact	Within 10%
MA065	Exact	Within 10%	Within 10%	Within 10%
MA202	Exact	Within 10%	Within 10%	Within 10%
MA198	Within 10%	Rounding	Exact	Rounding
MA211	Failure	Failure	Failure	Failure
MA126	n.a.	Exact	Exact	Exact

Table 17: A breakdown of how closely target result values were reproduced for each article with relevant code and a summary effect result type. The target result value types are sample size N, point estimate, and confidence interval bounds (CI lower and CI upper). Values of "n.a." indicate that that particular target result value type was not reported for that article.

Table 17 considers how closely the sample size, point estimate, lower confidence interval bound, and upper confidence interval bound could be reproduced for each article. The closeness of the reproduced values were considered progressively, from left to right. The table shows that most values of sample size could be reproduced exactly, but the closeness of the reproduced values dropped off considerably after that for the point estimate, etc. There are two identifiable clusters: a cluster of four articles (MA091, MA095, MA147, MA188) where all target values could be reproduced exactly, and a cluster of three articles (MA065, MA071,

MA202) where the sample size was reproduced exactly, and the remaining values were within 10%. This clustering may indicate that there is some dependency between values from the same article regarding how closely they will be reproduced. However, the sample is small and the categories of reproduction closeness are relatively coarse.

10 REPRODUCING TARGET RESULTS WHEN CODE NOT RELEVANT

Table 18 details the circumstances of the six cases where shared code was judged not relevant to reproducing the target result.

ID	Study	Code language	Description
MA016	(28)	Python	Not relevant. The code shared is for simulations of leaf longevity, reported separately from the meta-analysis.
MA068	(73)	R	Partially relevant. The code shared regards the extraction of effect sizes from primary studies used in the meta-analysis. The code does not conduct the meta-analysis itself.
MA092	(88)	Fortran	Not relevant. The code shared is the source code for a modified version of the Ecosystem Demography Biosphere Model, ED2 (221). Simulations using this model were reported separately from the meta-analysis.
MA094	(90)	R	Partially relevant. The code shared is for generating null food web models. Although necessary, the code is not sufficient to reproduce the chosen result. Further, there was a "missing" code file: in the Oikos online appendix, one listed code file was actually missing (`hierarchy_measure.R`), while the other listed code file (`null_models.R`) was duplicated, resulting in two code files in the appendix with the same contents.
MA155	(137)	R	Not relevant. The code shared is for conducting Gene Ontology analyses, and for producing article Figure 1D. These are separate results from the meta-analysis.
MA212	(182)	R	Partially relevant. The code shared runs Spearman rank correlation tests, relevant to meta-analysis results presented in Table 3 of article. The code is not relevant to the selected meta-analysis result.

Table 18: The articles with shared code which was either not relevant or only partially relevant to reproducing the chosen meta-analysis results.

In the cases of MA016, MA092, and MA155, the shared code had nothing to do with the reported meta-analysis results. In the case of MA212, the shared code was partially relevant, but was practically unusable for the purposes of reproducing the specific results in the article. (Specifically, the shared code for MA212, written to calculate Spearman's rank correlation coefficient for multiple sets of data and summarise the corresponding p-values, seemed to be an extract from a larger code base; the code assumed a specific data structure that was not defined anywhere in the shared materials, nor did the data structure implied by the code correspond to any of the shared data files. Lacking contextual information on the setup required for the code to work, we decided that the code as provided for MA212 was unusable.) For these four cases we attempted

to reproduce the originally selected target results detailed in either Table 4 from the main article or Table 10 for MA092 by writing entirely new R code.

There were 59 target result values across MA016, MA092, MA155, and MA212. This set of target result values included 12 non-numeric values: these were entries from the table in article MA212, see Table 15. Percent error was not applicable to these non-numeric values, and so the reproduced values were assessed as being either exact text string matches with the original or non-matches. The details of the individual reproduction attempts for all these values are reported in Table 16.

In the cases of MA068 and MA094, the code was relevant to other parts of the meta-analysis described in the articles. We selected alternative target results from these articles that were directly relevant to the shared code. For MA068, the shared code performed simulations of logistic regression slopes and standard errors; these simulations were performed to supplement under-reported results from two primary studies included in the meta-analysis. The target results were the values of the simulations as reported in the article. For MA094, the shared code simulated species richness in food webs using different food web models; the simulation results were compared with the results from a sample of published food webs in a figure. The target results were the widths of bars in that figure, which represented the average proportion of species richness at different trophic levels for the published and simulated food webs. There were 3 target result values for MA068, and 21 target result values for MA094. The details of the alternative target results are reported in Tables 19-21, and the results of comparing the reproduced values with the original values of these target results are in Table 22.

Variable	Value
ID	MA068
Study	(73)
Result source	Table 1 (pp.7-8)
Result type	Simulated slope parameters and standard errors to supplement incompletely reported primary study results
Standard error 1	0.001
Mean slope parameter 2	-0.001
Standard error 2	0.113

Table 19: Detail of the alternative target results selected for article MA068, (73). These alternative target results were selected due to being relevant to the shared code. Standard error 1 is simulated to supplement a result from (222), mean slope parameter 2 and standard error 2 are simulated to supplement a result from (223).

We used the shared code for these two articles to successfully calculate values for all 24 alternative target values. This perfect success rate is perhaps to be expected, since the alternative target results were specifically selected on the basis of being relevant to the shared code. Seven values out of the 24 (29%) were reproduced

Variable	Value
ID	MA094
Study	(90)
Result source	in text (p.1227)
Result type	Descriptive statistics of species richness (i.e., N_{species}) in a sample of published food webs
$N_{\text{food webs}}$	72
Mean	90.21
Standard deviation	31.27
Minimum	50
Maximum	209

Table 20: Detail of the first set of alternative target results selected for article MA094, (90). These alternative target results were selected due to being relevant to the shared code.

exactly (to the same precision as reported), another seven reproduced values (29%) were within 10% of the original value, and the remaining ten (42%) reproduced values were 10% or more from the original value. All ten reproduced values with substantial percent errors (10% or more) compared to the original were target results from simulations, which use pseudo-random number generation, and neither R script set a random seed which would have facilitated the exact reproduction of the simulations. For one target result in MA068, the mean slope parameter for a logistic regression, the reproduced value was 0.001, compared with an original value of −0.001. This is the only case in this study of a reproduced target result not being in the same direction as the original target result value. However, by using different random seeds, repeated simulations of this target result could yield different results, which might more closely agree with the original value.

Variable	Value
ID	MA094
Study	(90)
Result source	Figure 1 (p.1227)
Result type	Bar widths (in pixels) representing average proportions of species richness at different trophic levels for different food web types
Published food webs, top trophic level	215
Published food webs, intermediate trophic level	475
Published food webs, herbivore trophic level	430
Published food webs, basal trophic level	549
Random food webs, top trophic level	589
Random food webs, intermediate trophic level	521
Random food webs, herbivore trophic level	51
Random food webs, basal trophic level	108
Cascade food webs, top trophic level	79
Cascade food webs, intermediate trophic level	934
Cascade food webs, herbivore trophic level	158
Cascade food webs, basal trophic level	221
Niche food webs, top trophic level	441
Niche food webs, intermediate trophic level	408
Niche food webs, herbivore trophic level	102
Niche food webs, basal trophic level	385

Table 21: Detail of the second set of alternative target results selected for article MA094, (90). These alternative target results were selected due to being relevant to the shared code.

Table 22: The original and reproduced values of all alternative target results for MA068 and MA094. All target result values are numeric.

ID	Study	Result type	Effect size type	Target result	Original	Reproduced	Percent error (%)	Status
MA068	(73)	logistic regression model (Rödel)	slope parameter	SE	0.001	0.001	0.00	E
MA068	(73)	logistic regression model (Barber-Meyer)	slope parameter	point est.	-0.001	0.001	200.00	10%+
MA068	(73)	logistic regression model (Barber-Meyer)	slope parameter	SE	0.113	0.113	0.00	E
MA094	(90)	mean	species richness	point est.	90.21	90.21	0.00	E
MA094	(90)	mean	species richness	SD	31.27	31.27	0.00	E
MA094	(90)	mean	species richness	minimum	50	50	0.00	E
MA094	(90)	mean	species richness	maximum	209	209	0.00	E
MA094	(90)	mean	species richness	N	72	72	0.00	E
MA094	(90)	average proportion of species present	pixel width	published food webs, top trophic level	215	212	1.40	$<10\%$
MA094	(90)	average proportion of species present	pixel width	published food webs, intermediate trophic level	475	476	0.21	$<10\%$

ID	Study	Result type	Effect size type	Target result	Original	Reproduced	Percent error (%)	Status
MA094	(90)	average proportion of species present	pixel width	published food webs, herbivore trophic level	430	434	0.93	< 10%
MA094	(90)	average proportion of species present	pixel width	published food webs, basal trophic level	549	550	0.18	< 10%
MA094	(90)	average proportion of species present	pixel width	random food webs, top trophic level	589	96	83.70	10%+
MA094	(90)	average proportion of species present	pixel width	random food webs, intermediate trophic level	521	1078	106.91	10%+
MA094	(90)	average proportion of species present	pixel width	random food webs, herbivore trophic level	51	27	47.06	10%+
MA094	(90)	average proportion of species present	pixel width	random food webs, basal trophic level	108	64	40.74	10%+
MA094	(90)	average proportion of species present	pixel width	cascade food webs, top trophic level	79	252	218.99	10%+
MA094	(90)	average proportion of species present	pixel width	cascade food webs, intermediate trophic level	934	759	18.74	10%+
MA094	(90)	average proportion of species present	pixel width	cascade food webs, herbivore trophic level	158	160	1.27	< 10%
MA094	(90)	average proportion of species present	pixel width	cascade food webs, basal trophic level	221	227	2.71	< 10%
MA094	(90)	average proportion of species present	pixel width	niche food webs, top trophic level	441	359	18.59	10%+

ID	Study	Result type	Effect size type	Target result	Original	Reproduced	Percent error (%)	Status
MA094	(90)	average proportion of species present	pixel width	niche food webs, intermediate trophic level	408	441	8.09	< 10%
MA094	(90)	average proportion of species present	pixel width	niche food webs, herbivore trophic level	102	129	26.47	10%+
MA094	(90)	average proportion of species present	pixel width	niche food webs, basal trophic level	385	439	14.03	10%+

11 REVISITING THE DEFINITION OF REPRODUCIBILITY

In this section, we return to the definition of reproducibility provided in the introduction, "reproducibility is obtaining consistent results using the same input data; computational steps, methods, and code; and conditions of analysis" (224, p.43) and consider each component of this definition in turn, in the context of the results of this study.

Consistent As is noted in the NAS report, there can be different standards for what is considered "consistent". In some scenarios, bitwise consistency may be required. In others, obtaining results in the same direction as the original might be considered good enough. The reproduced results in this study were compared to their original counterparts by looking at the percentage error. Looking at Table 6 in the main article, relaxing standards for consistency from exact matches only to matches differing only by rounding precision and matches within 10% of the original increased the percentage of target results considered "consistent" from 43% to 57%. In the context of meta-analysis, what might be considered sufficient consistency will likely depend on the purposes that the results are put to use, and the sensitivity of those purposes to variation in the inputs. Meta-analysis in particular is an interesting case because meta-analyses can be updated with additional primary studies, and is complicated by differences of judgment over which primary studies ought to be included and excluded, etc. Given this, there may be an expectation that meta-analytic summary effects are already subject to variation beyond formal statistical error. In this context, there may be a tolerance for a certain amount of inconsistency in any asserted summary effect, such that small discrepancies of up to 10% in value when reproduced are not fatal (albeit perhaps still worthy of rigorous checking).

Results In the context of reproducing a numerical result, "results" are those numbers printed in the published article. Ordinarily, we take them as they are presented. However, in this study, we have the example of a result reported in article MA062 (67) which contains a typo (a missing minus sign). Here, the code and data produce the "correct" result, with a value less than zero. Here, interpretation and judgement is required: a reader can see that there is supposed to be a minus sign in front of the reported effect size, since that would then agree with the stated confidence interval. This example is particularly straightforward and obvious.

This is important to note because a lot of the challenge of evaluating computational reproducibility of results is in getting the data and code to "work"; understandably, that's where a lot of the focus is. But this perhaps takes for granted that the target value in the published article that is being reproduced is valid, and

has not been corrupted during rounds of revision, copy editing, type setting, etc. (This is of course the chief motivation behind reproducible reports/documents, where analysis and text are contained within the one document, and so issues such as transcription error, etc. are mitigated.)

Another issue is the coverage/completeness of results. Do all "results" (e.g., all numerical values reported in text, all tables and figures) in an article need to be reproducible? For tractability, this study selected a single target result for reproduction across a number of articles, with the goal of selecting the first mentioned summary effect where possible. Even though this "bare minimum" attempt for each article covered only a tiny proportion of all results reported, the successes and failures were still informative.

Same input data Data sharing policies and advocacy perhaps may take for granted that the data file(s) that get shared are the same as the data file(s) that were actually used for the calculations reported in the article. But, this may not necessarily be the case: (i) Authors may "clean up" their data files in preparation for them to be shared. This may involve recoding of data values, or renaming of variables to make them more explicable to outside readers. This could introduce changes to how the data needs to be pre-processed or recoded for analysis. (ii) Some data files may be updated or edited over time, especially if used in projects which span more than a single article. It may become a non-trivial task to identify a single version of the data file(s) that applies to all results reported in an article.

In one meta-analysis (181), the data file shared was not the data file used with the provided code, and according to the content of the article itself, could not have been the data file used to calculate all results reported. This was due to a missing variable in the data set.

There is one meta-analysis where the authors explicitly provide two different versions of their data: the first which is the one actually used in the meta-analysis (and so is the one to be used to "reproduce" the results in the article), and the second which is a corrected version of the first, and which the authors recommend be used for further analysis. This example is particularly striking because of the transparency of the authors and the delineation they provide between "original" results and what might be called "correct" results.

The point of this is to say that when we say "the same data", we might not necessarily mean or intend to refer to "the specific original file(s) used by the authors in the calculation of the results". What we mean is a set of data that has the same substantive content as the original data, regardless of whether that version of the data was used by the authors to calculate the results or not.

Same computational steps, methods, and code This has multiple components: first, the sharing of code files has the same issues as the sharing of data files, as detailed above. Code files may be "cleaned up" for public release, or comments may be added, or code might be passed through a tool to format the code for easier reading. The point of this is to say that when we say "the same code", we may not necessarily mean the "specific original file(s) used by the authors".

The "same computational steps" also requires some nuance: for example, it's taken as given in studies evaluating reproducibility that things like the file system paths of input files do not really count as meaningful barriers to computational reproducibility. It does not seem "fair" to declare a result as unable to be reproduced purely because the code as written assumes a different file folder structure than exists on the reproducer's computer system.

The above example of file paths seems unambiguous enough (and is very common), but "alterations to the code as supplied" exists on a spectrum: if we agree that altering the computational steps to enable files to be read is at the end of "insubstantial changes", at what point do changes to the code as supplied become substantial, and we agree that we are no longer taking the same computational steps?

Examples from this study include typos in code that once corrected produce matching results. Correcting the (perhaps obvious) typo is making an act of interpretation: we're intuiting what the original authors intended, even though it is not literally what they have written in code.

This particular example also feeds back to the "same code" issue: if an analysis script contains a typo/syntax error that does not produce the results reported in the paper, can it be "the same" code run by the authors? If it was, they would have obtained an error message instead of a result, and so could not have reported that result in the paper. This makes it clear that the shared code file is not literally the code that was run to obtain the original results.

Beneath all this, there is some notion that when we refer to computational steps, we are referring to the computational steps that "really matter" to the calculation of the result.

Same conditions of analysis Should we take this to mean the same computing and software environment? In the context of this study and its results, it seems that the conditions of analysis mostly concerns software packages and perhaps their versions—the fact that meta-analysis results were typically only reported to the third decimal place at most, as well as the observed success rate at the target result level indicates that information about computer hardware (e.g., architecture, processors) is wholly unnecessary, as is information about operating and file systems. This would not be universal across research disciplines, but it seems

reasonable for meta-analysis.

As found in this study, study authors often reported the software tools they used for analysis, even when code was not shared. Not all mentions of software were accompanied by information about versions, but this study shows that a lack of version information was not fatal to reproduction attempts, although software version differences may be contributing to the discrepancies between original and reproduced values.

One condition of analysis that clearly stood out as an issue was the lack of specification of random seeds for procedures which involved pseudo-random number generation. Without random seeds, such procedures become an inescapable source of discrepancy between original and reproduced values. Specifying a random seed can nullify this problem, however.

REFERENCES

[1] Lau J, Rothstein HR, Stewart GB. History and Progress of Meta-analysis. In: Koricheva J, Gurevitch J, Mengersen K, editors. Handbook of Meta-analysis in Ecology and Evolution. Princeton University Press; 2013. p. 407–419.

[2] Borenstein M, Hedges LV, Higgins JPT, Rothstein HR. Introduction to Meta-Analysis. Chichester, U.K.: Wiley; 2009.

[3] Green S, Higgins JP, Alderson P, Clarke M, Mulrow CD, Oxman AD. Introduction. In: Fellow JPHSSV, Director SGF, editors. Cochrane Handbook for Systematic Reviews of Interventions. John Wiley & Sons, Ltd; 2008. p. 1–9.

[4] Moher D, Liberati A, Tetzlaff J, Altman DG, Group TP. Preferred Reporting Items for Systematic Reviews and Meta-Analyses: The PRISMA Statement. PLOS Medicine. 2009;6(7):e1000097. doi:10.1371/journal.pmed.1000097.

[5] Page MJ, McKenzie JE, Bossuyt PM, Boutron I, Hoffmann TC, Mulrow CD, et al. The PRISMA 2020 statement: an updated guideline for reporting systematic reviews. BMJ. 2021;372:n71. doi:10.1136/bmj.n71.

[6] O'Dea RE, Lagisz M, Jennions MD, Koricheva J, Noble DWA, Parker TH, et al. Preferred reporting items for systematic reviews and meta-analyses in ecology and evolutionary biology: a PRISMA extension. Biological Reviews. 2021;96(5):1695–1722. doi:10.1111/brv.12721.

[7] Arnqvist G, Wooster D. Meta-analysis: synthesizing research findings in ecology and evolution. Trends in Ecology & Evolution. 1995;10(6):236–240. doi:10.1016/S0169-5347(00)89073-4.

[8] Gurevitch J, Hedges LV. Statistical Issues in Ecological Meta-Analyses. Ecology. 1999;80(4):1142–1149. doi:10.1890/0012-9658(1999)080[1142:SIIEMA]2.0.CO;2.

[9] Gurevitch J, Hedges LV. Meta-analysis: Combining the results of independent experiments. In: Scheiner SM, Gurevitch J, editors. Design and analysis of ecological experiments. 2nd ed. New York, NY: Oxford University Press; 2001. p. 347–369.

[10] Koricheva J, Gurevitch J, Mengersen K, editors. Handbook of Meta-analysis in Ecology and Evolution. Princeton University Press; 2013.

[11] Gates S. Review of methodology of quantitative reviews using meta-analysis in ecology. Journal of Animal Ecology. 2002;71(4):547–557. doi:10.1046/j.1365-2656.2002.00634.x.

[12] Nakagawa S, Santos ESA. Methodological issues and advances in biological meta-analysis. Evolutionary Ecology. 2012;26(5):1253–1274. doi:10.1007/s10682-012-9555-5.

[13] Vetter D, Rücker G, Storch I. Meta-analysis: A need for well-defined usage in ecology and conservation biology. Ecosphere. 2013;4(6):1–24. doi:10.1890/ES13-00062.1.

[14] Koricheva J, Gurevitch J. Uses and misuses of meta-analysis in plant ecology. Journal of Ecology. 2014;102(4):828–844. doi:10.1111/1365-2745.12224.

[15] ArchMiller AA, Bauer EF, Koch RE, Wijayawardena BK, Anil A, Kottwitz JJ, et al. Formalizing the definition of meta-analysis in Molecular Ecology. Molecular Ecology. 2015;24(16):4042–4051. doi:10.1111/mec.13264.

[16] Nakagawa S, Noble DWA, Senior AM, Lagisz M. Meta-evaluation of meta-analysis: ten appraisal questions for biologists. BMC Biology. 2017;15(1):18. doi:10.1186/s12915-017-0357-7.

[17] Mislan KAS, Heer JM, White EP. Elevating The Status of Code in Ecology. Trends in Ecology & Evolution. 2016;31(1):4–7. doi:10.1016/j.tree.2015.11.006.

[18] Bowles TM, Jackson LE, Loeher M, Cavagnaro TR. Ecological intensification and arbuscular mycorrhizas: a meta-analysis of tillage and cover crop effects. Journal of Applied Ecology. 2017;54(6):1785–1793. doi:10.1111/1365-2664.12815.

[19] Mori AS, Tatsumi S, Gustafsson L. Landscape properties affect biodiversity response to retention approaches in forestry. Journal of Applied Ecology. 2017;54(6):1627–1637. doi:10.1111/1365-2664.12888.

[20] Charlebois JA, Sargent RD. No consistent pollinator-mediated impacts of alien plants on natives. Ecology Letters. 2017;20(11):1479–1490. doi:10.1111/ele.12831.

[21] Martin-StPaul N, Delzon S, Cochard H. Plant resistance to drought depends on timely stomatal closure. Ecology Letters. 2017;20(11):1437–1447. doi:10.1111/ele.12851.

[22] Romano A, Saino N, Møller AP. Viability and expression of sexual ornaments in the barn swallow Hirundo rustica: a meta-analysis. Journal of Evolutionary Biology. 2017;30(10):1929–1935. doi:10.1111/jeb.13151.

[23] Davidson KE, Fowler MS, Skov MW, Doerr SH, Beaumont N, Griffin JN. Livestock grazing alters multiple ecosystem properties and services in salt marshes: a meta-analysis. Journal of Applied Ecology. 2017;54(5):1395–1405. doi:10.1111/1365-2664.12892.

[24] Grant JWA, Weir LK, Steingrímsson SÓ. Territory size decreases minimally with increasing food abundance in stream salmonids: Implications for population regulation. Journal of Animal Ecology. 2017;86(6):1308–1316. doi:10.1111/1365-2656.12737.

[25] Horswill C, O'Brien SH, Robinson RA. Density dependence and marine bird populations: are wind farm assessments precautionary? Journal of Applied Ecology. 2017;54(5):1406–1414. doi:10.1111/1365-2664.12841.

[26] Auer SK, Killen SS, Rezende EL. Resting vs. active: a meta-analysis of the intra- and inter-specific associations between minimum, sustained, and maximum metabolic rates in vertebrates. Functional Ecology. 2017;31(9):1728–1738. doi:10.1111/1365-2435.12879.

[27] Buchanan AL, Hermann SL, Lund M, Szendrei Z. A meta-analysis of non-consumptive predator effects in arthropods: the influence of organismal and environmental characteristics. Oikos. 2017;126(9):1233–1240. doi:10.1111/oik.04384.

[28] Xu X, Medvigy D, Joseph Wright S, Kitajima K, Wu J, Albert LP, et al. Variations of leaf longevity in tropical moist forests predicted by a trait-driven carbon optimality model. Ecology Letters. 2017;20(9):1097–1106. doi:10.1111/ele.12804.

[29] Soria M, Leigh C, Datry T, Bini LM, Bonada N. Biodiversity in perennial and intermittent rivers: a meta-analysis. Oikos. 2017;126(8):1078–1089. doi:10.1111/oik.04118.

[30] Delavaux CS, Smith-Ramesh LM, Kuebbing SE. Beyond nutrients: a meta-analysis of the diverse effects of arbuscular mycorrhizal fungi on plants and soils. Ecology. 2017;98(8):2111–2119. doi:10.1002/ecy.1892.

[31] LaManna JA, Martin TE. Logging impacts on avian species richness and composition differ across latitudes and foraging and breeding habitat preferences. Biological Reviews. 2017;92(3):1657–1674. doi:10.1111/brv.12300.

[32] Leal LC, Peixoto PEC. Decreasing water availability across the globe improves the effectiveness of protective ant–plant mutualisms: a meta-analysis. Biological Reviews. 2017;92(3):1785–1794. doi:10.1111/brv.12307.

[33] Romano A, Costanzo A, Rubolini D, Saino N, Møller AP. Geographical and seasonal variation in the intensity of sexual selection in the barn swallow Hirundo rustica: a meta-analysis. Biological Reviews. 2017;92(3):1582–1600. doi:10.1111/brv.12297.

[34] Anthelme F, Meneses RI, Valero NNH, Pozo P, Dangles O. Fine nurse variations explain discrepancies in the stress-interaction relationship in alpine regions. Oikos. 2017;126(8):1173–1183. doi:10.1111/oik.04248.

[35] Hindrikson M, Remm J, Pilot M, Godinho R, Stronen AV, Baltrūnaité L, et al. Wolf population genetics in Europe: a systematic review, meta-analysis and suggestions for conservation and management. Biological Reviews. 2017;92(3):1601–1629. doi:10.1111/brv.12298.

[36] Knapp JL, Bartlett LJ, Osborne JL. Re-evaluating strategies for pollinator-dependent crops: How useful is parthenocarpy? Journal of Applied Ecology. 2017;54(4):1171–1179. doi:10.1111/1365-2664.12813.

[37] Gázquez A, Beemster GTS. What determines organ size differences between species? A meta-analysis of the cellular basis. New Phytologist. 2017;215(1):299–308. doi:10.1111/nph.14573.

[38] Hendershot JN, Read QD, Henning JA, Sanders NJ, Classen AT. Consistently inconsistent drivers of microbial diversity and abundance at macroecological scales. Ecology. 2017;98(7):1757–1763. doi:10.1002/ecy.1829.

[39] Farji-Brener AG, Werenkraut V. The effects of ant nests on soil fertility and plant performance: a meta-analysis. Journal of Animal Ecology. 2017;86(4):866–877. doi:10.1111/1365-2656.12672.

[40] Hitchcock DJ, Varpe Ø, Andersen T, Borgå K. Effects of reproductive strategies on pollutant concentrations in pinnipeds: a meta-analysis. Oikos. 2017;126(6):772–781. doi:10.1111/oik.03955.

[41] Miller SE, Barrueto M, Schluter D. A comparative analysis of experimental selection on the stickleback pelvis. Journal of Evolutionary Biology. 2017;30(6):1165–1176. doi:10.1111/jeb.13085.

[42] Rowiński PK, Rogell B. Environmental stress correlates with increases in both genetic and residual variances: A meta-analysis of animal studies. Evolution. 2017;71(5):1339–1351. doi:10.1111/evo.13201.

[43] Yue K, Fornara DA, Yang W, Peng Y, Peng C, Liu Z, et al. Influence of multiple global change drivers on terrestrial carbon storage: additive effects are common. Ecology Letters. 2017;20(5):663–672. doi:10.1111/ele.12767.

[44] Wood KA, Ponting J, D'Costa N, Newth JL, Rose PE, Glazov P, et al. Understanding intrinsic and extrinsic drivers of aggressive behaviour in waterbird assemblages: a meta-analysis. Animal Behaviour. 2017;126:209–216. doi:10.1016/j.anbehav.2017.02.008.

[45] Greenwood S, Ruiz-Benito P, Martínez-Vilalta J, Lloret F, Kitzberger T, Allen CD, et al. Tree mortality across biomes is promoted by drought intensity, lower wood density and higher specific leaf area. Ecology Letters. 2017;20(4):539–553. doi:10.1111/ele.12748.

[46] Martin PA, Newton AC, Bullock JM. Impacts of invasive plants on carbon pools depend on both species' traits and local climate. Ecology. 2017;98(4):1026–1035. doi:10.1002/ecy.1711.

[47] Holtmann B, Lagisz M, Nakagawa S. Metabolic rates, and not hormone levels, are a likely mediator of between-individual differences in behaviour: a meta-analysis. Functional Ecology. 2017;31(3):685–696. doi:10.1111/1365-2435.12779.

[48] Catano CP, Dickson TL, Myers JA. Dispersal and neutral sampling mediate contingent effects of disturbance on plant beta-diversity: a meta-analysis. Ecology Letters. 2017;20(3):347–356. doi:10.1111/ele.12733.

[49] Poulin R, Pérez-Ponce de León G. Global analysis reveals that cryptic diversity is linked with habitat but not mode of life. Journal of Evolutionary Biology. 2017;30(3):641–649. doi:10.1111/jeb.13034.

[50] Usui T, Butchart SHM, Phillimore AB. Temporal shifts and temperature sensitivity of avian spring migratory phenology: a phylogenetic meta-analysis. Journal of Animal Ecology. 2017;86(2):250–261. doi:10.1111/1365-2656.12612.

[51] Hrycik AR, Almeida LZ, Höök TO. Sub-lethal effects on fish provide insight into a biologically-relevant threshold of hypoxia. Oikos. 2017;126(3):307–317. doi:10.1111/oik.03678.

[52] Rossetti MR, Tscharntke T, Aguilar R, Batáry P. Responses of insect herbivores and herbivory to habitat fragmentation: a hierarchical meta-analysis. Ecology Letters. 2017;20(2):264–272. doi:10.1111/ele.12723.

[53] Sorenson GH, Dey CJ, Madliger CL, Love OP. Effectiveness of baseline corticosterone as a monitoring tool for fitness: a meta-analysis in seabirds. Oecologia. 2017;183(2):353–365. doi:10.1007/s00442-016-3774-3.

[54] Foo YZ, Nakagawa S, Rhodes G, Simmons LW. The effects of sex hormones on immune function: a meta-analysis. Biological Reviews. 2017;92(1):551–571. doi:10.1111/brv.12243.

[55] Hossie T, Landolt K, Murray DL. Determinants and co-expression of anti-predator responses in amphibian tadpoles: a meta-analysis. Oikos. 2017;126(2):n/a–n/a. doi:10.1111/oik.03305.

[56] Booksmythe I, Mautz B, Davis J, Nakagawa S, Jennions MD. Facultative adjustment of the offspring sex ratio and male attractiveness: a systematic review and meta-analysis. Biological Reviews. 2017;92(1):108–134. doi:10.1111/brv.12220.

[57] Ameye M, Allmann S, Verwaeren J, Smagghe G, Haesaert G, Schuurink RC, et al. Green leaf volatile production by plants: a meta-analysis. New Phytologist. 2017; p. n/a–n/a. doi:10.1111/nph.14671.

[58] Vidal MC, Murphy SM. Bottom-up vs. top-down effects on terrestrial insect herbivores: a meta-analysis. Ecology Letters. 2018;21(1):138–150. doi:10.1111/ele.12874.

[59] Deng Q, McMahon DE, Xiang Y, Yu CL, Jackson RB, Hui D. A global meta-analysis of soil phosphorus dynamics after afforestation. New Phytologist. 2017;213(1):181–192. doi:10.1111/nph.14119.

[60] Lee MR, Bernhardt ES, van Bodegom PM, Cornelissen JHC, Kattge J, Laughlin DC, et al. Invasive species' leaf traits and dissimilarity from natives shape their impact on nitrogen cycling: a meta-analysis. New Phytologist. 2017;213(1):128–139. doi:10.1111/nph.14115.

[61] Zvereva EL, Zverev V, Kruglova OY, Kozlov MV. Strategies of chemical anti-predator defences in leaf beetles: is sequestration of plant toxins less costly than de novo synthesis? Oecologia. 2017;183(1):93–106. doi:10.1007/s00442-016-3743-x.

[62] Xiao Z, Wang X, Koricheva J, Kergunteuil A, Le Bayon RC, Liu M, et al. Earthworms affect plant growth and resistance against herbivores: A meta-analysis. Functional Ecology. 2018;32(1):150–160. doi:10.1111/1365-2435.12969.

[63] Camenzind T, Hättenschwiler S, Treseder KK, Lehmann A, Rillig MC. Nutrient limitation of soil microbial processes in tropical forests. Ecological Monographs. 2018;88(1):4–21. doi:10.1002/ecm.1279.

[64] Yang H, Zhang Q, Koide RT, Hoeksema JD, Tang J, Bian X, et al. Taxonomic resolution is a determinant of biodiversity effects in arbuscular mycorrhizal fungal communities. Journal of Ecology. 2017;105(1):219–228. doi:10.1111/1365-2745.12655.

[65] Winternitz J, Abbate JL, Huchard E, Havlíček J, Garamszegi LZ. Patterns of MHC-dependent mate selection in humans and nonhuman primates: a meta-analysis. Molecular Ecology. 2017;26(2):668–688. doi:10.1111/mec.13920.

[66] Molnar JL, Diogo R, Hutchinson JR, Pierce SE. Reconstructing pectoral appendicular muscle anatomy in fossil fish and tetrapods over the fins-to-limbs transition. Biological Reviews. 2017; p. n/a–n/a. doi:10.1111/brv.12386.

[67] Grueber CE, Gray LJ, Morris KM, Simpson SJ, Senior AM. Intergenerational effects of nutrition on immunity: a systematic review and meta-analysis. Biological Reviews. 2018;93(2):1108–1124. doi:10.1111/brv.12387.

[68] Gruner DS, Bracken MES, Berger SA, Eriksson BK, Gamfeldt L, Matthiessen B, et al. Effects of experimental warming on biodiversity depend on ecosystem type and local species composition. Oikos. 2016;126(1):8–17. doi:10.1111/oik.03688.

[69] Dynarski KA, Houlton BZ. Nutrient limitation of terrestrial free-living nitrogen fixation. New Phytologist. 2018;217(3):1050–1061. doi:10.1111/nph.14905.

[70] Noble DWA, Stenhouse V, Schwanz LE. Developmental temperatures and phenotypic plasticity in reptiles: a systematic review and meta-analysis. Biological Reviews. 2018;93(1):72–97. doi:10.1111/brv.12333.

[71] Junker RR, Kuppler J, Amo L, Blande JD, Borges RM, van Dam NM, et al. Covariation and phenotypic integration in chemical communication displays: biosynthetic constraints and eco-evolutionary implications. New Phytologist. 2017; p. n/a–n/a. doi:10.1111/nph.14505.

[72] Risely A, Klaassen M, Hoye BJ. Migratory animals feel the cost of getting sick: A meta-analysis across species. Journal of Animal Ecology. 2017;87(1):301–314. doi:10.1111/1365-2656.12766.

[73] Ronget V, Gaillard J, Coulson T, Garratt M, Gueyffier F, Lega J, et al. Causes and consequences of variation in offspring body mass: meta-analyses in birds and mammals. Biological Reviews. 2017;93(1):1–27. doi:10.1111/brv.12329.

[74] Rohner PT, Teder T, Esperk T, Lüpold S, Blanckenhorn WU. The evolution of male-biased sexual size dimorphism is associated with increased body size plasticity in males. Functional Ecology. 2017;In press:n/a–n/a. doi:10.1111/1365-2435.13004.

[75] Goodell K, Parker IM. Invasion of a dominant floral resource: effects on the floral community and pollination of native plants. Ecology. 2017;98(1):57–69. doi:10.1002/ecy.1639.

[76] Sievers M, Hale R, Parris KM, Swearer SE. Impacts of human-induced environmental change in wetlands on aquatic animals. Biological Reviews. 2017;93(1):529–554. doi:10.1111/brv.12358.

[77] Harts AMF, Booksmythe I, Jennions MD. Mate guarding and frequent copulation in birds: A meta-analysis of their relationship to paternity and male phenotype. Evolution. 2016;70(12):2789–2808. doi:10.1111/evo.13081.

[78] Poorter H, Fiorani F, Pieruschka R, Wojciechowski T, Putten WH, Kleyer M, et al. Pampered inside, pestered outside? Differences and similarities between plants growing in controlled conditions and in the field. New Phytologist. 2016;212(4):838–855. doi:10.1111/nph.14243.

[79] Edge CB, Houlahan JE, Jackson DA, Fortin M. The response of amphibian larvae to environmental change is both consistent and variable. Oikos. 2016;125(12):1700–1711. doi:10.1111/oik.03166.

[80] Gill AL, Finzi AC. Belowground carbon flux links biogeochemical cycles and resource-use efficiency at the global scale. Ecology Letters. 2016;19(12):1419–1428. doi:10.1111/ele.12690.

[81] Buoro M, Olden JD, Cucherousset J. Global Salmonidae introductions reveal stronger ecological effects of changing intraspecific compared to interspecific diversity. Ecology Letters. 2016;19(11):1363–1371. doi:10.1111/ele.12673.

[82] Besson AA, Lagisz M, Senior AM, Hector KL, Nakagawa S. Effect of maternal diet on offspring coping styles in rodents: a systematic review and meta-analysis. Biological Reviews. 2016;91(4):1065–1080. doi:10.1111/brv.12210.

[83] Flick AJ, Acevedo MA, Elderd BD. The negative effects of pathogen-infected prey on predators: a meta-analysis. Oikos. 2016;125(11):1554–1560. doi:10.1111/oik.03458.

[84] Jaffé R, Pope N, Acosta AL, Alves DA, Arias MC, De la Rúa P, et al. Beekeeping practices and geographic distance, not land use, drive gene flow across tropical bees. Molecular Ecology. 2016;25(21):5345–5358. doi:10.1111/mec.13852.

[85] Tsen EWJ, Sitzia T, Webber BL. To core, or not to core: the impact of coring on tree health and a best-practice framework for collecting dendrochronological information from living trees. Biological Reviews. 2016;91(4):899–924. doi:10.1111/brv.12200.

[86] Derroire G, Balvanera P, Castellanos-Castro C, Decocq G, Kennard DK, Lebrija-Trejos E, et al. Resilience of tropical dry forests – a meta-analysis of changes in species diversity and composition during secondary succession. Oikos. 2016;125(10):1386–1397. doi:10.1111/oik.03229.

[87] Lemoine NP, Hoffman A, Felton AJ, Baur L, Chaves F, Gray J, et al. Underappreciated problems of low replication in ecological field studies. Ecology. 2016;97(10):2554–2561. doi:10.1002/ecy.1506.

[88] Xu X, Medvigy D, Powers JS, Becknell JM, Guan K. Diversity in plant hydraulic traits explains seasonal and inter-annual variations of vegetation dynamics in seasonally dry tropical forests. New Phytologist. 2016;212(1):80–95. doi:10.1111/nph.14009.

[89] Hidding B, Bakker ES, Hootsmans MJM, Hilt S. Synergy between shading and herbivory triggers macrophyte loss and regime shifts in aquatic systems. Oikos. 2016;125(10):1489–1495. doi:10.1111/oik.03104.

[90] Turney S, Buddle CM. Pyramids of species richness: the determinants and distribution of species diversity across trophic levels. Oikos. 2016;125(9):1224–1232. doi:10.1111/oik.03404.

[91] Gibert A, Gray EF, Westoby M, Wright IJ, Falster DS. On the link between functional traits and growth rate: meta-analysis shows effects change with plant size, as predicted. Journal of Ecology. 2016;104(5):1488–1503. doi:10.1111/1365-2745.12594.

[92] Jessop TS, Lane ML, Teasdale L, Stuart-Fox D, Wilson RS, Careau V, et al. Multiscale Evaluation of Thermal Dependence in the Glucocorticoid Response of Vertebrates. The American Naturalist. 2016;188(3):342–356. doi:10.1086/687588.

[93] Katz DSW. The effects of invertebrate herbivores on plant population growth: a meta-regression analysis. Oecologia. 2016;182(1):43–53. doi:10.1007/s00442-016-3602-9.

[94] Fuzessy LF, Cornelissen TG, Janson C, Silveira FAO. How do primates affect seed germination? A meta-analysis of gut passage effects on neotropical plants. Oikos. 2016;125(8):1069–1080. doi:10.1111/oik.02986.

[95] Cooke J, Leishman MR. Consistent alleviation of abiotic stress with silicon addition: a meta-analysis. Functional Ecology. 2016;30(8):1340–1357. doi:10.1111/1365-2435.12713.

[96] Yoon S, Read Q. Consequences of exotic host use: impacts on Lepidoptera and a test of the ecological trap hypothesis. Oecologia. 2016;181(4):985–996. doi:10.1007/s00442-016-3560-2.

[97] Coetzee BWT, Chown SL. A meta-analysis of human disturbance impacts on Antarctic wildlife. Biological Reviews. 2016;91(3):578–596. doi:10.1111/brv.12184.

[98] Östman Ö, Eklöf J, Eriksson BK, Olsson J, Moksnes PO, Bergström U. Top-down control as important as nutrient enrichment for eutrophication effects in North Atlantic coastal ecosystems. Journal of Applied Ecology. 2016;53(4):1138–1147. doi:10.1111/1365-2664.12654.

[99] He Q, Silliman BR. Consumer control as a common driver of coastal vegetation worldwide. Ecological Monographs. 2016;86(3):278–294. doi:10.1002/ecm.1221.

[100] Delgado-Baquerizo M, Maestre FT, Reich PB, Trivedi P, Osanai Y, Liu YR, et al. Carbon content and climate variability drive global soil bacterial diversity patterns. Ecological Monographs. 2016;86(3):373–390. doi:10.1002/ecm.1216.

[101] Thom D, Seidl R. Natural disturbance impacts on ecosystem services and biodiversity in temperate and boreal forests. Biological Reviews. 2016;91(3):760–781. doi:10.1111/brv.12193.

[102] Liu J, Wu N, Wang H, Sun J, Peng B, Jiang P, et al. Nitrogen addition affects chemical compositions of plant tissues, litter and soil organic matter. Ecology. 2016;97(7):1796–1806. doi:10.1890/15-1683.1.

[103] Liao H, D'Antonio CM, Chen B, Huang Q, Peng S. How much do phenotypic plasticity and local genetic variation contribute to phenotypic divergences along environmental gradients in widespread invasive plants? A meta-analysis. Oikos. 2016;125(7):905–917. doi:10.1111/oik.02372.

[104] Arceo-Gómez G, Ashman TL. Invasion status and phylogenetic relatedness predict cost of heterospecific pollen receipt: implications for native biodiversity decline. Journal of Ecology. 2016;104(4):1003–1008. doi:10.1111/1365-2745.12586.

[105] Oduor AMO, Leimu R, Kleunen Mv. Invasive plant species are locally adapted just as frequently and at least as strongly as native plant species. Journal of Ecology. 2016;104(4):957–968. doi:10.1111/1365-2745.12578.

[106] Ficetola GF, Maiorano L. Contrasting effects of temperature and precipitation change on amphibian phenology, abundance and performance. Oecologia. 2016;181(3):683–693. doi:10.1007/s00442-016-3610-9.

[107] Kriengwatana B, Spierings MJ, ten Cate C. Auditory discrimination learning in zebra finches: effects of sex, early life conditions and stimulus characteristics. Animal Behaviour. 2016;116:99–112. doi:10.1016/j.anbehav.2016.03.028.

[108] Samia DSM, Blumstein DT, Stankowich T, Cooper WE. Fifty years of chasing lizards: new insights advance optimal escape theory. Biological Reviews. 2016;91(2):349–366. doi:10.1111/brv.12173.

[109] Smilanich AM, Fincher RM, Dyer LA. Does plant apparency matter? Thirty years of data provide limited support but reveal clear patterns of the effects of plant chemistry on herbivores. New Phytologist. 2016;210(3):1044–1057. doi:10.1111/nph.13875.

[110] Boyle WA, Sandercock BK, Martin K. Patterns and drivers of intraspecific variation in avian life history along elevational gradients: a meta-analysis. Biological Reviews. 2016;91(2):469–482. doi:10.1111/brv.12180.

[111] Blount JD, Vitikainen EIK, Stott I, Cant MA. Oxidative shielding and the cost of reproduction. Biological Reviews. 2016;91(2):483–497. doi:10.1111/brv.12179.

[112] Wesner JS. Contrasting effects of fish predation on benthic versus emerging prey: a meta-analysis. Oecologia. 2016;180(4):1205–1211. doi:10.1007/s00442-015-3539-4.

[113] Moore MP, Riesch R, Martin RA. The predictability and magnitude of life-history divergence to ecological agents of selection: a meta-analysis in livebearing fishes. Ecology Letters. 2016;19(4):435–442. doi:10.1111/ele.12576.

[114] Rowen E, Kaplan I. Eco-evolutionary factors drive induced plant volatiles: a meta-analysis. New Phytologist. 2016;210(1):284–294. doi:10.1111/nph.13804.
[115] Thurman TJ, Barrett RDH. The genetic consequences of selection in natural populations. Molecular Ecology. 2016;25(7):1429–1448. doi:10.1111/mec.13559.
[116] Hébert MP, Beisner BE, Maranger R. A meta-analysis of zooplankton functional traits influencing ecosystem function. Ecology. 2016;97(4):1069–1080. doi:10.1890/15-1084.1.
[117] Anderson JT. Plant fitness in a rapidly changing world. New Phytologist. 2016;210(1):81–87. doi:10.1111/nph.13693.
[118] Lorenz S, Martinez-Fernández V, Alonso C, Mosselman E, García de Jalón D, González del Tánago M, et al. Fuzzy cognitive mapping for predicting hydromorphological responses to multiple pressures in rivers. Journal of Applied Ecology. 2016;53(2):559–566. doi:10.1111/1365-2664.12569.
[119] Dougherty LR, Shuker DM. Variation in pre- and post-copulatory sexual selection on male genital size in two species of lygaeid bug. Behavioral Ecology and Sociobiology. 2016;70(4):625–637. doi:10.1007/s00265-016-2082-6.
[120] Crouzeilles R, Curran M. Which landscape size best predicts the influence of forest cover on restoration success? A global meta-analysis on the scale of effect. Journal of Applied Ecology. 2016;53(2):440–448. doi:10.1111/1365-2664.12590.
[121] van Katwijk MM, Thorhaug A, Marbà N, Orth RJ, Duarte CM, Kendrick GA, et al. Global analysis of seagrass restoration: the importance of large-scale planting. Journal of Applied Ecology. 2016;53(2):567–578. doi:10.1111/1365-2664.12562.
[122] McCary MA, Mores R, Farfan MA, Wise DH. Invasive plants have different effects on trophic structure of green and brown food webs in terrestrial ecosystems: a meta-analysis. Ecology Letters. 2016;19(3):328–335. doi:10.1111/ele.12562.
[123] Mazé-Guilmo E, Blanchet S, McCoy KD, Loot G. Host dispersal as the driver of parasite genetic structure: a paradigm lost? Ecology Letters. 2016;19(3):336–347. doi:10.1111/ele.12564.
[124] Allen DC, Wesner JS. Synthesis: comparing effects of resource and consumer fluxes into recipient food webs using meta-analysis. Ecology. 2016;97(3):594–604. doi:10.1890/15-1109.1.
[125] Zvereva EL, Kozlov MV. The costs and effectiveness of chemical defenses in herbivorous insects: a meta-analysis. Ecological Monographs. 2016;86(1):107–124. doi:10.1890/15-0911.1.
[126] Barton KE. Tougher and thornier: general patterns in the induction of physical defence traits. Functional Ecology. 2016;30(2):181–187. doi:10.1111/1365-2435.12495.
[127] Street SE, Cross CP, Brown GR. Exaggerated sexual swellings in female nonhuman primates are reliable signals of female fertility and body condition. Animal Behaviour. 2016;112(Supplement C):203–212. doi:10.1016/j.anbehav.2015.11.023.
[128] Moore FR, Shuker DM, Dougherty L. Stress and sexual signaling: a systematic review and meta-analysis. Behavioral Ecology. 2016;27(2):363–371. doi:10.1093/beheco/arv195.
[129] Roca IT, Desrochers L, Giacomazzo M, Bertolo A, Bolduc P, Deschesnes R, et al. Shifting song frequencies in response to anthropogenic noise: a meta-analysis on birds and anurans. Behavioral Ecology. 2016;27(5):1269–1274. doi:10.1093/beheco/arw060.
[130] Holman L. Bet hedging via multiple mating: A meta-analysis. Evolution. 2016;70(1):62–71. doi:10.1111/evo.12822.
[131] Vico G, Manzoni S, Nkurunziza L, Murphy K, Weih M. Trade-offs between seed output and life span – a quantitative comparison of traits between annual and perennial congeneric species. New Phytologist. 2016;209(1):104–114. doi:10.1111/nph.13574.
[132] Daskin JH, Pringle RM. Does primary productivity modulate the indirect effects of large herbivores? A global meta-analysis. Journal of Animal Ecology. 2016;85(4):857–868. doi:10.1111/1365-2656.12522.
[133] Gunton RM, Pöyry J. Scale-specific spatial density dependence in parasitoids: a multi-factor meta-analysis. Functional Ecology. 2016;30(9):1501–1510. doi:10.1111/1365-2435.12627.
[134] German RN, Thompson CE, Benton TG. Relationships among multiple aspects of agriculture's environmental impact and productivity: a meta-analysis to guide sustainable agriculture. Biological Reviews. 2017;92(2):716–738. doi:10.1111/brv.12251.
[135] Shantz AA, Lemoine NP, Burkepile DE. Nutrient loading alters the performance of key nutrient exchange mutualisms. Ecology Letters. 2016;19(1):20–28. doi:10.1111/ele.12538.
[136] Dillingham PW, Moore JE, Fletcher D, Cortés E, Curtis KA, James KC, et al. Improved estimation of intrinsic growth rmax for long-lived species: integrating matrix models and allometry. Ecological Applications. 2016;26(1):322–333. doi:10.1890/14-1990.
[137] Strader ME, Aglyamova GV, Matz MV. Red fluorescence in coral larvae is associated with a diapause-like state. Molecular Ecology. 2016;25(2):559–569. doi:10.1111/mec.13488.
[138] Wood KA, O'Hare MT, McDonald C, Searle KR, Daunt F, Stillman RA. Herbivore regulation of plant abundance in aquatic ecosystems. Biological Reviews. 2017;92(2):1128–1141. doi:10.1111/brv.12272.
[139] Deng Q, Hui D, Luo Y, Elser J, Wang YP, Loladze I, et al. Down-regulation of tissue N:P ratios in terrestrial plants by elevated CO2. Ecology. 2015;96(12):3354–3362. doi:10.1890/15-0217.1.
[140] Garamszegi LZ, Markó G, Szász E, Zsebők S, Azcárate M, Herczeg G, et al. Among-year variation in the repeatability, within- and between-individual, and phenotypic correlations of behaviors in a natural population. Behavioral Ecology and Sociobiology. 2015;69(12):2005–2017. doi:10.1007/s00265-015-2012-z.
[141] Lüpold S, Simmons LW, Tomkins JL, Fitzpatrick JL. No evidence for a trade-off between sperm length and male premating weaponry. Journal of Evolutionary Biology. 2015;28(12):2187–2195. doi:10.1111/jeb.12742.
[142] Moreno-Mateos D, Meli P, Vara-Rodríguez MI, Aronson J. Ecosystem response to interventions: lessons from restored and created wetland ecosystems. Journal of Applied Ecology. 2015;52(6):1528–1537. doi:10.1111/1365-2664.12518.

[143] Katano I, Doi H, Eriksson BK, Hillebrand H. A cross-system meta-analysis reveals coupled predation effects on prey biomass and diversity. Oikos. 2015;124(11):1427–1435. doi:10.1111/oik.02430.

[144] Graham S, Chapuis E, Meconcelli S, Bonel N, Sartori K, Christophe A, et al. Size-assortative mating in simultaneous hermaphrodites: an experimental test and a meta-analysis. Behavioral Ecology and Sociobiology. 2015;69(11):1867–1878. doi:10.1007/s00265-015-1999-5.

[145] Goessling JM, Kennedy H, Mendonça MT, Wilson AE. A meta-analysis of plasma corticosterone and heterophil : lymphocyte ratios – is there conservation of physiological stress responses over time? Functional Ecology. 2015;29(9):1189–1196. doi:10.1111/1365-2435.12442.

[146] Romero GQ, Gonçalves-Souza T, Vieira C, Koricheva J. Ecosystem engineering effects on species diversity across ecosystems: a meta-analysis. Biological Reviews. 2015;90(3):877–890. doi:10.1111/brv.12138.

[147] Nielsen JM, Popp BN, Winder M. Meta-analysis of amino acid stable nitrogen isotope ratios for estimating trophic position in marine organisms. Oecologia. 2015;178(3):631–642. doi:10.1007/s00442-015-3305-7.

[148] James J, Slater FM, Vaughan IP, Young KA, Cable J. Comparing the ecological impacts of native and invasive crayfish: could native species' translocation do more harm than good? Oecologia. 2015;178(1):309–316. doi:10.1007/s00442-014-3195-0.

[149] Esteban R, Barrutia O, Artetxe U, Fernández-Marín B, Hernández A, García-Plazaola JI. Internal and external factors affecting photosynthetic pigment composition in plants: a meta-analytical approach. New Phytologist. 2015;206(1):268–280. doi:10.1111/nph.13186.

[150] Becker DJ, Streicker DG, Altizer S. Linking anthropogenic resources to wildlife–pathogen dynamics: a review and meta-analysis. Ecology Letters. 2015;18(5):483–495. doi:10.1111/ele.12428.

[151] Starko S, Claman BZ, Martone PT. Biomechanical consequences of branching in flexible wave-swept macroalgae. New Phytologist. 2015;206(1):133–140. doi:10.1111/nph.13182.

[152] Hsu YH, Schroeder J, Winney I, Burke T, Nakagawa S. Are extra-pair males different from cuckolded males? A case study and a meta-analytic examination. Molecular Ecology. 2015;24(7):1558–1571. doi:10.1111/mec.13124.

[153] Mazza CA, Ballaré CL. Photoreceptors UVR8 and phytochrome B cooperate to optimize plant growth and defense in patchy canopies. New Phytologist. 2015;207(1):4–9. doi:10.1111/nph.13332.

[154] Jackson MC. Interactions among multiple invasive animals. Ecology. 2015;96(8):2035–2041. doi:10.1890/15-0171.1.

[155] Arct A, Drobniak SM, Cichoń M. Genetic similarity between mates predicts extrapair paternity—a meta-analysis of bird studies. Behavioral Ecology. 2015;26(4):959–968. doi:10.1093/beheco/arv004.

[156] Boudreau SA, Anderson SC, Worm B. Top-down and bottom-up forces interact at thermal range extremes on American lobster. Journal of Animal Ecology. 2015;84(3):840–850. doi:10.1111/1365-2656.12322.

[157] Yang LY, Machado CA, Dang XD, Peng YQ, Yang DR, Zhang DY, et al. The incidence and pattern of copollinator diversification in dioecious and monoecious figs. Evolution. 2015;69(2):294–304. doi:10.1111/evo.12584.

[158] Siefert A, Violle C, Chalmandrier L, Albert CH, Taudiere A, Fajardo A, et al. A global meta-analysis of the relative extent of intraspecific trait variation in plant communities. Ecology Letters. 2015;18(12):1406–1419. doi:10.1111/ele.12508.

[159] Beninde J, Veith M, Hochkirch A. Biodiversity in cities needs space: a meta-analysis of factors determining intra-urban biodiversity variation. Ecology Letters. 2015;18(6):581–592. doi:10.1111/ele.12427.

[160] Senior AM, Nakagawa S, Lihoreau M, Simpson SJ, Raubenheimer D. An Overlooked Consequence of Dietary Mixing: A Varied Diet Reduces Interindividual Variance in Fitness. The American Naturalist. 2015;186(5):649–659. doi:10.1086/683182.

[161] Albertson LK, Allen DC. Meta-analysis: abundance, behavior, and hydraulic energy shape biotic effects on sediment transport in streams. Ecology. 2015;96(5):1329–1339. doi:10.1890/13-2138.1.

[162] Frankham R. Genetic rescue of small inbred populations: meta-analysis reveals large and consistent benefits of gene flow. Molecular Ecology. 2015;24(11):2610–2618. doi:10.1111/mec.13139.

[163] Voje KL. Scaling of Morphological Characters across Trait Type, Sex, and Environment: A Meta-analysis of Static Allometries. The American Naturalist. 2015;187(1):89–98. doi:10.1086/684159.

[164] Slot M, Kitajima K. General patterns of acclimation of leaf respiration to elevated temperatures across biomes and plant types. Oecologia. 2015;177(3):885–900. doi:10.1007/s00442-014-3159-4.

[165] Jahnke M, Olsen JL, Procaccini G. A meta-analysis reveals a positive correlation between genetic diversity metrics and environmental status in the long-lived seagrass Posidonia oceanica. Molecular Ecology. 2015;24(10):2336–2348. doi:10.1111/mec.13174.

[166] Iacarella JC, Dick JTA, Alexander ME, Ricciardi A. Ecological impacts of invasive alien species along temperature gradients: testing the role of environmental matching. Ecological Applications. 2015;25(3):706–716. doi:10.1890/14-0545.1.

[167] Quesnelle PE, Lindsay KE, Fahrig L. Relative effects of landscape-scale wetland amount and landscape matrix quality on wetland vertebrates: a meta-analysis. Ecological Applications. 2015;25(3):812–825. doi:10.1890/14-0362.1.

[168] Dougherty LR, Shuker DM. The effect of experimental design on the measurement of mate choice: a meta-analysis. Behavioral Ecology. 2015;26(2):311–319. doi:10.1093/beheco/aru125.

[169] Paz-Vinas I, Loot G, Stevens VM, Blanchet S. Evolutionary processes driving spatial patterns of intraspecific genetic diversity in river ecosystems. Molecular Ecology. 2015;24(18):4586–4604. doi:10.1111/mec.13345.

[170] Sistla SA, Appling AP, Lewandowska AM, Taylor BN, Wolf AA. Stoichiometric flexibility in response to fertilization along gradients of environmental and organismal nutrient richness. Oikos. 2015;124(7):949–959. doi:10.1111/oik.02385.

[171] Jauni M, Gripenberg S, Ramula S. Non-native plant species benefit from disturbance: a meta-analysis. Oikos. 2015;124(2):122–129. doi:10.1111/oik.01416.

[172] Bunn RA, Ramsey PW, Lekberg Y. Do native and invasive plants differ in their interactions with arbuscular mycorrhizal fungi? A meta-analysis. Journal of Ecology. 2015;103(6):1547–1556. doi:10.1111/1365-2745.12456.

[173] Mehrabi Z, Tuck SL. Relatedness is a poor predictor of negative plant–soil feedbacks. New Phytologist. 2015;205(3):1071–1075. doi:10.1111/nph.13238.

[174] Wang X, Taub DR, Jablonski LM. Reproductive allocation in plants as affected by elevated carbon dioxide and other environmental changes: a synthesis using meta-analysis and graphical vector analysis. Oecologia. 2015;177(4):1075–1087. doi:10.1007/s00442-014-3191-4.

[175] Albert A, Auffret AG, Cosyns E, Cousins SAO, D'hondt B, Eichberg C, et al. Seed dispersal by ungulates as an ecological filter: a trait-based meta-analysis. Oikos. 2015;124(9):1109–1120. doi:10.1111/oik.02512.

[176] Mijangos JL, Pacioni C, Spencer PBS, Craig MD. Contribution of genetics to ecological restoration. Molecular Ecology. 2015;24(1):22–37. doi:10.1111/mec.12995.

[177] Tamburello L, Maggi E, Benedetti-Cecchi L, Bellistri G, Rattray AJ, Ravaglioli C, et al. Variation in the impact of non-native seaweeds along gradients of habitat degradation: a meta-analysis and an experimental test. Oikos. 2015;124(9):1121–1131. doi:10.1111/oik.02197.

[178] Ferreira V, Castagneyrol B, Koricheva J, Gulis V, Chauvet E, Graça MAS. A meta-analysis of the effects of nutrient enrichment on litter decomposition in streams. Biological Reviews. 2015;90(3):669–688. doi:10.1111/brv.12125.

[179] Stephens AEA, Westoby M. Effects of insect attack to stems on plant survival, growth, reproduction and photosynthesis. Oikos. 2015;124(3):266–273. doi:10.1111/oik.01809.

[180] Anderegg WRL. Spatial and temporal variation in plant hydraulic traits and their relevance for climate change impacts on vegetation. New Phytologist. 2015;205(3):1008–1014. doi:10.1111/nph.12907.

[181] Yuan ZY, Chen HYH. Negative effects of fertilization on plant nutrient resorption. Ecology. 2015;96(2):373–380. doi:10.1890/14-0140.1.

[182] Valls A, Coll M, Christensen V. Keystone species: toward an operational concept for marine biodiversity conservation. Ecological Monographs. 2015;85(1):29–47. doi:10.1890/14-0306.1.

[183] Colautti RI, Lau JA. Contemporary evolution during invasion: evidence for differentiation, natural selection, and local adaptation. Molecular Ecology. 2015;24(9):1999–2017. doi:10.1111/mec.13162.

[184] Fuiman LA, Connelly TL, Lowerre-Barbieri SK, McClelland JW. Egg boons: central components of marine fatty acid food webs. Ecology. 2015;96(2):362–372. doi:10.1890/14-0571.1.

[185] Baeten L, Davies TJ, Verheyen K, Calster HV, Vellend M. Disentangling dispersal from phylogeny in the colonization capacity of forest understorey plants. Journal of Ecology. 2015;103(1):175–183. doi:10.1111/1365-2745.12333.

[186] Taranu ZE, Gregory-Eaves I, Leavitt PR, Bunting L, Buchaca T, Catalan J, et al. Acceleration of cyanobacterial dominance in north temperate-subarctic lakes during the Anthropocene. Ecology Letters. 2015;18(4):375–384. doi:10.1111/ele.12420.

[187] Pintor LM, Byers JE. Do native predators benefit from non-native prey? Ecology Letters. 2015;18(11):1174–1180. doi:10.1111/ele.12496.

[188] Harper KA, Macdonald SE, Mayerhofer MS, Biswas SR, Esseen PA, Hylander K, et al. Edge influence on vegetation at natural and anthropogenic edges of boreal forests in Canada and Fennoscandia. Journal of Ecology. 2015;103(3):550–562. doi:10.1111/1365-2745.12398.

[189] Culina A, Radersma R, Sheldon BC. Trading up: the fitness consequences of divorce in monogamous birds. Biological Reviews. 2015;90(4):1015–1034. doi:10.1111/brv.12143.

[190] Bracken MES, Hillebrand H, Borer ET, Seabloom EW, Cebrian J, Cleland EE, et al. Signatures of nutrient limitation and co-limitation: responses of autotroph internal nutrient concentrations to nitrogen and phosphorus additions. Oikos. 2015;124(2):113–121. doi:10.1111/oik.01215.

[191] Périquet S, Fritz H, Revilla E. The Lion King and the Hyaena Queen: large carnivore interactions and coexistence. Biological Reviews. 2015;90(4):1197–1214. doi:10.1111/brv.12152.

[192] Elliott KH, Hare JF, Vaillant ML, Gaston AJ, Ropert-Coudert Y, Anderson WG. Ageing gracefully: physiology but not behaviour declines with age in a diving seabird. Functional Ecology. 2015;29(2):219–228. doi:10.1111/1365-2435.12316.

[193] Lafuente A, Pérez-Palacios P, Doukkali B, Molina-Sánchez MD, Jiménez-Zurdo JI, Caviedes MA, et al. Unraveling the effect of arsenic on the model Medicago–Ensifer interaction: a transcriptomic meta-analysis. New Phytologist. 2015;205(1):255–272. doi:10.1111/nph.13009.

[194] Gamfeldt L, Lefcheck JS, Byrnes JEK, Cardinale BJ, Duffy JE, Griffin JN. Marine biodiversity and ecosystem functioning: what's known and what's next? Oikos. 2015;124(3):252–265. doi:10.1111/oik.01549.

[195] Dryad. Joint Data Archiving Policy (JDAP); 2020. Available from: `https://datadryad.org/docs/JointDataArchivingPolicy.pdf`.

[196] Dryad. Dryad; 2021. Available from: `https://datadryad.org/stash/`.

[197] Rausher MD, McPeek MA, Moore AJ, Rieseberg L, Whitlock MC. Data Archiving. Evolution. 2010;64(3):603–604. doi:10.1111/j.1558-5646.2009.00940.x.

[198] Moore AJ, Mcpeek MA, Rausher MD, Rieseberg L, Whitlock MC. The need for archiving data in evolutionary biology. Journal of Evolutionary Biology. 2010;23(4):659–660. doi:10.1111/j.1420-9101.2010.01937.x.

[199] Rieseberg L, Vines T, Kane N. Editorial and retrospective 2010. Molecular Ecology. 2010;19(1):1–22. doi:10.1111/j.1365-294X.2009.04450.x.

[200] Whitlock M, McPeek M, Rausher M, Rieseberg L, Moore A. Data Archiving. The American Naturalist. 2010;175(2):145–146. doi:10.1086/650340.

[201] Fox CW, Irschick DJ, Knapp AK, Thompson K, Baker L, Meyer J. Functional ecology: moving forward into a new era of publishing. Functional Ecology. 2014;28(2):291–292. doi:10.1111/1365-2435.12254.

[202] Sandhu L, Baker L. Journal of Ecology is part of new BES data archiving policy; 2014. Available from: `https://jecologyblog.com/2014/01/20/journal-of-ecology-is-part-of-new-bes-data-archiving-policy/`.

[203] Caetano DS, Aisenberg A. Forgotten treasures: the fate of data in animal behaviour studies. Animal Behaviour. 2014;98:1–5. doi:10.1016/j.anbehav.2014.09.025.

[204] John Wiley & Sons I. Author Compliance Tool; 2021. Available from: `https://authorservices.wiley.com/author-resources/Journal-Authors/open-access/author-compliance-tool.html`.

[205] Magee AF, May MR, Moore BR. The Dawn of Open Access to Phylogenetic Data. PLOS ONE. 2014;9(10):e110268. doi:10.1371/journal.pone.0110268.

[206] Culina A, Berg Ivd, Evans S, Sánchez-Tójar A. Low availability of code in ecology: A call for urgent action. PLOS Biology. 2020;18(7):e3000763. doi:10.1371/journal.pbio.3000763.

[207] Borenstein M, Hedges L, Higgins J, Rothstein H. Comprehensive Meta-Analysis; 2013. Available from: `https://www.meta-analysis.com`.

[208] Rosenberg MS, Adams DC, Gurevitch J. Metawin: Statistical software for meta-analysis with resampling tests. Sunderland, MA: Sinauer Associates; 1997.

[209] Viechtbauer W. Conducting Meta-Analyses in R with the metafor Package. Journal of Statistical Software. 2010;36(1):1–48. doi:10.18637/jss.v036.i03.

[210] Hadfield JD. MCMC Methods for Multi-Response Generalized Linear Mixed Models: The MCMCglmm R Package. Journal of Statistical Software. 2010;33(1):1–22. doi:10.18637/jss.v033.i02.

[211] Knuth DE. Literate Programming. The Computer Journal. 1984;27(2):97–111. doi:10.1093/comjnl/27.2.97.

[212] Claerbout JF, Karrenbach M. Electronic documents give reproducible research a new meaning. In: SEG Technical Program Expanded Abstracts 1992. Society of Exploration Geophysicists; 1992. p. 601–604.

[213] Buckheit JB, Donoho DL. WaveLab and Reproducible Research. In: Antoniadis A, Oppenheim G, editors. Wavelets and Statistics. New York, NY: Springer; 1995. p. 55–81. Available from: `https://doi.org/10.1007/978-1-4612-2544-7_5`.

[214] Gentleman R, Temple Lang D. Statistical Analyses and Reproducible Research. Journal of Computational and Graphical Statistics. 2007;16(1):1–23. doi:10.1198/106186007X178663.

[215] R Core Team. R: A Language and Environment for Statistical Computing; 2022. Available from: `https://www.R-project.org/`.

[216] Hardwicke TE, Bohn M, MacDonald K, Hembacher E, Nuijten MB, Peloquin BN, et al. Analytic reproducibility in articles receiving open data badges at the journal Psychological Science: an observational study. Royal Society Open Science. 2021;8(1):201494. doi:10.1098/rsos.201494.

[217] Boettiger C. An introduction to Docker for reproducible research. ACM SIGOPS Operating Systems Review. 2015;49(1):71–79. doi:10.1145/2723872.2723882.

[218] Nüst D, Sochat V, Marwick B, Eglen SJ, Head T, Hirst T, et al. Ten simple rules for writing Dockerfiles for reproducible data science. PLOS Computational Biology. 2020;16(11):e1008316. doi:10.1371/journal.pcbi.1008316.

[219] Boettiger C, Eddelbuettel D. An Introduction to Rocker: Docker Containers for R. The R Journal. 2017;9(2):527–536.

[220] Marwick B, Boettiger C, Mullen L. Packaging Data Analytical Work Reproducibly Using R (and Friends). The American Statistician. 2018;72(1):80–88. doi:10.1080/00031305.2017.1375986.

[221] Medvigy D, Wofsy SC, Munger JW, Hollinger DY, Moorcroft PR. Mechanistic scaling of ecosystem function and dynamics in space and time: Ecosystem Demography model version 2. Journal of Geophysical Research: Biogeosciences. 2009;114(G1). doi:10.1029/2008JG000812.

[222] Rödel HG, Bora A, Kaetzke P, Khaschei M, Hutzelmeyer H, von Holst D. Over-winter survival in subadult European rabbits: weather effects, density dependence, and the impact of individual characteristics. Oecologia. 2004;140(4):566–576. doi:10.1007/s00442-004-1616-1.

[223] Barber-Meyer SM, Mech LD, White PJ. Elk Calf Survival and Mortality Following Wolf Restoration to Yellowstone National Park. Wildlife Monographs. 2008;169(1):1–30. doi:10.2193/2008-004.

[224] National Academies of Sciences, Engineering, and Medicine. Reproducibility and Replicability in Science. Washington, DC: The National Academies Press; 2019.
