# Supplementary material for: Computationally reproducing results from meta-analyses in ecology and evolutionary biology using shared code and data
Source: PLoS One. 2024 Mar 13;19(3):e0300333. doi: 10.1371/journal.pone.0300333 (PMC10936784; doi:10.1371/journal.pone.0300333)
Supplement: Attachment — Submitted filename: comments_PONE-D-23-35924.docx [file pone.0300333.s002.docx]

This paper touched upon an interesting topic about reproducibility of ecological and evolutionary meta-analysis papers. The authors found that the code and data sharing in ecological and evolutionary meta-analyses were alarmingly low (26/177; 15%), and only 4-13% of papers were successfully reproduced. Generally, I like the idea of this paper and it is a trendy paper that will arise discussion once formally released. Note that this paper has many merits that I would not mention emphasize them. I will focus on the concerns and the ways that this paper can be improved.

Major comments:

1. The conclusions made are a bit arbitrary and not in a rigorous way to me. They way they calculated the success rate was quite unusual. The core conclusion they made is “The low overall success rate was primarily driven by the low rate of code sharing”. This is a conclusion that any one can make, and does not need any research and analyses. How the author simple equate “no data and code sharing” and “not reproducible ”. A proper way is to use the subset that has data and code available (in this case, 26 papers) to calculate success rate. I assume this paper will reach a quite wide range of audiences and “4-13%” computational reproducibility rate is really conveying misleading information to the community. Literally, it means at best, only 13% meta-analyses can be reproduced in using some software like R; at worst, only 4% can be reproduced. Do the authors believe this?

2. The authors appear to “hacked” the abstract. The authors write in the abstract in a way with the intension to express “how bad of the data sharing and reproducibility is in the field of ecology and evolution”. A decent data sharing rate was found by the authors, as described as “sharing rate among this sample of meta-analysis articles was 75% (133 out of 177).”. This key finding should be emphasized in the abstract. The author said in the abstract: 26 articles (15%) were found to have obtainable data and code files. This sentence gives audience an illusion that only 15% meta-analyses shared data and code. But actually, only 15% meta-analyses shared both data and code at the same time. This is not a transparent way to report your results! Please make it clear.

3. The authors reproduced only meta-analyses with both data and code available, and calculated the success rate. This is not a proper way. What the authors should do is to reproduce the results of meta-analyses with data available, if following the definition of computational reproducibility. Having the original code available is not a criterion to filter your data and use this subset to calculate success rate.

Minor concerns:

Abstract:

The last sentence does not make sense. Please conclude this paper in a broader sense and discuss implications of the results the authors found.

Introduction:

The introduction was not well framed. The authors lacked the descriptions of the recent research progress of meta-science in ecology. The current version only slightly touched upon or simply mentioned meta-science in ecology and evolution. There are quite a few recent work that has not been properly credited. For example

Kimmel, K., Avolio, M. L. & Ferraro, P. J. Empirical evidence of widespread exaggeration bias and selective reporting in ecology. Nature ecology & evolution 7, 1525-1536 (2023).

Yang, Y. et al. Publication bias impacts on effect size, statistical power, and magnitude (Type M) and sign (Type S) errors in ecology and evolutionary biology. BMC biology 21, 1-20 (2023).

The majority of the introduction were about the data and code sharing/archiving. The author’s paper is about reproducibility but not much relevant literature was mentioned, even those in other fields. Without those research progress, it is hard to judge the significance of the paper.

Methods:

The authors used a very outdated dataset. They searched the meta-analysis papers published between 2015 to 2017, on 20th December 2017 in Scopus abstract and citation database search. I would like to suggest the author to update their data. As the open science is a fast-moving moment in ecology and evolution. What the community wants to know is the “current” reproducibility of meta-analyses or the dynamics of the reproducibility. My intuition is that the data and code sharing and reproducibility of recent 5 years’ meta-analyses are expected to increase a lot. The paper is a meta-scientific work. Information/conclusions disseminated in this paper should not have any misleading elements. Imagine that by roughly reading this paper, an audience will have an impression that the success rate of reproducing ecological and evolutionary meta-analyses is 4-13%. This is totally misleading because the way of calculating success rate (a wrong number used as the denominator!) and the outdated data.

I did not get the point of the Box about “Definitions of data, code, and sharing”. Any researchers should know what are the data, code, and sharing, although they might not know the exact definitions. The aim of the Box is to explain the jargons or terminologies that are not familiar by the general audiences.

Although I tried, but I did not find the code and data that can be used to reproduce the paper itself. Please clearly indicate where it is and provide the publicly accessible repository and link.

Results

What is the point of lines 161 – 163 “The practice of including some kind of supplemental information alongside a published article was very common in this sample. The vast majority (168/177, or 95%) of meta-analysis articles included some kind of supplementary or supporting document (regardless of whether or not they also shared data or code).”. Are these sentences relevant to any aims defined in the introduction of this paper?

As said, the author should have a subsection to show the results of reproducing target results when data are available.

Discussions

The discussions were badly written. A proper way is to put your results in the context of the current literature: compare your results with others, interpret your results, the implications, and limitations. The authors only compared their results to one paper and then have a very general discussion on a “topic” that is not very relevant to the topic of their paper – The widespread use of R in ecology and evolution for meta-analysis. Please rewrite the discussions and have proper comparisons.
